# The role of endogenous versus exogenous sources in the exposome of putative genotoxins and consequences for risk assessment

**DOI:** 10.1007/s00204-022-03242-0

**Published:** 2022-03-06

**Authors:** Ivonne M. C. M. Rietjens, Arand Michael, Hermann M. Bolt, Bourdoux Siméon, Hartwig Andrea, Hinrichsen Nils, Kalisch Christine, Mally Angela, Pellegrino Gloria, Ribera Daniel, Thatcher Natalie, Eisenbrand Gerhard

**Affiliations:** 1 Division of Toxicology, Wageningen University, Stippeneng 4, 6708 WE Wageningen, The Netherlands; 2 Institute of Pharmacology and Toxicology, University of Zurich, Winterthurerstr. 190, 8057 Zurich, Switzerland; 3 Department of Toxicology, Leibniz Research Centre for Working Environment and Human Factors at TU Dortmund (IfADo), Ardeystr. 67, 44139 Dortmund, Germany; 4 ILSI Europe, Av. E. Mounier 83, 1200 Brussels, Belgium; 5 Department of Food Chemistry and Toxicology, Institute of Applied Biosciences (IAB), Karlsruhe Institute of Technology (KIT), Adenauerring 20a, 76131 Karlsruhe, Germany; 6 Food Oils and Fats Research, ADM Hamburg AG, Research, Seehafenstraße 24, 21079 Hamburg, Germany; 7 Department of Toxicology, University of Würzburg, Versbacher Straße 9, 97078 Wurzburg, Germany; 8 Scientific Affairs and Research, Luigi Lavazza SpA, Strada Settimo, 410, 10156 Turin, Italy; 9 Regulatory and Scientific Affairs EMEA, Cargill R&D, Havenstraat 84, 1800 Vivoorde, Belgium; 10 Food Safety, Mondelez International, Bournville Lane, Birmingham, B30 2LU UK; 11 Department of Toxicology and Food Chemistry, University of Kaiserslautern, Kühler Grund 48/1, 69126 Heidelberg, Germany

**Keywords:** Exposome, Endogenous exposure, Genotoxins, Process-related contaminants

## Abstract

The “totality” of the human exposure is conceived to encompass life-associated endogenous and exogenous aggregate exposures. Process-related contaminants (PRCs) are not only formed in foods by heat processing, but also occur endogenously in the organism as physiological components of energy metabolism, potentially also generated by the human microbiome. To arrive at a comprehensive risk assessment, it is necessary to understand the contribution of in vivo background occurrence as compared to the ingestion from exogenous sources. Hence, this review provides an overview of the knowledge on the contribution of endogenous exposure to the overall exposure to putative genotoxic food contaminants, namely ethanol, acetaldehyde, formaldehyde, acrylamide, acrolein, α,β-unsaturated alkenals, glycation compounds, *N*-nitroso compounds, ethylene oxide, furans, 2- and 3-MCPD, and glycidyl esters. The evidence discussed herein allows to conclude that endogenous formation of some contaminants appears to contribute substantially to the exposome. This is of critical importance for risk assessment in the cases where endogenous exposure is suspected to outweigh the exogenous one (e.g. formaldehyde and acrolein).

## Introduction

In modern risk assessment, deriving accurate exposure estimates can present serious challenges and uncertainties. This originates in part from the fact that consumer exposure is multifactorial, including exposure from exogenous environmental, occupational and food-related sources, while in some cases also, endogenous exposure adds to the aggregate exposure. In a seminal definition of the ‘totality’ of human exposure, the term “exposome” was conceived to holistically encompass lifetime and lifestyle factors to provide more solid grounds for scientific ascertainment of associations between multifactorial exposure and human health (Wild 2005). A later definition also included the exposure of the organism through its endogenous metabolism (Miller and Jones 2013). However, given the extreme complexity of the exposome as defined above, it remains a matter of discussion what data sets are appropriate to define the exposome.

For some compounds, it has already been well established that endogenous exposure may add substantially to the total exposure. For instance, methanol and ethanol and their oxidative metabolites formaldehyde and acetaldehyde are ingested with food when consuming fruits and certain beverages but are also continuously formed endogenously during physiological intermediary energy metabolism (Dorokhov et al. 2015; Ostrovsky 1986). Thus, endogenous exposure constitutes a substantial intrinsic part of the total exposure.

It came as a surprise when it was found that several process-related contaminants (PRCs) are not only formed in foods upon heating or because of other process conditions, but also may be generated endogenously in the organism as physiological components of metabolic pathways in the host, and/or may originate from the intestinal microbiome.

PRCs are formed in foods upon processing, encompassing any type of the multifaceted technical or household-related processes applied to food. The present review discusses those environmental compounds and PRCs that also occur as physiological components of mammalian intermediary energy metabolism or other processes in the organism, including generation by the host microbiome. The sequence of chapters intends to reflect the strength of scientific evidence underpinning the relevance of endogenous exposure as a constitutive part of total exposure to a given compound.

This dual exposure from endogenous and exogenous sources constitutes the rational for the selection of compounds for this review. Thus, the list starts with ethanol, acetaldehyde and formaldehyde, followed by acrylamide, acrolein and further α,β-unsaturated alkenals, as well as glycation products. Next, the *N*-nitroso compounds (NOC) are discussed here within a condensed summary of the extensive published evidence accumulated over the last 50 years. This is followed by ethylene oxide for which there is compelling evidence for endogenous exposure, although exposure may primarily occur by inhalation in the (working) environment. Finally, PRCs for which the evidence of endogenous occurrence at present is not as compelling as for the aforementioned compounds, or not (yet) available are discussed subsequently. This group includes furans, glycidol-esters, and the chloropropanols, as examples.

This review aims to provide an overview of the state-of-the-art with respect to understanding the contribution of endogenous exposure to the overall exposure to putative genotoxic food contaminants. This may also be of potential use for other sectors of the overall exposome. Assessing how much of a compound is generated through endogenous processes can be challenging, since the underlying pathways are often only partially elucidated and may be influenced by the physiological homeostasis reflecting individual health-, age- and gender-related parameters. Nevertheless, in cases where endogenous exposure is proven to add substantially to the exposome, it becomes essential to better understand the contribution of in vivo background occurrence, as compared to the ingestion from exogenous sources, to arrive at a comprehensive risk assessment.

Perspectively, this approach may pave the way to a more holistic risk assessment that will include the human endogenous background exposure where this appears to provide an important contribution to the exposome. In consequence, this may lead to a refinement of human health risk assessment by analysing the total exposome and the respective contributions of endogenous and exogenous origin. Further, the endogenous exposome, if adequately explored, may become an established reference point (point of departure) against which to evaluate exogenous exposure in human health risk assessment.

## Process-related contaminants

### Ethanol

Ethanol (ethyl alcohol) is an alcohol produced naturally by fermentation of sugars. It can be found in overripe fruits and is present in alcoholic and, to a minor extent, in “non-alcoholic” beverages. For example, grape juice and other fruit juices can contain up to 1% ethanol. The existence of a disease called “auto-brewery syndrome”, also named “gut fermentation syndrome” or “drunkenness disease”, already indicates that endogenous formation of ethanol may occur within the digestive tract, in some cases even at levels causing symptoms of alcohol intoxication. This endogenous ethanol formation has been ascribed to the fermentation of ingested carbohydrates by the gut microbiota (Painter et al. 2020).

#### Characterisation, formation, occurrence and public health concern (ethanol)

Ethanol has been the subject of numerous human safety evaluations by the International Agency for Research on Cancer and the Joint FAO/WHO Expert Committee on Food Additives (IARC 1988, 2010a, 2018; JEFCA 1970). In these assessments, exposure to ethanol has been associated with adverse effects on the liver, the cardiovascular system, the central nervous system, and the induction of cancers of the digestive tract, larynx, breast and liver. IARC concluded that ethanol shows weak mutagenic potential in standardised in vitro and in vivo test systems. The mutagenicity of ethanol may result from the DNA reactivity of its metabolite acetaldehyde but has also been related to the ethanol-induced production of reactive oxygen species, for example, via the induction and activity of inflammatory cytokines or cytochrome P4502E1 (IARC 2018). JEFCA concluded that the use of ethanol as an extraction solvent should be restricted to usage determined by good manufacturing practice, in which case residues are unlikely to have any toxicological effects and that ethanol posed no safety concern at the levels of intake resulting from use of ethyl esters as flavouring agents (JEFCA 1970). The German Research Foundation (DFG) MAK commission has rated ethanol and acetaldehyde as class 5 carcinogens (Hartwig and MAK commission 2018). This class comprises compounds that cause or are considered to cause cancer in humans or animals for which a MAK value can be derived. This follows the rationale that although a genotoxic mode of action is conceived of prime importance, ethanol exposure is considered to contribute only very slightly to human cancer risk, provided the MAK and BAT values are respected (DFG 2020; Nakamura et al. 2014). Given the role for acetaldehyde in the carcinogenicity of ethanol, it is also relevant to note that in the human population a polymorphism for aldehyde dehydrogenase 2 (ALDH2) is expected to have a major impact on the ultimate adverse outcome of ethanol exposure. This is discussed to a further extent in “Acetaldehyde”.

#### Existing knowledge on exogenous sources

##### Exogenous exposure from food

Exogenous exposure to ethanol occurs via food with a major contribution for adults coming from alcoholic beverages (Health Council of the Netherlands 2006). In 2018, IARC presented estimates of annual ethanol intake from alcoholic beverages for the adult population (> 15 years of age) varying from 0.7 L per capita for the Eastern Mediterranean Region to 12.2 L per capita for the European regions in the period 2003–2005 (IARC 2018). A standard measure of an alcoholic drink contains roughly 8–14 g ethanol. For example, in the UK, the National Health Service (NHS) has defined 1 alcohol unit as 8 g of ethanol (NHS 2018). In addition, fruit juices may contain notable levels of alcohol. For example, 0.1–1 g/L ethanol was found in a random selection of non-fermented commercial fruit juices, within the legal limits for fruit juice in general (0.38%) or grape juice (1%) (Gorgus et al. 2016).

##### Exogenous exposure from other sources

Exposure to ethanol may also occur in the occupational setting mainly by inhalation and, especially when used as an antiseptic agent, by dermal absorption. Dermal exposure may also result from ethanol-containing cosmetics, perfumes and drugs used topically. The Dutch Health Council estimated that inhalation exposure of ethanol under working conditions for a person inhaling 10 m^3^ of breath volume per working day with an atmospheric content of 1900 mg ethanol/m^3^, assuming 60% absorption efficiency, would result in an exposure of 11.4 g of ethanol (Health Council of the Netherlands 2006). This estimate is considerably higher than what would result from exposure by inhalation to ethanol at the German MAK value of 380 mg/m^3^.

#### Existing knowledge on endogenous sources

Ethanol is known to be present in blood, breath, and urine of healthy subjects (Liebich et al. 1982). Its endogenous formation may result from microbial fermentation by the gut microbiota, or from its formation in pathways endogenous to host metabolism including reactions producing acetaldehyde, a metabolite that may be reduced to ethanol to some extent. Examples of such reactions are the conversion of threonine to glycine and acetaldehyde or of deoxyribose to glyceraldehyde and acetaldehyde (Krebs and Perkins 1970). This implies that endogenous formation of ethanol and acetaldehyde are linked, as will also become clear from “Acetaldehyde”.

The mean level of ethanol in human blood or plasma has been estimated to amount to 0.27 (± 0.17) mg/L blood (Health Council of the Netherlands 2006; Sprung et al. 1981), and to 1–2 mg/L plasma (Liebich et al. 1982). Based on the volume of fluid per kg of body weight (0.54 L/kg bw), this implies an amount of 10.2–75.6 mg ethanol for a 70 kg person, equal to 0.15–1.08 mg ethanol/kg bw (Caldwell 1989). Plasma levels of unexposed people are reported in a range of 0.1–0.3 mg/L plasma, as measured by gas chromatography (GC)/mass spectroscopy (MS). This would make up to around 65–200 µg/kg bw, when based on the average distribution factor of 0.65, corresponding to ≤ 10 mg/kg/day (Ostrovsky 1986).

#### Available biomarkers

Biomarkers for detecting recent alcohol exposure are ethanol and its metabolites, ethylglucuronide and ethylsulfate, that can be measured in blood or urine. Given the rapid metabolism and clearance of these metabolites, detection of chronic exposure may require other biomarkers and/or matrices. These include phosphatidylethanol generated by the reaction of phosphatidylcholine with ethanol catalysed by phospholipase D and fatty acid ethyl esters resulting from esterification of fatty acids and ethanol. Hair and nails have been used as alternative matrices for analysis of the metabolites, offering longer detection windows and easier sample collection (ARUP Consult 2020; Shu 2016).

#### Impact of endogenous formation on risk assessment

From the overview presented above, it appears that endogenous formation of ethanol may add substantially to the overall exposure. For people not consuming alcoholic beverages and not being exposed via the occupational setting, endogenous exposure may even present a major source. In risk assessment, this endogenous formation may be used as a reference against which to evaluate exogenous exposure. For example, exposures resulting from use of ethyl esters as flavour constituents have been deemed not to add substantially to the overall exposure, thus not raising a safety concern (JEFCA 1970).

Another example of using this approach in risk assessment can be found in the evaluation of exposure to ethanol in the occupational setting by the Health Council of the Netherlands (Health Council of the Netherlands 2006). By comparison with the endogenous ethanol concentration in human blood of 0.27 (± 0.17) mg/L, which corresponds to an AUC for 80 years of 21.6 (± 13.6) (mg/L) × year, an occupational exposure of 13 mg/m^3^, resulting in an AUC of approximately 0.2 (mg/L) × year, was concluded to be negligible. The same committee further calculated an additional breast cancer risk of 4/10^3^ after 40 years exposure towards 1300 mg/m^3^. In addition, the German MAK commission considered that—based on the variation of the endogenous exposure—an occupational exposure of 200 mL/m^3^ (380 mg/m^3^) would be within the standard deviation of the lifetime AUC and thus would contribute only little to the cancer risk (Hartwig and MAK commission 2018).

### Acetaldehyde

Exposure to acetaldehyde can occur via various sources, both endogenous and exogenous. Acetaldehyde is used to produce basic chemicals, thus giving rise to occupational exposure by inhalation. In addition, acetaldehyde is the major and most critical metabolite of ethanol and is also present in cigarette smoke; therefore, oral as well as inhalation exposure are also relevant for the general population. Genetic polymorphisms within the ALDH2*2 gene, coding for the enzymes responsible for the oxidation of acetaldehyde to acetate, have major impact on internal exposure levels as well as on carcinogenicity. Endogenous sources arise from physiological amino acid metabolism and from other metabolic processes as well as from metabolism by the intestinal and salivary microbiomes.

#### Characterisation, formation, occurrence and public health concern

Acetaldehyde is the primary oxidative metabolite of ethanol, and its formation appears to be the major mechanism of ethanol-associated cancer in the upper aerodigestive tract upon chronic alcohol consumption (Brooks and Theruvathu 2005; Yu et al. 2010) (see also “Ethanol”). Thus, based on epidemiological data, IARC concluded that there is sufficient evidence in humans for the carcinogenicity of alcohol consumption and for the carcinogenicity of acetaldehyde associated with the consumption of alcoholic beverages, and both were classified in Category 1 (IARC 2010a, 2012b). In addition, in several animal studies after oral administration of ethanol, increased incidences of cancers of the head and neck and the liver and benign tumours of several organs in rats and liver tumours and mammary gland adenocarcinomas were found, e.g. in mice (IARC 2012b). Regarding acetaldehyde itself, carcinogenicity in the nose and larynx of rats and hamsters was observed upon inhalation exposure. Several tumours were also seen after oral exposure, but without clear dose–response relationship. As the underlying mechanism of carcinogenicity, acetaldehyde has been shown to be genotoxic in vitro, giving rise to elevated levels of sister chromatid exchanges (SCE), chromosomal aberrations, micronuclei, and gene mutatio﻿ns. The induction of SCE and micronuclei was also evident in vivo (IARC 2012b; MAK Commission 2013).

**Fig. 1:**
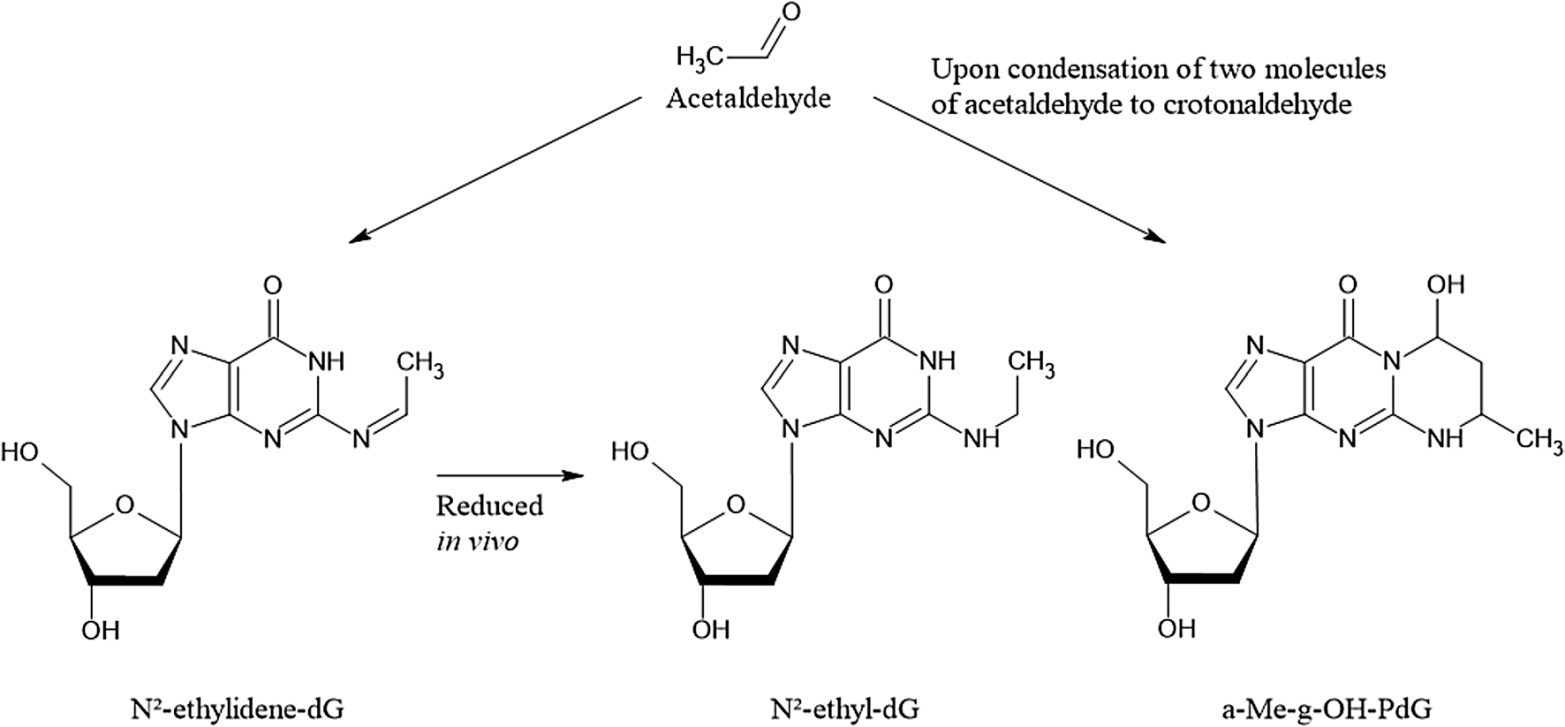
Acetaldehyde-derived DNA adducts; for details and references see text

Several DNA adducts involving the exocyclic amino group of deoxyguanosine are formed in vitro and in vivo after exposure to acetaldehyde (Brooks and Zakhari 2014; Yu et al. 2010) (Fig. 1). Upon reaction of a single molecule of acetaldehyde, *N*^2^-ethylidene-2′-deoxyguanosine (*N*^2^-ethylidene-dG) is formed, which can be reduced in vivo to the more stable *N*^2^-ethyldeoxyguanosine (*N*^2^-ethyl-dG). Apparently, this adduct is not repaired in vivo, which makes it an excellent biomarker of exposure, but its mutagenic potential appears to be low [reviewed in (Brooks and Zakhari 2014)]. Further DNA lesions are formed upon the condensation of two molecules of acetaldehyde to crotonaldehyde, namely *R*- and *S*-*α*-methyl-*γ*-hydroxy-1,*N*^2^-propano-2′-deoxyguanosine (*α*-Me-*γ*-OH-PdG; 1,*N*^2^-PdG) which are considered responsible for the mutagenic, genotoxic and carcinogenic properties of crotonaldehyde (Eder and Budiawan 2001) and thus also acetaldehyde (Brooks and Zakhari 2014). Furthermore, the ring-opened form of 1,*N*^2^-PdG is a precursor lesion for the formation of DNA–protein or DNA–DNA crosslinks (Yu et al. 2010). Both nucleotide excision repair as well as homologous recombination mediated by the Fanconi Anemia-Breast Cancer Susceptibility network are involved in the repair of the respective DNA lesions, and especially defects in the latter pathway appear to be also involved in developmental toxicity of acetaldehyde (Brooks and Zakhari 2014). However, high concentrations of acetaldehyde are required to induce 1,*N*^2^-PdG in vitro, and only low levels below the limit of quantification were detected in the brain and lungs of (^13^C_2_)-acetaldehyde-exposed animals upon inhalation (Sanchez et al. 2018). Therefore, it needs to be further investigated whether relevant levels of this DNA lesion are formed in vivo (MAK Commission 2013). In addition to these and further types of DNA lesion, acetaldehyde can also impact the function of the nuclear genome indirectly, via interactions with specific DNA repair enzymes such as O^6^-methylguanine methyltransferase and enzymes involved in epigenetic gene regulation such as DNA cytosine methyltransferase (Brooks and Zakhari 2014). Therefore, genomic stability can be compromised by acetaldehyde via different, most likely complementing mechanisms.

Acetaldehyde, produced by ethanol oxidation, is rapidly metabolised mainly by mitochondrial ALDH2 to form acetate and NADH. ALDH2-deficient animals are more susceptible to the generation of *N*^2^-ethyl-dG adducts after oral ethanol uptake and inhalation exposure to acetaldehyde. In agreement with this observation, ALDH2*2 carriers have dramatically elevated rates of esophageal cancer from alcohol drinking compared with individuals with fully active ALDH2 who drink comparable amounts of alcohol (Brooks and Zakhari 2014).

#### Existing knowledge on exogenous sources

##### Exogenous exposure from food and beverages

Acetaldehyde is the major metabolite from ethanol; therefore, the exposure to acetaldehyde correlates with the consumption of alcoholic beverages. Usually, the concentration of acetaldehyde remains more than three orders of magnitudes below that of ethanol (low micromolar against up to millimolar range), due to the large capacity of ALDH2; however, in case of polymorphisms within the ALDH2*2 gene, concentrations of acetaldehyde may be considerably higher (Umulis et al. 2005). In addition, alcoholic beverages themselves contain significant levels of acetaldehyde (Hartwig et al. 2020).

##### Exogenous exposure from other sources

Acetaldehyde is primarily used as an intermediate in the manufacturing of numerous products, including acetic acid, flavourings, aniline dyes, plastics, and synthetic rubber. Thus, occupational exposure is a relevant exposure source. Furthermore, acetaldehyde is a ubiquitous indoor and outdoor air pollutant, deriving from industrial burning processes, traffic emissions, and combustion of wood. It is also a component of tobacco smoke. Therefore, inhalation is a relevant route of exogenous exposure.

#### Existing knowledge on endogenous sources

Besides exposure via its occurrence in food, beverages, cigarette smoke, and exposure at the workplace, acetaldehyde is also produced endogenously in the intermediary metabolism by oxidative decarboxylation of pyruvate, in the course of amino acid metabolism, and by other metabolic processes. It is also formed by intestinal and salivary microflora. Endogenous concentrations were found to be 2.2–3.6 µM (about 0.1 mg/L) in blood (MAK Commission 2013). A relevant source of endogenous acetaldehyde exposure consists of the metabolism of ethanol to acetaldehyde by oral microbes and mucosal cells. Because of inefficient local enzymatic detoxification, acetaldehyde accumulates in saliva and gastric juice (MAK Commission 2013). Salivary acetaldehyde concentrations were found to be much higher than the blood acetaldehyde concentrations after ingestion of alcoholic beverages (Homann 2001). In addition, deficient ALDH2 activity plays an important role in increasing the risk for upper digestive tract cancer, and concentrations of acetaldehyde in saliva and gastric juice are 2 times and about 5 times higher in ALDH2-deficient compared to ALDH2-proficient persons, respectively (Lachenmeier and Salaspuro 2017; Yokoyama et al. 2008). Thus, regarding risk assessment of ethanol and acetaldehyde, local concentrations and effects need to be considered in preference to systemic effects (Maejima et al. 2015).

#### Available biomarkers

The two primary DNA lesions described above are applied as biomarkers of exposure, namely *N*^2^-ethylidene-dG and 1,*N*^2^-propano-dG, generated by direct reaction of acetaldehyde with the *N*^2^ position of 2′-deoxyguanosine or with two molecules of acetaldehyde, as described above. Since *N*^2^-ethylidene-dG is unstable at the nucleotide level, it requires reduction to form the more stable *N*^2^-ethyl-dG for analysis (Balbo et al. 2016). To discriminate between endogenous formation of DNA adducts and those formed via exogenous exposure to acetaldehyde, methods applying stable isotopes have been established. Thus, studies with human lymphoblastoid TK6 cells using (^13^C_2_)-acetaldehyde in the range of 50 nM to 2 mM, incubated for 12 h, revealed an increase in exogenous *N*^2^-ethylidene-dG formation (after reduction to *N*^2^-ethyl-dG) at exposure concentrations ≥ 1 μM, whereas the endogenous adducts remained nearly constant across all exposure concentrations, with an average of 6.6 adducts/10^8^ nt. Levels of exogenous adducts were lower than endogenous adducts at concentrations ≤ 10 μM and exceeded their level at concentrations ≥ 250 μM. The sum of endogenous and exogenous adducts reached a statistically significant increase over the endogenous background at 50 µM. Statistically significant decreases in cell survival and increases in micronucleus formation occurred at ≥ 1000 μM acetaldehyde (Lachenmeier and Sohnius 2008; Moeller et al. 2013).

Dose-dependent increases in DNA adduct levels in vitro and in vivo were observed in several studies for *N*^2^-ethyl-dG adducts (Balbo et al. 2016). However, the biological significance of identified DNA adducts for mutagenicity and carcinogenicity of acetaldehyde is still not fully elucidated, and respective dose-dependent correlations are required. Furthermore, even though any additional intake from exogenous sources within the range of variation of the endogenous body burden will add little to cancer risk, respective contributions should be verified on the level of local DNA adducts in vivo, for instance in tissues of first contact. Such local dosimetry needs to take into account the effects of ALDH2-deficiency, since local concentrations of acetaldehyde and thus levels of acetaldehyde-derived DNA adducts, for example in the upper aerodigestive tract, seem to play a major role in the development of cancer from ethanol and acetaldehyde. Additional local effects such as tissue irritation by acetaldehyde may be promotional for its carcinogenic effect which needs to be further elucidated.

#### Impact of endogenous formation on risk assessment

When performing risk assessment from exogenous sources, DNA lesions induced by endogenous sources should be considered for comparison. This approach was chosen by the MAK Commission that classified ethanol and acetaldehyde as Category 5 carcinogens (see “Characterization, formation, occurrence and public health concern (ethanol)”). With respect to internal exposure, air levels at the MAK value of 50 mL/m^3^ (≙ 91 mg/m^3^) were estimated to lead to an additional body burden in the range of variation of the lifetime endogenous body burden. Nevertheless, local tumours in rats occurred in the olfactorial epithelium, and prevention of nasal tissue irritation was also considered for the derivation of the MAK value for acetaldehyde (Neumann et al. 1998). This approach has also been discussed in more detail by the German MAK commission (MAK Commission 2013).

The German MAK commission classified acetaldehyde in Group 5, based on irritation and thus accelerated cell proliferation as tumour-promoting factor and defined a MAK value which protects from carcinogenicity. The latter approach has been supported by recent developments in the quantification of both endogenous and exogenously induced DNA adducts as detailed above. While DNA damage is detectable already at low dose levels upon inhalation in the nasal epithelium of rats, respective levels are considerably lower than endogenously induced DNA lesions.

### Formaldehyde

#### Characterisation, formation, occurrence and public health concern

Formaldehyde exposure occurs via exogenous sources from food, consumer products, and at workplaces. However, it is also formed endogenously during amino acid metabolism and as a major constituent of the physiological C-1-pool. Since formaldehyde is the metabolite of methanol that raises concern, the text on formaldehyde also relates to methanol. Formaldehyde induces DNA damage and is carcinogenic, inducing squamous cell carcinoma in the nose in experimental animals upon inhalation and—with less evidence—nasopharyngeal carcinomas in humans. Formaldehyde can induce DNA adducts including *N*^2^-hydroxymethyl-dG, *N*^6^-hydroxymethyl-dA, as well as *N*^4^-hydroxymethyl-dC and, in turn, DNA protein crosslinks. Those DNA adducts are considered promutagenic, as the amino groups participating in Watson–Crick base pairing are involved and DNA protein crosslinks are formed which give rise to double strand breaks (Lai et al. 2016; Swenberg et al. 2011). Furthermore, both intra- and inter-strand DNA crosslinks are generated (Kawanishi et al. 2014). Formaldehyde was classified by IARC as a human carcinogen (Category 1) (IARC 2012a). Due to its high chemical reactivity, formaldehyde causes local irritation as well as acute and chronic toxicity after direct contact in target tissues, which may increase the carcinogenic risk. Thus, based on the mode of action, formaldehyde was classified in Category 4 for carcinogens by the German MAK commission (Greim 2002), i.e. carcinogenic substances for which an increase in cancer risk is not expected provided that the MAK value is observed.

#### Existing knowledge on exogenous sources

Exposure towards formaldehyde is very common both for consumers as well as for workers in different industrial settings. Formaldehyde is predominantly used as a chemical intermediate in the production of formaldehyde-based resins, which are widely used as adhesives and binders in the woodworking, paper as well as synthetic vitreous fibre industries, in the production of plastics and in textile finishing. Formaldehyde is also used as an intermediate in the manufacture of industrial chemicals. Furthermore, in aqueous solution called formalin, it is widely used as a disinfectant and preservative (IARC 2012a). This is associated with exposure at workplaces during production, but also relevant for the general population due to formaldehyde release from the respective products.

##### Exogenous exposure from food

Levels of formaldehyde in different foods have been summarised by the European Food Safety Authority (EFSA 2014). Highest levels are found in meat, poultry, and fish, but also in fruit, vegetables, and coffee. However, levels in food are variable and range from values below 1 mg/kg in milk to more than 200 mg/kg in some fish species. Although a precise assessment of dietary human exposure is not possible, EFSA assumed that—based on the consumption of one kg of food—formaldehyde uptake would not exceed 100 mg/day, corresponding to 1.7 or 1.4 mg/kg bw for a 60 or 70 kg person, respectively. Formaldehyde is also derived from metabolism of methanol. Methanol occurs naturally in food as free methanol, methyl esters of fatty acids or methoxy groups in polysaccharides such as pectin, from which it is released during digestion. Major sources are fruits, vegetables, and fresh fruit juices (Lindinger et al. 1997).

Moreover, substantial amounts of methanol may be present in alcoholic beverages. Other sources may be food additives such as the preservative dimethyl dicarbonate in amounts of up to 250 mg/L in non-alcoholic beverages, which decomposes rapidly to carbonate and methanol (EFSA 2015b), as well as aspartame, where an estimated 10% is metabolised to methanol and subsequently to formaldehyde, formic acid or formate (EFSA 2013).

##### Exogenous exposure from other sources

Non-occupational and non-food-derived sources of exposure to formaldehyde include combustion processes, e.g. through emissions from motor vehicles, power plants, incinerators, refineries, wood stoves, and kerosene heaters. In addition, formaldehyde may be released—among other sources—from building materials, carpets and paints, and during its use as a disinfectant. It is also present in tobacco smoke. Concentrations of formaldehyde in outdoor air are generally below 0.001 mg/m^3^ in remote areas and below 0.02 mg/m^3^ in urban areas. Indoor air levels in houses are between 0.02 and 0.06 mg/m^3^; indoor combustion sources can significantly increase these levels. Cigarettes may contribute as much as 10–25% of the indoor exposure (IARC 2012a). The WHO Guideline for Indoor Air Quality for formaldehyde (WHO 2010) set an exposure limit to 0.1 mg/m^3^ (30-min average concentration), considered to be protective against both acute and chronic sensory irritation in the airways of the general population, including sensitive subpopulations.

#### Existing knowledge on endogenous sources

Formaldehyde is an endogenously formed metabolic intermediate present in all cells at an intracellular concentration of around 400 µM. Predominant sources are the methanol and amino acid metabolism, the one-carbon pool, lipid peroxidation and P450-dependent demethylation (Dhareshwar and Stella 2008; Nakamura et al. 2014; Swenberg et al. 2011). Due to its electrophilicity, formaldehyde is reactive towards a variety of cellular components such as glutathione (GSH), proteins and nucleic acids and folic acid. The metabolism of formaldehyde is rapid and catalysed by GSH-dependent formaldehyde dehydrogenase (also known as alcohol dehydrogenase 5, ADH5) and *S*-formyl-GSH hydrolase to result in the formation of formic acid. Formic acid then enters the one-carbon pool where it can be incorporated as a methyl group into nucleic acids and proteins and is either excreted in the urine or oxidised to carbon dioxide and exhaled at a significantly slower rate than its formation from formaldehyde (formic acid half-life in plasma is between 1 and 6 h) (Dhareshwar and Stella 2008; EFSA 2014).

#### Available biomarkers

**Fig. 2:**
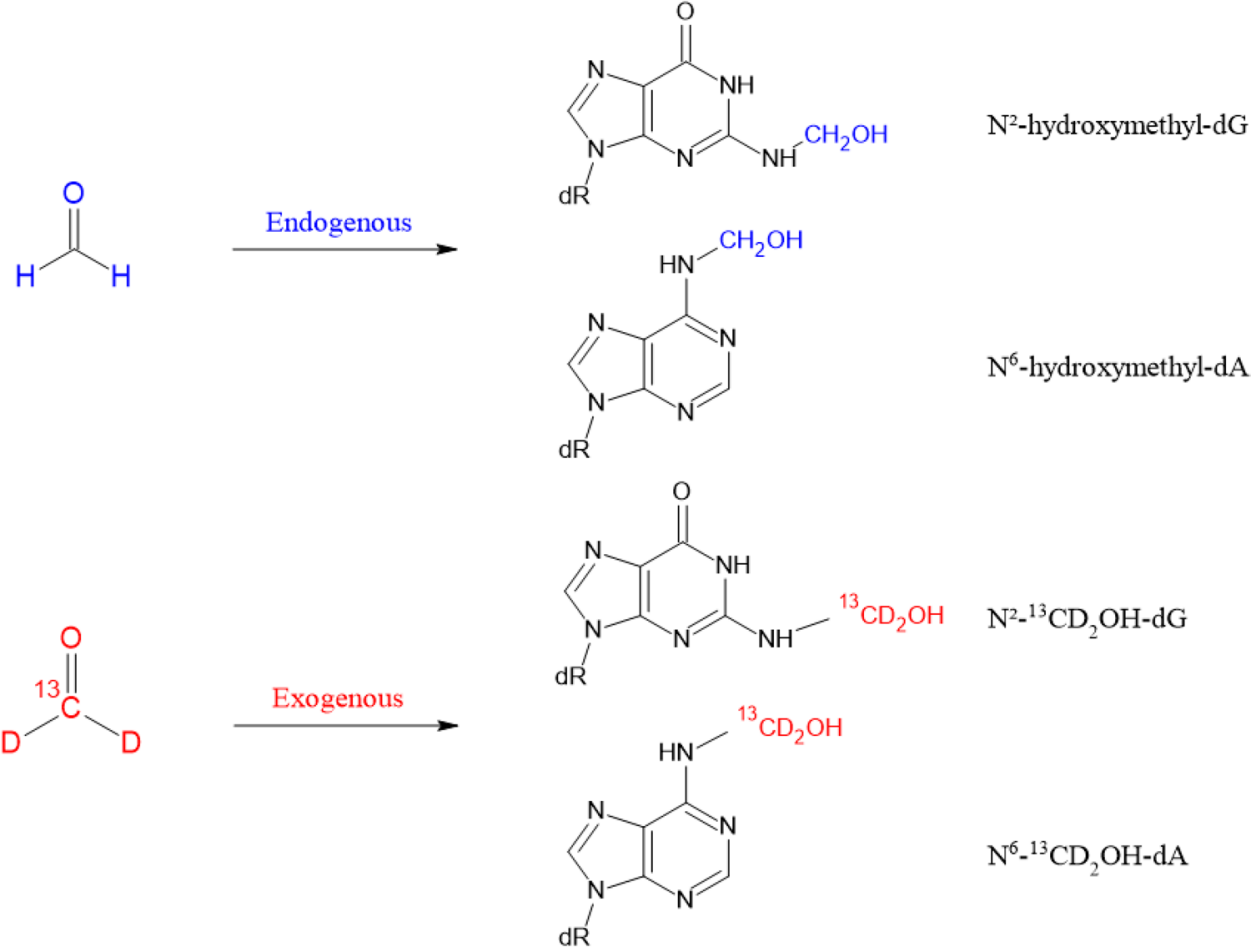
Formation of hydroxymethyl DNA adducts induced by formaldehyde and discrimination between endogenous and exogenous sources (Adapted from Swenberg et al. 2011)

Formaldehyde-induced DNA lesions have been shown to be not only suitable biomarkers for formaldehyde exposure, but also to discriminate between its endogenous formation and additional DNA damage generated via exogenous exposure (Fig. 2). Available quantification methods cover DNA base lesions as well as DNA–protein and DNA–DNA crosslinks. DNA–protein crosslinks and DNA–DNA crosslinks were detected in rats after 6 h inhalation of 0.3 ppm ^14^C-formaldehyde (Casanova et al. 1989) and in monkeys starting from 0.7 ppm under the same conditions (Casanova et al. 1991), reviewed in several articles (Greim 2002; McGregor et al. 2010).

As one significant step forward, Swenberg and coworkers (2011) were able to distinguish between *N*^2^-hydroxymethyl-dG adducts resulting from endogenously and exogenously formed formaldehyde using stable isotopes (^13^CD_2_-formaldehyde, combined with MS analysis).

**Fig. 3:**
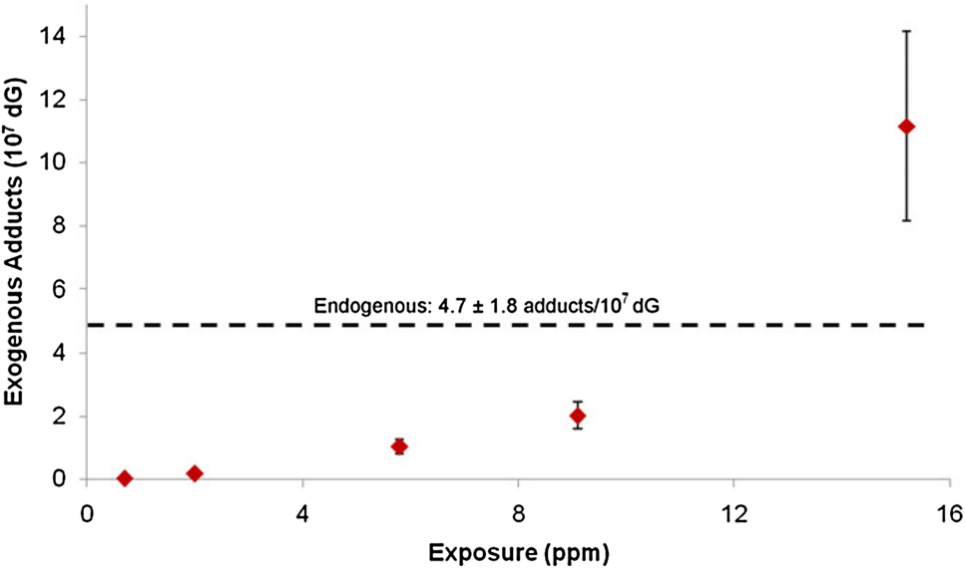
Molecular dosimetry of *N*^2^-hydroxymethyl-dG adducts in the nasal epithelium of rats exposed to formaldehyde via inhalation (6 h) (Adapted from Swenberg et al. 2011)

The steady-state level of DNA base damage derived from endogenous formaldehyde generation in rats as determined by liquid chromatography (LC)–MS/MS was found to be about 1–7 adducts/10^7^ dG in case of *N*^2^-hydroxymethyl-dG and 1–3 adducts/10^7^ dA in case of *N*^6^-hydroxymethyl-dA (Lu et al. 2010; Swenberg et al. 2011). The same order of magnitude was reported for human leukocytes (0.7 *N*^6^-hydroxymethyl-dA/10^7^ dA) (Wang et al. 2009). To discriminate between DNA adducts formation resulting from endogenous and exogenous formaldehyde, DNA adducts were determined in the nasal epithelium of rats exposed to formaldehyde for 6 h via inhalation. As shown in Fig. 3, DNA adducts induced by exogenous formaldehyde were found even at the lowest concentration (0.7 ppm) and increased dose-dependently. However, the level of DNA lesions resulting from endogenous formaldehyde exposure largely was predominant and was exceeded by the DNA adducts resulting from exogenous formaldehyde only after exposure concentrations of 10 ppm and above (Swenberg et al. 2011). Similarly, DNA adducts resulting from exogenous formaldehyde inhalation were found in the nasal respiratory epithelium in the rat after longer exposure (28 days, 2 ppm) as well as in a monkey study (2 days, 6 ppm), but not in any other tissue distant to the site of initial contact (Yu et al. 2015). Lower concentrations of 0.001, 0.03, 0.3 ppm [^13^CD_2_]-formaldehyde (28 days nose-only inhalation, 6 h/day) yielded no detectable exogenous DNA lesions or DNA–protein crosslinks in any tissue sample, including the most susceptible nasal epithelium, while endogenous adducts were present in all analysed tissues (Leng et al. 2019). The absence of additional exogenous DNA lesions up to 0.3 ppm formaldehyde in this experimental setting was also strengthened by a recent refined modelling approach (Campbell et al. 2020).

#### Impact of endogenous formation on risk assessment

Endogenous vs. exogenous exposure towards formaldehyde has been investigated both for oral exposure via food as well as for inhalation exposure, particularly relevant for workplace exposure.

EFSA estimated the relative contribution of formaldehyde from food vs. the endogenous production. They considered three different sources, namely formaldehyde in different food products, carry over from animals fed with formaldehyde-supplemented feed and oral exposure to formaldehyde from aspartame-derived methanol at the currently acceptable daily intake. Among these sources, the highest potential exposure was derived from aspartame, followed by food products and only very little impact was calculated for carry over from animal feed. Even when combining all three sources, less than 1% compared to endogenous exposure was calculated (EFSA 2014).

Regarding inhalation as the most relevant route of workplace exposure, formaldehyde induces nasal tumours with a sublinear dose–response relationship. While IARC—based on hazard—assigned formaldehyde to Group 1 (carcinogenic to humans), the German MAK commission classified it in Group 4, based on irritation and thus accelerated cell proliferation as tumour-promoting factor and defined a MAK value which protects from carcinogenicity. The latter approach has been supported by recent developments in the quantification of both endogenous and exogenously induced DNA adducts as detailed above. While DNA damage is detectable already at low dose levels upon inhalation in the nasal epithelium of rats, respective levels are considerably lower than endogenously induced DNA lesions. With respect to the nose as the critical target organ for nasal tumours, it is unclear whether inhaled formaldehyde at the low dose levels reaches the basal cells as target cells at all or whether it undergoes rapid clearance, metabolism and/or reaction in the upper cell layers. To clarify this aspect, further research is required. In addition to DNA damage, accelerated cell proliferation is needed for relevant conversion of DNA lesions into mutations. In this context, data from animal experiments analysing gene expression profiles revealed a transcriptional benchmark dose (BMD) at the mRNA level of 1 ppm for significantly changes in sensitive response genes associated with cellular stress, inflammation, and cell proliferation [summarised in (Hartwig et al. 2020)]. Those data are in agreement with the irritation of the eye or nose and throat in human volunteers observed at 0.5 or 1 ppm. Therefore, considering the currently available database, it can be assumed that below the level of irritation, there is no relevant additional risk of nasal tumours at the low dose level, such as exposure at the MAK value of 0.3 ppm (0.37 mg/m^3^) (Greim 2002). Furthermore, the absence of additional DNA lesions distant to the site of initial contact contradicts the potential induction of leukaemia by formaldehyde (Lu et al. 2010; Yu et al. 2015).

### Acrylamide

#### Characterisation, formation, occurrence and public health concern

**Fig. 4:**
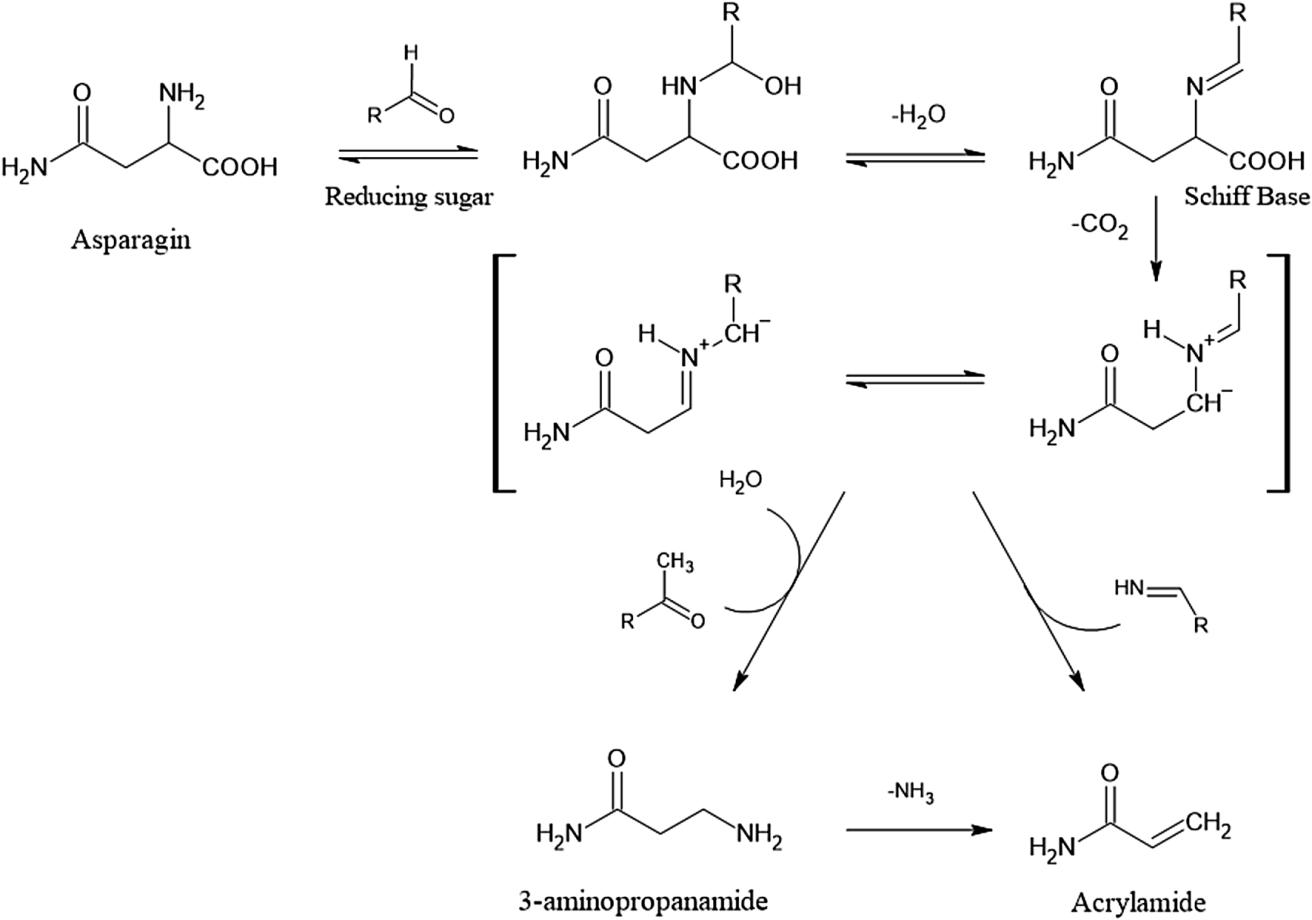
Heat-induced acrylamide formation in food

Acrylamide (AA) is an α,β-unsaturated carbonyl compound used as an industrial chemical, for instance in the production of polyacrylamides. As such, it finds broad industrial use in the form of various polymers with extensive technical applications. In 2002, the discovery that AA is generated during heat processing of foods as a PRC has raised great public attention. Extensive research was devoted to unravelling the formation pathways and to develop risk management measures to counteract thermally induced AA formation. It is widely accepted that in foods at temperatures exceeding ~ 120 °C, reaction rates of carbonyl groups of reducing carbohydrates with alfa-amino groups of the second main precursor molecule, the amino acid amide asparagine, increase to initiate enhanced Schiff base formation. This is the initial step of a sequence of chemical transformations leading to AA (Fig. 4). Such reactions are examples of the Maillard reactions, common to heat-induced food browning, and generate a wide spectrum of thermally induced compounds termed Maillard reaction products. The sequence of chemical reactions in Fig. 4 shows the predominant formation pathway of AA in foods (Guth et al. 2013).

Dietary intake is considered the major path of consumer exposure, at levels estimated in general rarely to exceed about 3–4 µg/kg bw and to amount to average levels of < 1 µg/kg bw (EFSA 2015a; Guth et al. 2013).

AA itself is not mutagenic or carcinogenic, except for exceedingly high exposure levels in a toxic dose range. After absorption, AA is extensively metabolised. First, due to its Michael reactivity, AA can directly react with cysteine moieties and other nucleophilic centres in structural and/or plasma proteins and with molecules such as *N*-acetyl cysteine or GSH (GSH). Reactions with GSH may occur spontaneously and/or by enzymatic catalysis by GSH-S-transferases. The high reactivity of AA towards nucleophilic centres in proteins is conceived to be primarily causative for the potent neurotoxicity it exerts in animals and humans, resulting in adverse effects on the peripheral nervous system, with histopathological changes in nerves and nervous system structures (EFSA 2015a).

A second major biotransformation of AA consists of preferentially cytochrome P450 2E1 (CYP 2 E1)-mediated epoxidation. The resulting metabolite, 2,3-epoxypropanamide, also called glycidamide (GA), is considered the ultimate genotoxic metabolite of AA, supposed to damage DNA by covalent binding to nucleophilic centres, primarily at the nitrogen in position 7 of the DNA base guanine, an adduct considered of rather low promutagenic activity (Eisenbrand 2020).

**Fig. 5:**
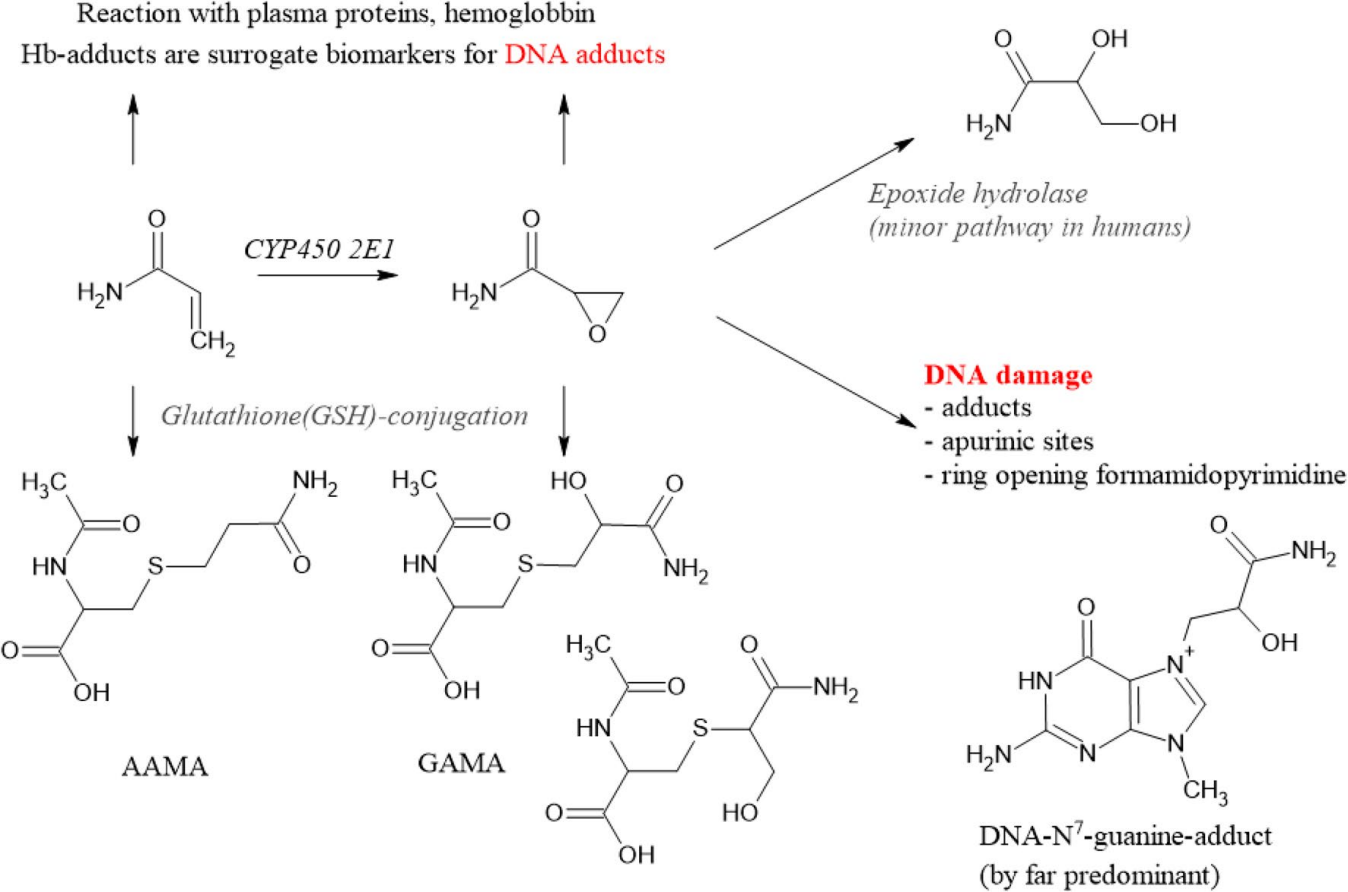
Biotransformations of acrylamide. *AAMA* *N*-acetyl-*S*-(2-carboxamidoethyl) cysteine, *GAMA* isomeric GA *N*-acetylcysteine adducts, *N*-acetyl-*S*-(3-amino-2-hydroxy-3-oxopropyl)-l-cysteine) and *N*-acetyl-*S*-(1-carbamoyl-2-hydroxyethyl)-l-cysteine

Like AA, GA can also react with GSH directly by covalent binding to its thiol group and/or mediated enzymatically by GSH-S-transferases (Fig. 5). The resulting primary GSH adducts undergo further enzymatic transformations to become excreted in the urine as *N*-acetylcysteine adducts [(*N*-acetyl-*S*-(2-carboxamidoethyl) cysteine) (AAMA) and 2 isomeric GA *N*-acetylcysteine adducts, *N*-acetyl-*S*-(3-amino-2-hydroxy-3-oxopropyl)-l-cysteine] and *N*-acetyl-*S*-(1-carbamoyl-2-hydroxyethyl)-l-cysteine (GAMA and iso-GAMA). These mercapturic acids (MAs) serve as established short-term exposure biomarkers, because they are excreted within about 3 days following exposure and can easily be monitored noninvasively in spot and/or total urine samples. In humans, AAMA can be partially sulfoxidised to AAMA-sulfoxide (Fennell and Friedman 2005; Kopp and Dekant 2009).

The proportion of the presumably genotoxic CYP450 metabolite GA, escaping the detoxification reactions described above, is available to potentially damage DNA. The DNA *N*^7^-guanine adduct, *N*^7^-(2 carbamoyl-2-hydroxy ethyl)guanine (*N*^7^-GA-Gua), is by far the most abundant DNA adduct resulting from covalent DNA interaction upon exposure to AA (Eisenbrand 2020). It is known to be of rather low promutagenic activity and relatively unstable, being cleaved off by spontaneous or repair-mediated depurination, giving rise to apurinic sites. In vitro under conditions of neutral thermal depurination (37 °C) a half-life of 42 h for cleavage of the *N*^7^-GA-Gu adduct from calf thymus DNA has been reported (Gamboa da Costa et al. 2003). In vivo, after an AA dose of 18 mg/kg bw, *N*^7^-GA-Gua was found to be removed from DNA of rat liver, testes and brain at half-lives in the range of 50–80 h (Maniere et al. 2005). Metabolism and excretion of *N*^7^-GA-Gua is not extensively investigated but the adduct is conceived to be excreted to some extent in the urine. Accordingly, it has been identified in human urine after occupational exposure (Huang et al. 2018).

In contrast to GA adducts, covalent DNA adducts of the parent molecule AA have not been detected in animal or human tissues. Humans are less proficient than rodents in activating AA metabolically to the genotoxic metabolite GA. In contrast, detoxifying biotransformation, especially coupling of AA and GA to GSH, is more efficient in humans than rodents (Berger et al. 2011; Fennell and Friedman 2005; Fuhr et al. 2006).

AA has been classified as a genotoxic carcinogen (EFSA 2015a). In assessing human health risk, induction of neoplasms in multiple organs of rodents has been selected as the critical key outcome to derive a margin of exposure (MOE). As point of departure, a benchmark dose lower confidence limit for 10% extra tumour incidence (BMDL10) of 0.17 mg/kg bw/day was deduced from the incidence of Harderian gland adenomas and adenocarcinomas observed in an NTP study in male B6C3F1 mice orally exposed to AA for 2 years (EFSA 2015a). MOEs, calculated through dividing the BMDL10 by exposure estimates (mean and 95th percentile), were found in a range considerably below 500. According to EFSA, this indicates a concern with respect to human health risk (EFSA 2015a). Hence, there is continuing endeavour to minimise exposure, ideally to achieve a target MOE of > 10,000 considered of low public health relevance.

#### Existing knowledge on exogenous sources

Chronic dietary exposure of adults has been estimated to be on average between 0.4 and 0.9 μg/kg bw/day (95th percentile: 0.6–2.0 μg/kg bw/day), of children between 0.5 and 1.9 μg/kg bw/day (95th percentile: 1.4–3.4 μg/kg bw/day). AA is mainly found in carbohydrate-rich, heat-processed foods, such as, for example, French fries, potato chips/crisps, certain bread varieties and roasted coffee (EFSA 2015a).

##### Exogenous exposure from food

In its 2015 opinion, EFSA presented a large database on the occurrence of AA in foods. A total of 43,419 analytical results from food commodities collected and analysed since 2010 and reported by 24 European Countries and 6 food Associations was evaluated, giving overall consistent and complementary information (EFSA 2015a).

AA was found at the highest levels in solid coffee substitutes and coffee, and in potato fried products. For coffee, the CONTAM panel expressed its expectation that “due to dilution effects, lower levels are expected in ‘Coffee beverages’ and ‘Coffee substitutes beverage’ as consumed by the population”. This expectation was later confirmed, for example within carefully controlled human intervention studies based on AA dosimetry by duplicate diet methodology. Ingestion of four cups of coffee (together 500 mL during the day), freshly brewed from coffee pads containing 241 ± 11 µg/kg AA, resulted in an AA intake of 0.15–0.17 µg/kg bw by the volunteers. The associated urinary excretion of AA-related mercapturic acids was detected as a slightly enhanced yet still significant increase in AAMA (*p* < 0.01) and GAMA (*p* < 0.05) excretion. This short-term biomarker response remained, however, within the background range of 0.14 ± 0.10 μmol AAMA daily excreted by the volunteers on washout. Thus, coffee consumption at moderate intake level appeared to contribute to a minor extent to dietary AA exposure. In comparison, a single oral administration of stable isotope-labelled ^13^C_3_D_3_-AA (1 μg/kg bw) in water resulted in a prominent urinary biomarker signal (Goempel et al. 2017).

According to EFSA (2015a, b), the main contributor to total dietary exposure was generally the category ‘Potato fried products (except potato crisps and snacks)’. It was also noted that preferences in home-cooking can have a substantial impact on human dietary AA exposure. Although at that point in time a reliable Europe-wide temporal trend analysis was not feasible, a dataset of manufacturers measurements of AA levels in 40,455 samples of fresh sliced potato crisps from 20 European countries for the years 2002–2011 showed a substantial downward trend for mean levels of AA, from 763 ± 91.1 μg/kg in 2002 to 358 ± 2.5 μg/kg in 2011. For other food categories, a similar downward trend was not observed at that time.

Mitigation measures have been issued together with benchmark levels to reduce AA contents in food in the Commission Regulation (EU) 2017/2158 of 20 November 2017 (European Commission 2017). Further options to reduce the presence of AA in foods are available from the so-called Acrylamide Toolbox for precautionary measures to mitigate AA formation (FoodDrinkEurope 2019).

##### Exogenous exposure from other sources

Levels of AA in mainstream cigarette smoke have been reported to range from 1100 to 2340 ng/cigarette (Smith et al. 2000) or from 497.1 to 4168.8 ng/cigarette (Moldoveanu and Gerardi 2011). The CONTAM Panel concluded that tobacco smoking represents a more prominent source of AA exposure than the diet.

In contrast, non-dietary exposure to AA for the non-smoking general population was thought to be low (EFSA 2015a) and to potentially result from the ingestion of water treated with polyacrylamides containing residual AA as flocculants. A parametric value for AA in drinking water has been established at 0.10 μg/L (Council of the European Union 1998).

AA is listed as a prohibited substance in cosmetic products (European Commission 2009). For polyacrylamides used in cosmetic products, Annex III of the same regulation lists a maximal residual AA content of 0.1 mg/kg for leave-on products and 0.5 mg/kg for other cosmetic products.

In consideration of the above regulatory situation, exposure through drinking water and cosmetics is considered barely relevant when compared to exposure through food consumption.

Since AA is produced for a wide variety of industrial applications and is used in laboratories in the preparation of polyacrylamide gels for electrophoresis, there may be potential relevance concerning workplace-associated exposure. Overall, workers exposed at various working places to enhanced levels of AA have shown increased risk of mostly peripheral neurotoxicity, but no indication for an enhanced occupational cancer risk (EFSA 2015a).

#### Existing knowledge on endogenous sources

An unexpected discovery was the observation of an apparent endogenous formation of AA. This was first observed in rats in the course of carefully controlled feeding/oral gavage studies. Quantitative comparison of AA intake in feed with the urinary output of AA- and GA-related mercapturic acids, well established exposure biomarkers, revealed that in control animals the urinary output substantially exceeded the AA input (Watzek et al. 2012a).

This finding was subsequently confirmed in human studies using duplicate diet methodology. Detailed monitoring of exposure biomarkers in carefully controlled human intervention studies provided compelling evidence that AA is continuously biosynthesised in the human body. Based on human exposure biomarker dosimetry, this endogenous background exposure has been estimated to contribute about 0.2–0.4 μg AA/kg bw/day to overall exposure, indicating a similar level as the average exogenous consumer exposure associated with food intake. Thus, in addition to exogenous exposure to AA as a PRC in foods, sustained endogenous exposure to AA clearly must be considered (Goempel et al. 2017; Goerke et al. 2019; Ruenz et al. 2016; Watzek et al. 2012b).

The origin of the endogenous AA background is not fully clear at present. It is conceivable that AA may arise during the manifold biochemical reactions occurring during physiological energy metabolism of dietary substrates in the human organism. Reaction pathways encountered within the Maillard chemistry may supposedly play a role, potentially related to processes involved in the formation of glycation compounds (see “Glycation compounds (“advanced glycation end products”)”).

However, alternative formation pathways may prevail. AA may also be generated from acrolein (AC), either by addition of ammonia equivalents or by related biochemistry. AC is endogenously generated as well in humans, at considerably higher level than AA (see “Acrolein”). In addition to these pathways associated with intermediate metabolism in the mammalian host, AC and/or its analogues may play a prominent role as microbially generated AA precursors. It appears that glycerol acts as the major molecular precursor for AC in the human gut (Engels et al. 2016; Mu et al. 2018; Zhang et al. 2018). The microbial glycerol metabolite 3-hydroxypropanal is conceived to equilibrate with AC in the intestine and may easily react with ammonia or equivalents, such as amines and/or other nucleophiles, to generate AA and/or corresponding reaction products. A seminal observation pointing to this pathway came from the discovery of a reaction product of another PRC, the heterocyclic amine 2-amino-1-methyl-6-phenylimidazo[4,5-*b*]pyridine (PhIP), with acrolein to yield 7-hydroxy-5-methyl-3-phenyl-6,7,8,9-tetrahydropyrido[3′,2′:4,5]imidazo[1,2-*a*]pyrimidin-5-ium chloride (PhIP-M1) in urine and faeces of volunteers after consuming well done chicken. Formation of this PhIP-metabolite was ascribed to a bacterial transformation process mediated by strains isolated from human faecal samples (*Enterococcus* species, *Lactobacillus reuteri*), able to convert faecal glycerol into 3-hydroxypropanal (3-HPA) (Vanhaecke et al. 2006). For further information to formation of AC and human exposure, see “Acrolein”.

A recent human study on levels of *N*^7^-GA-Gua in DNA isolated from 56 healthy volunteers reported the in vivo presence of *N*^7^-GA-Gua in human DNA isolated from peripheral blood mononuclear cells (PBMC). In the majority of PBMC DNA samples, the levels found were above one adduct/10^8^ nucleosides. They were not found correlated to dietary habits or to blood glucose levels or Hb HbA1c. Instead, they were significantly correlated with the body mass index (BMI), showing continuous increase over three BMI classes (Hemgesberg et al. 2021a). Although the precise origin of this background of *N*^7^-GA-Gua DNA adducts in human PBMC at present is unclear, the observation of a correlation with the BMI rather than with certain dietary or lifestyle factors lends further support to the existence of an endogenous AA background in humans. Whether putatively enhanced AA formation, e.g. in the gut of individuals with enhanced BMI, may act on *N*^7^-GA-Gua levels in PBMC DNA and whether the gut microbiome is playing a role needs to be further investigated. It may be speculated that endogenous generation of AA, e.g. in the gut, may be accompanied by extrahepatic CYP dependent activation to GA. These background findings were in line with findings of a subsequent study reporting background level of 5–10 *N*^7^-GA-Gua adducts/10^8^ nucleosides in primary hepatocytes from AA-unexposed rats (Hemgesberg et al. 2021b). It was also reported that incubation with AA up to concentrations of 500 μM did not induce significantly increased DNA adduct formation above the background (after 24 h), which became measurable only at mM concentrations. This hypo-linear dose–response, presumably reflecting the existence of a threshold concentration, compares well with earlier in vivo results (see below) likewise reporting a hypo-linear dose–response of *N*^7^-GA-Gua DNA adduct formation in AA-treated rats, with a clearly dose-related increase in *N*^7^-GA-Gua adducts over background beyond a threshold dose level (Watzek et al. 2012a). For in vitro adduct formation in AA exposed rat hepatocytes, a composite lower bound of the 95% confidence interval of the benchmark concentration was calculated, leading to a 10% increase of *N*^7^-GA-Gua levels over background (BMC10) of 6.35 μM AA. Up to this value, an increase in *N*^7^-GA-Gua of more than 10% over the background seen in untreated hepatocytes may not be expected (Hemgesberg et al. 2021b).

#### Available biomarkers

Relevant biotransformation pathways of AA in mammalian systems (Fig. 5) also show the terminal reaction products primarily exploited for biomarker dosimetry. To assess exposure to AA, short-term and/or long-term exposure biomarkers may be monitored. In general, urinary MAs do not accumulate and their rapid clearance and complete urinary excretion within about 3 days makes them useful short-term biomarkers. Their monitoring allows to follow short-term variations in (dietary) AA exposure. They are also ideally suited to study excretion kinetics using stable AA isotopologues, such as, for instance, ^13^C_3_D_3_-AA, and are also useful to elucidate endogenous background exposure levels (Goempel et al. 2017).

In contrast to MAs, the adducts of AA and GA to the N-terminal valine of haemoglobin (Hb) have widely been used as long-term exposure biomarkers in animal experiments and in human studies. Hb adducts are not repaired and accumulate over the lifespan of Hb in erythrocytes, which is about 120 days in humans. Hb adduct dosimetry thus represents a measure of chronic exposure to AA.

Of note, simultaneous monitoring of MAs and Hb adducts allows to obtain comprehensive information about time related exposure, individual biotransformation and the ratio between detoxifying and toxifying biotransformations. This may provide important mechanistic insights, for instance by means of carefully controlled animal experiments or human nutritional intervention studies.

In rats ingesting experimental lab chow containing 100 µg/kg AA for 9 days, formation of AA Hb adducts depicted linear dose–response behaviour, showing treatment-associated cumulative build up in erythrocytes. In contrast, GA Hb adducts were not found significantly enhanced above untreated control. However, urinary excretion of the short time exposure biomarker GAMA proved that GA was formed in the liver by CYP 2E1 oxidation, yet practically quantitatively coupled to GSH. This allowed to conclude that at this dose level, the GA arising from metabolic oxidation of AA in the liver is effectively detoxified (Berger et al. 2011). This conclusion finds support by the observation that in primary rat hepatocytes, GSH coupling of AA is faster than its CYP 2E1 mediated oxidation to GA (Watzek et al. 2013).

#### Impact of endogenous formation on risk assessment

There is compelling evidence for a sustained background of endogenous exposure to AA, primarily unrelated to the exogenous exposure. Whereas the latter can be diminished by appropriate risk management measures, endogenous background exposure appears much less prone to targeted reduction. It should, therefore, be appropriately taken into consideration when performing human health risk assessment.

Of note, the variability of the endogenous AA exposure may depend on yet largely unknown factors of influence that appear important to explore to reliably inform about the variance of endogenous exposure on a population level.

With such data, knowledge-based refinement of human health risk assessment may be achieved. The ultimate aims of such future research are:

1. To approach a better-informed holistic risk assessment.
2. To establish the endogenous exposure level as an accepted reference for risk assessment, potentially serving as point of departure against which to evaluate the cognate exogenous exposure.

### Acrolein

#### Characterisation, formation, occurrence and public health concern

The 2-alkenal acrolein (2-propenal) is a prototypic α,β-unsaturated carbonyl compound, avidly reacting with a wide spectrum of nucleophiles by covalent addition across the carbonyl-activated α,β-double bond, called the Michael reaction. The high electrophilic reactivity of acrolein (AC) and its α,β-unsaturated alkenal analogues is responsible for the well-known biological effects of these alkenals. AC is known to be generated by (incomplete) combustion of fuels, wood or plastics.

In food, heat-induced formation of AC from various food constituents such as fats, amino acids and carbohydrates is well established (Shibamoto 2009; Stevens and Maier 2008). Thermal decomposition of precursor molecules, including glycerol and glycerides and amino acids such as methionine and threonine, can lead to the formation of AC as well as heating of carbohydrate-rich foods. AC has been detected in processed foods, in products containing heated animal fats and plant oils and in the volatile fraction (head space) of certain foods such as fish, bread, poultry and beef (WHO 2002). Heat-induced formation of AC in fats/oils depends on the fatty acid composition, the heating time and the temperature. Thus, whereas in oils and fats not subject to heating processes during/after refining, only traces of AC (up to 20 µg/kg) were reported, deep-frying fats showed markedly increased AC contents, depending on usage length (0.2–1.4 mg/kg = ppm) (Ghilarducci and Tjeerdema 1995; Kuballa et al. 2012; Schuh 1992).

It may be presumed that heat-treated foods, depending on the type of thermal treatment, may contain significant amounts of AC. In addition, certain microorganisms such as heterofermentative lactobacilli and enterobacteriaceae can enzymatically generate 3-HPA which functions as a direct precursor of AC by water elimination, for instance during distillation of alcohol from fermentation mashes (Bauer-Christoph 2010). Other alcoholic beverages, e.g. red wine and lager beer, may also contain AC, mostly in low concentrations (Ewert et al. 2011; Feron et al. 1991; Kuballa et al. 2012; Saison et al. 2009). Microbial formation of AC has been reported to also occur in the human gastrointestinal tract, where the predominant precursor is supposed to be glycerol (see “Existing knowledge on endogenous sources”).

Inhalative AC exposure of rats caused irritation and inflammation of exposed mucosae and necrosis of the pulmonary tissue, as reported by the U.S. Environmental Protection Agency (US-EPA 2003). Similarly, oral exposure resulted in inflammation and necrosis of the forestomach of rats. Reversible GSH depletion was also observed (Arumugam et al. 1999; Witz 1989). Rodent acute and subchronic oral toxicity studies on AC were summarised in an opinion from the DFG Senate Commission on Food Safety (SKLM) (SKLM 2013). AC induced mainly local effects, with hyperplastic lesions in the forestomach observed at dosages of ≥ 2.5 mg/kg bw appearing as the most sensitive parameter. In 90-day subchronic oral toxicity NTP studies, no-observed-adverse-effect-level (NOAEL) values of 1.25 mg/kg bw/day for female mice and male rats and of 0.75 mg/kg bw/day for female rats were deduced. In male mice, an increase of the number of epithelial hyperplastic lesions already occurred at the lowest dose tested (1.25 mg/kg bw/day), so that a dose without effect could not be determined. From the rat study, based on a NOAEL value of 0.75 mg/kg bw/day, a tolerable daily intake (TDI) of 7.5 μg/kg bw/day was deduced by applying a safety factor of 100 (Abraham et al. 2011a; WHO 2002).

AC can form DNA adducts (in vitro and in vivo), and reactions primarily with guanine residues have been described (Chung et al. 1984; Nath and Chung 1994; Zhang et al. 2007). The main DNA adduct formed in vitro is *γ*-hydroxy-propanodeoxyguanosine (*γ*-OH-PdG), a minor product *α*-hydroxy-propanodeoxyguanosine (*α*-OH-PdG). Of note, AC-DNA adducts were also detected in untreated rats and mice as well as in human samples (blood, liver, mammary gland) (Nath and Chung 1994). In human samples, the *N*^7^-adduct 7-(2′-carboxyethyl)guanine was present in substantially higher concentrations than the cyclic adducts. This adduct has been speculated to result from binding of acrylic acid generated from AC to the *N*^7^ of guanine in DNA. Genotoxic/mutagenic effects of AC have been reported at non-cytotoxic concentrations in bacterial test systems (US-EPA 2003). Data were not conclusive since depending on the strain and test conditions positive as well as negative results were observed (Irwin 2006; MAK-Commission 2012a).

In mammalian cell test systems, results were also inconclusive, primarily because of the potent cytotoxicity of AC. Hypoxanthine–guanine phosphoribosyltransferase mutations have been observed sporadically, e.g. in V79 cells, AC was found to be mutagenic in the mM concentration range but was later reported not to induce a significant mutagenic response in the presence or absence of metabolic activation (Irwin 2006; MAK-Commission 2012a; Parent et al. 1991; SKLM 2013; Smith et al. 1990). In HepG2 cells, AC induced DNA strand breaks (at 12.5 and 25 μM) and DNA–protein crosslinks (at 50–100 μM) (Li et al. 2008). AC was not found to be mutagenic in human and murine fibroblasts (Kim et al. 2007). Limited data obtained from in vivo studies in mice, rats, and dogs regarding potential genotoxic/mutagenic effects of AC altogether were deemed negative so far (SKLM 2013).

Different bodies/commissions have classified the carcinogenic potential of AC:

- IARC classified AC into Category 3 (not classifiable as to its carcinogenicity to humans, based on inadequate evidence in humans and in experimental animals for the carcinogenicity of acrolein) (IARC 1995). In a recent evaluation, AC is classified as “possibly carcinogenic to humans” (Group 2A) on the basis of “sufficient” evidence of carcinogenicity in experimental animals and “strong” mechanistic evidence (IARC 2020).
- MAK Commission (Commission for the Investigation of Health Hazards of Chemical Compounds in the Work Area) of the DFG: Category 3B (Substances that cause concern that they could be carcinogenic for man but cannot be assessed conclusively because of lacking data) (MAK-Commission 2012a; SKLM 2013).
- US-EPA: “data are inadequate for an assessment of human carcinogenic potential by either the inhalation or oral routes of exposure” (US-EPA 2003).

#### Existing knowledge on exogenous sources

##### Exogenous exposure from food

Of note, adequate data on AC contents in heated foods are largely missing. This appears to result from a lack of reliable analytical methods for AC determination in various food matrices. For a few food groups that lend themselves to specific determination of AC contents in the volatile fraction, head space analysis has been used as the method of choice using GC or GC/MS (Ewert et al. 2011). It was reported that acrolein in hydroalcoholic solution rapidly reacts with water to form 1,3,3-propanetriol and 3-hydroxypropionaldehyde, a degradation that was found to be preventable by the stabiliser hydroquinone (Kachele et al. 2014). Because of the high Michael reactivity of AC, covalent binding to food constituents and/or (reversible) formation of non-volatile adducts with food constituents other than water may presumably contribute to limit its availability to headspace GC. Yet, such reversible adducts may still, at least in part, liberate AC in the gastrointestinal tract. In conclusion, the limited database regarding acrolein levels in foods does not allow a reliable assessment of the AC exposure through foods.

Based on the assumption that all foods contain maximal reported levels of AC, a theoretical maximal exposure of around 1 mg/person/day (17 μg/kg bw/day) has been estimated (SKLM 2013). The environmental AC exposure of humans mainly occurs through inhalation. A more reliable exposure estimate may be possible by making use of exposure biomarkers, e.g. through the quantitation of AC-associated mercapturic acids such as 3-hydroxypropylmercapturic acid (3-HPMA) and 2-carboxyethylmercapturic acid (CEMA) in urine. On the assumption that about 20% of a given AC intake is excreted as 3-HPMA, a total exposure of about 300–1400 μg/day or 5–24 μg/kg bw/day has been estimated (SKLM 2013). Stringently controlled human biomarker studies arrived at similar estimates of about 10–12 μg/kg bw/day, based on monitoring of urinary 3-HPMA, supposedly excreted at 20% of the total AC dose (Ruenz et al. 2019).

##### Exogenous exposure from other sources

The US-EPA has estimated an average atmospheric AC concentration of about 14.3 μg/m^3^ (Stevens and Maier 2008; US-EPA 2003). However, persistence and distribution in the environment is considered rather low, due to the high reactivity of AC (WHO 2002).

Based on an average AC concentration in the atmosphere of about 14.3 μg/m^3^ (6.2 ppb) and a mean respiratory volume in humans of about 20 m^3^/day (ECHA 2010), an AC exposure via the atmosphere of 286 μg/day can be estimated. In the case of smokers (about 20 cigarettes/day), an additional exposure (50–100 μg/cigarette) of up to 2 mg, corresponding to 0.03 μg/kg bw/day, has to be taken into account.

Workplace-associated inhalative AC exposure can occur at certain workplaces, e.g. in (commercial) kitchens, in which edible oil is heated up to temperatures above 180 °C (roasting/deep-frying). Depending on the conditions, appreciable amounts, up to 250 mg AC/kg of used oil may reportedly be released into ambient air (Fullana et al. 2004; Li et al. 2005; Umano and Shibamoto 1987). Up to 0.55 mg AC/m^3^ air were detected in ambient air of kitchens on heating deep-frying fat (Ho et al. 2006; Schuh 1992). Cigarette smoke also contributes to the inhalative uptake of AC. In main stream smoke of cigarettes, 56–118 μg /cigarette were reported. AC formation was found to depend on glycerol and sugar contents in tobacco (Carmines and Gaworski 2005; Talhout et al. 2006). An estimate of aggregate AC exposure was attempted by referring to a median 3-HPMA concentration measured in spot urine samples of non-smokers and assuming about 50% of oral AC being excreted as 3-HPMA (which appears too high as excretion estimate). A putative AC exposure of 2.1–2.4 μg/kg bw/day resulted, based on a daily urine volume of 1.5 L. The same calculation based on maximal instead of median values resulted in an exposure estimate of about 30 μg/kg bw/day (Abraham et al. 2011a).

#### Existing knowledge on endogenous sources

AC may be formed endogenously from carbohydrates, triacylglycerides, and certain amino acids. Non-microbial endogenous formation routes of AC include myeloperoxidase catalysed formation of AC from threonine, amine oxidase catalysed formation from spermine and spermidine, and formation as a by-product of lipid peroxidation (Ruenz et al. 2019).

In addition to these pathways associated with intermediate metabolism in the mammalian host, AC and/or its analogues may play a prominent role as microbially generated AA precursors. It is well established that AC can be generated by a wide spectrum of microbiota, including the genera *Klebsiella*, *Enterobacter*, *Citrobacter*, *Clostridium*, *Lactobacillus*, and other prominent constituents of the human intestinal microbiome (Engels et al. 2016). It appears that glycerol acts as the major molecular precursor for AC in the human gut (Engels et al. 2016; Mu et al. 2018; Zhang et al. 2018). Glycerol is present in the intestine (1) as nutritional component, (2) as a product of digestion of luminal and membrane lipids and (3) as consistently generated product of microbial fermentation. The above-mentioned gut microbiota can metabolise glycerol into 3-HPA, a microbial biotransformation catalysed by vitamin B12-dependent glycerol/diol dehydratases (Sauvageot et al. 2000; Talarico and Dobrogosz 1990; Vollenweider and Lacroix 2004). 3-HPA easily eliminates water to yield the dehydration product AC. AC and its hydrate 3-HPA equilibrate in aqueous media and form part of a multicomponent system called Reuterin, after *Lactobacillus reuteri* (Engels et al. 2016).

#### Available biomarkers

After absorption, AC is rapidly conjugated to GSH, mainly in the liver, and converted into the final metabolites, 3-HPMA and CEMA which are excreted in the urine. The major AC-derived metabolite in urine is the reductive metabolite *N*-acetyl-*S*-(3-hydroxypropyl)-l-cysteine (HPMA). After oral administration of ^14^C-labelled AC to rats, about 60% of the administered radioactivity was excreted via the urine, about 20% of the administered total dose was excreted as 3-HPMA in rats. AC exposure has been assessed based on a daily HPMA excretion (200–1000 μg/24 h) in urine of non-smokers or former smokers on abstinence and assuming 20% urinary excretion of ingested AC, in analogy to data obtained from rats. As mentioned above, this resulted in a total AC exposure estimate of 300–1400 μg/day, suggesting an intake of 5–24 μg/kg bw/day (Ruenz et al. 2019).

#### Impact of endogenous formation on risk assessment

A TDI of 7.5 µg/kg bw/day of acrolein was derived by a working group of the WHO in 2002 (WHO 2002). This TDI was set without taking into account the sustained endogenous AC background exposure. Evidence from well-controlled human biomarker studies indicates a level of total AC exposure of about 10–12 μg/kg bw/day, based on monitoring of urinary HPMA (Ruenz et al. 2019). A total exposure of about 300–1400 μg/day or 5–24 μg/kg bw/day has been estimated (SKLM 2013). Consequences for risk assessment are not yet clear but there is some evidence suggestive of the potential relevance of (endogenous) AC exposure to human health. For example, a human biomarker-based epidemiologic study provided evidence for a dose–response-related positive association of diabetes and insulin resistance with AC exposure, as measured by urinary AC related mercapturic acid excretion (Feroe et al. 2016). In another human study, levels of acrolein-conjugated protein in the plasma and CSF of Alzheimer disease (AD) patients were found to be significantly higher than those of control subjects, whereas the levels of urinary acrolein-related mercapturic acids were markedly decreased. These data suggest that deregulated detoxifying AC metabolism may be correlated with neuronal damage in AD patients (Tsou et al. 2018).

Taken together, the total AC exposure as monitored by biomarker dosimetry, shows negligible influence of different diets, and clearly exceeds the TDI for exogenous AC as derived by the WHO (2002). It is about 30-fold higher than dietary exposure to the closely related PRC of public concern, AA (Ruenz et al. 2019). The data strongly point to endogenous AC generation by pathways of mammalian and/or microbial metabolism. The biological consequences of such sustained AC exposure are far from being established and require further detailed research, The same applies to detailed elucidation of pathways of endogenous, (supposedly mainly microbial) AC generation and relevant precursors. Future epidemiology may need to take into account this high-sustained endogenous AC background when aiming to derive associations between a given exogenous PRC exposure and human health effects.

### α,β-Unsaturated alkenals

#### Characterisation, formation, occurrence and public health concern

**Fig. 6:**
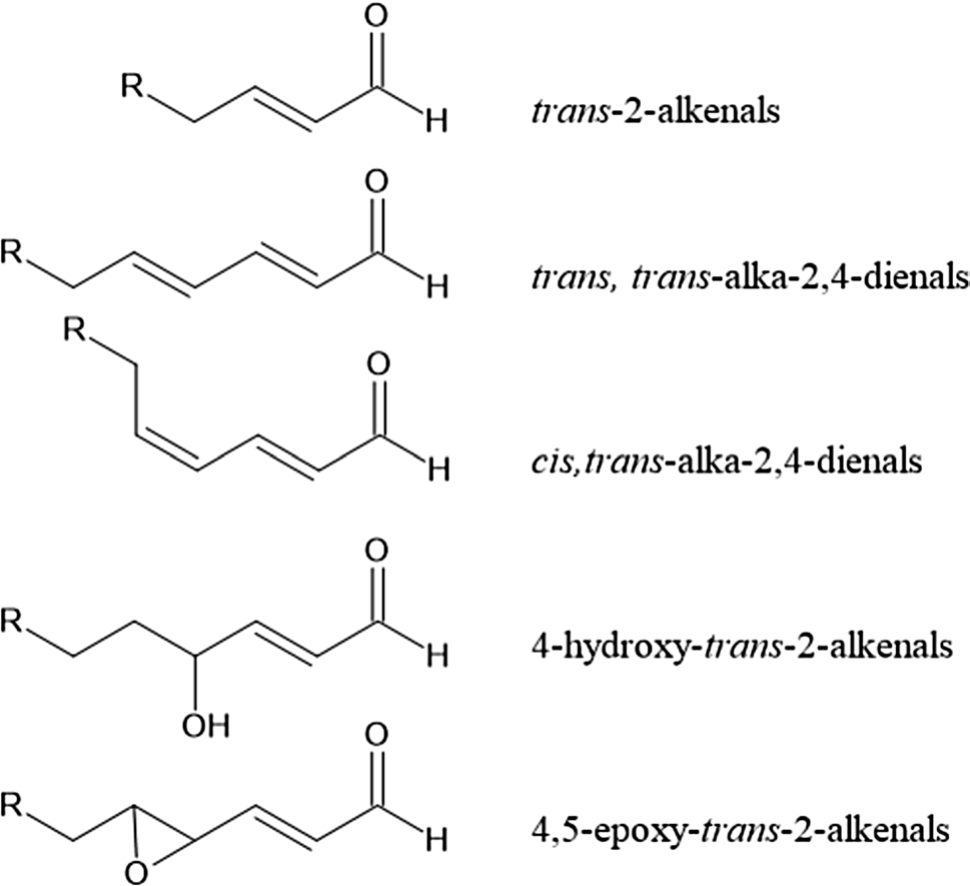
Structural formulas of the different type of α,β-unsaturated aldehydes detected in the study reported by Grootveld et al. (2014) in thermally stressed oils and fats

In addition to acrolein, also other α,β-unsaturated alkenals may be formed upon heating of food, especially upon heating of oils and fats. Mainly based on ^1^H NMR analysis, the presence of various classes of aldehydes in heated oils and fats, including α,β-unsaturated alkenals such as *trans*-2-alkenals (also including acrolein), *trans,trans*-alka-2,4-dienals, *cis*,*trans*-alka-2,4-dienals, 4-hydroxy-*trans*-2-alkenals and 4,5-epoxy-*trans*-2-alkenals (Fig. 6) was reported (Grootveld et al. 2014). These alkenals represent lipid oxidation products generated in culinary oils especially upon heating.

Presence of those α,β-unsaturated alkenals is a concern because the α,β-unsaturated aldehyde moiety is considered a structural alert for genotoxicity. However, not all α,β-unsaturated aldehydes express their in vitro genotoxicity in vivo (SKLM 2002, 2013; Younes et al. 2018).

The SKLM concluded that 2-alkenals can cause cytotoxic and genotoxic effects, but are also rapidly detoxified by oxidation or reduction as well as by GSH conjugation, and that toxicity and genotoxicity will become apparent only at high doses, when such detoxification mechanisms become overloaded (SKLM 2002, 2013).

In 2018, EFSA published an opinion evaluating the Flavouring Group containing α,β-unsaturated aldehydes, including 2-hexenal and others, indicating that based on the available data concerns over genotoxicity could be excluded for all 74 substances in the group (Younes et al. 2018).

#### Existing knowledge on exogenous sources

##### Exogenous exposure from food

α,β-Unsaturated aldehydes (including acrolein) were detected in culinary oils at levels of 20 mmol/kg oil and in some cases 50 mmol/kg oil when heated to 180 °C for ≥ 30 min (Grootveld et al. 2014). The study also reported that consumption of a 300 g serving of French fries containing 35 g thermally stressed oil with 10 mmol aldehydes/kg would result in an intake of 0.35 mmol aldehydes. Taking into account that over half of the aldehydes will be α,β-unsaturated aldehydes this would amount to an estimated intake for a 70 kg person of 0.0025 mmol α,β-unsaturated aldehydes/kg bw (Grootveld et al. 2014).

It is also of interest to note that others reported substantially lower levels of alkenals in soybean oil after 6 h frying at 185 °C: 0.04 mg/kg oil for 2-hexenal, 1.36 mg/kg oil for 2-heptenal, and 2.10 mg/kg oil for 2-nonenal mg/kg oil giving a total level of 3.5 mg 2-alkanals/kg oil (Seppanen and Csallany 2001). Intake values upon consumption of 10 ml of this oil analysed by a 70 kg person would amount to a value of only 0.5 µg 2-alkanals/kg bw (Seppanen and Csallany 2001). Some of the *trans*-2-alkanals are also known to be naturally present in food or are used as food flavours. For example, *trans*-2-hexenal naturally occurs in bananas at levels amounting to 76 mg/kg bananas (Adams et al. 2008). For a 70 kg individual, eating 100 g of bananas would result in intake of *trans*-2-hexenal at 108 µg/kg bw. Kiwamoto et al. reported a physiologically based kinetic study on detoxification and DNA adduct formation of 18 acyclic foodborne α,β-unsaturated aldehydes. The study revealed that DNA adduct formation in the liver increases with decreasing bulkiness of the molecule especially due to less efficient detoxification (Kiwamoto et al. 2015). Acrolein was identified to induce the highest DNA adduct levels. At realistic dietary intake, the predicted DNA adduct levels for all aldehydes were two orders of magnitude lower than endogenous background levels observed in disease free human liver, suggesting that for all 18 aldehydes DNA adduct formation is negligible at the relevant levels of dietary intake.

In addition to *trans*-2-alkenals, also other α,β-unsaturated alkenals have been detected in heated oils. For example, it was reported levels of *trans,trans*-alka-2,4-dienals in heated oils that amounted to about 1.5 up to 37 mmol/kg oil depending on the number of thermal stressing periods at 180 °C and the type of oil (Grootveld et al. 2014). Others reported levels of 2,4-heptadienal and 2,4-decadienal in soybean oil after 6 h frying at 185 °C that amounted to 0.60 mg/kg oil and 8.56 mg/kg oil, respectively, which is again substantially lower than the levels of total *trans*,*trans*-alka-2,4-dienals reported by Grootveld et al. (Grootveld et al. 2014).

4-Hydroxy-*trans*-2-alkenals reported to be formed upon heating of oils and fats include 4-hydroxy-2-hexenal (HHE), 4-hydroxy-2-octenal (HOE) and 4-hydroxy-2-nonenal (HNE) (Seppanen and Csallany 2001, 2002). Levels of 4-hydroxy-*trans*-2-alkenals of about 2–8 mmol/kg oil were reported later (Grootveld et al. 2014). In another study, levels of HHE and HNE ranged from 64 to 429 µg/kg oil and from 80 to 499 µg/kg oil, respectively (Surh and Kwon 2005). Levels of 0.17 mg/kg oil for HHE, 0.54 mg/kg oil HOE, and 2.45 mg/kg oil for HNE were reported in soybean oil after 6 h frying at 185 °C (Seppanen and Csallany 2001, 2002).

##### Exogenous exposure from other sources

Exposure to cigarette smoke and other sources known to cause oxidative stress and lipid peroxidation can also be expected to contribute to the exposome of HNE (Kode et al. 2006) and potentially also of other α,β-unsaturated alkenals.

#### Existing knowledge on endogenous sources

Given that these α,β-unsaturated alkenals are formed in a process known as lipid peroxidation, it is likely that formation of (some of) these α,β-unsaturated alkenals may also occur endogenously. This has best been described for HNE which is a well-known product of endogenous (lipid) peroxidation (Esterbauer et al. 1991; Schaur et al. 2015; Spickett 2013). In addition, HNE can also be generated enzymatically by cyclooxygenase-2 and lipoxygenase (Wang et al. 2013; Zhang and Forman 2017).

A review presented an overview of plasma and tissue levels of HNE and the corresponding references (Zhang and Forman 2017). Concentrations in human plasma may amount to 0.28–0.68 μM, in rat hepatocytes they were in the range of 2.5–3.8 μM, and in human blood monocytes and other types of cells levels were reported to be higher than those in plasma. The same review also indicated that the levels in different tissues may vary due to tissue-dependent differences in HNE metabolising capacity. Under conditions of oxidative stress and diseases, HNE levels have been reported to be significantly increased in plasma and tissues [(Zhang and Forman 2017) and references therein].

Given that HNE is formed endogenously under conditions of oxidative stress, it can also be expected to be formed endogenously at elevated levels upon heavy physical exercise and/or upon smoking.

#### Available biomarkers

Deoxyguanosine adducts of HNE have been reported as endogenous DNA lesions in rodents and humans (Chung et al. 2000) and may thus be considered as biomarkers of exposure. In addition, levels of HNE-protein adducts as well as metabolites resulting from conjugation of HNE with GSH (i.e. HNE-GSH, 4-hydroxynonenal-3-yl-mercapturic acid and its reduced form 1,4-dihydroxynonenal mercapturic acid) may be used as biomarkers of exposure instead of free HNE concentrations (Zhang and Forman 2017). However, since HNE may be formed during conditions of oxidative stress, elevated levels of DNA and protein adducts of HNE as well as GSH-derived HNE metabolites may not necessarily reflect exogenous exposure but may also be due to endogenous formation resulting from oxidative stress (Mally et al. 2007; Volkel et al. 2005).

#### Impact of endogenous formation on risk assessment

In addition to potential food-derived sources of α,β-unsaturated alkenals, there are also several endogenous sources of α,β-unsaturated alkenals. These include their generation by normal physiological processes, but also their potentially enhanced endogenous formation under conditions of oxidative stress including smoking or heavy physical exercise. The extent to which endogenous sources may add to the total exposome, may differ with the α,β-unsaturated alkenal under consideration. For HNE, it is most obvious that endogenous exposure adds to the overall exposure and may have to be taken into account in risk assessment, potentially even in risk assessment of related α,β-unsaturated alkenals.

### Glycation compounds (“advanced glycation end products”)

#### Characterisation, formation, occurrence, and public health concern

**Fig. 7:**
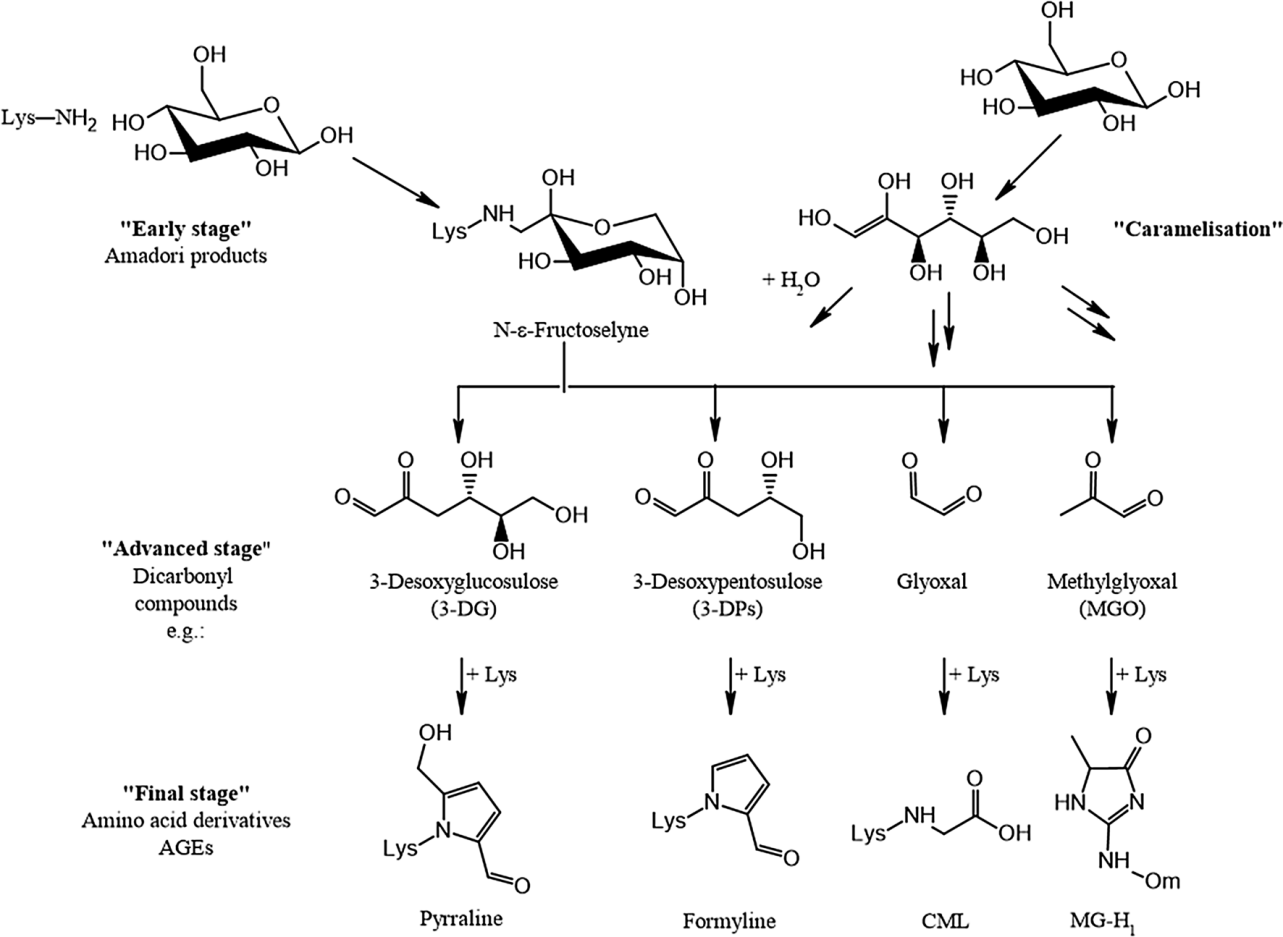
Selected glycation compounds formed during different stages of the Maillard reaction. (adapted from unpublished work, T. Henle, Institute of Food Chemistry, Technische Universität Dresden, Germany). *CML* *Nε*-(carboxylmethyl)-l-lysine, *MG-H1* *Nδ*-(5-hydro-5-methyl-4-imidazolon-2-yl)-l-ornithine

The term “glycation compounds” summarises a wide range of structurally diverse compounds that derive from the Maillard reaction, a network of chemical reactions between reducing sugars and amino acids that produces colour, aroma and flavour of processed food (Hodge 1953; Maillard 1912). It comprises Amadori compounds (e.g. *N*-*ε*-fructoselysine) that are formed in the early stage of the Maillard reaction from reaction of reducing sugars with free or peptide-bound amino acids, dicarbonyl compounds (e.g. glyoxal) formed by degradation of Amadori products during advanced stages of the Maillard reaction, glycated peptide-bound amino acid derivatives that result from reaction of dicarbonyl compounds with the ε-amino group of lysine or the guanidino group of arginine (e.g. carboxymethylysine), as well as stable end products (e.g. 5-hydroxymethylfurfural, AA) and probably a significant number of as yet unidentified compounds (Fig. 7) (Hellwig et al. 2019).

Following identification of glycated Hb in blood of patients suffering from diabetes (Kunkel and Wallenius 1955; Rahbar 1968), Monnier and Cerami (1981) first reported that the Maillard reaction also occurs in vivo and suggested that the pigmented protein adducts and protein crosslinks formed may play a role in ageing and cataractogenesis (Monnier and Cerami 1981). The term “advanced glycation end products” (AGEs), which is now frequently used as a summary term for glycation compounds, including those present in food, was initially introduced for these “brown fluorescent pigments which crosslink proteins” formed by the Maillard reaction in vivo.

Numerous studies have since established that AGEs formed endogenously (e.g. as a consequence of a high dietary sugar intake) accumulate in the body and may contribute to ageing and a range of diseases, including diabetic complications such as retinopathy, neuropathy and nephropathy, as well as cardiovascular disease, neurodegeneration, chronic inflammation and cancer via their ability to alter protein structure and function or to act as ligands for cellular receptors. Based on increasing evidence for adverse effects of AGEs produced by endogenous glycation reactions, the question of whether exposure to dietary glycation compounds may also pose a risk to human health has gained great attention. Dietary AGE Maillard reaction products are not only considered to reduce nutritional quality but have also been associated with food allergy, diabetes and metabolic syndrome, atherosclerosis and cardiovascular disease, ureamia and kidney disease, declined cognitive function, ageing and cancer (Cepas et al. 2020; Nowotny et al. 2018; Poulsen et al. 2013; Smith et al. 2017; Sowndhar Rajan et al. 2018; West et al. 2014). Even though structure–activity relationships between defined dietary glycation compounds and adverse health effects are lacking, this has led some to propose that an “AGE-rich diet” presents a nutritional risk. Considering the structural diversity of glycation compounds formed in food during heating, which includes highly reactive dicarbonyl compounds as well as stable peptide-bound glycated amino acid derivatives, the variability in composition depending on food matrix and heating conditions, and the fact that analysis of individual glycation compounds in food provides limited information on the overall mixture, it is evident that causal relationships between dietary “AGE” exposure and adverse health effects cannot be established based on an undefined dietary “AGE content” given as a sum parameter (Hellwig et al. 2019).

Considering their chemical reactivity and thus apparent potential to cause toxicity through covalent binding to cellular macromolecules, dicarbonyl compounds such as glyoxal, methylglyoxal and 3-deoxyglucosulose are among the most widely studied dietary glycation compounds. While there are limited data on the absorption and distribution of glyoxal in humans and experimental animals, it is well established that glyoxal is efficiently detoxified via the cytosolic GSH-dependent glyoxalase system as well as via 2-ketoaldehyde dehydrogenase. Glyoxal has been shown to cause irritation to the gastrointestinal tract and to produce degenerative changes in the kidney and pancreas as the main target organs. While there are no long-term oral carcinogenicity studies on glyoxal to establish that it is carcinogenic, there is clear evidence for genotoxicity. Glyoxal directly reacts with DNA bases to form stable adducts with guanosine by reaction with the *N*^1^ and the exocyclic nitrogen of guanine. Reaction of glyoxal with deoxycytidine yields 5-hydroxyacetyl-deoxycytidine, which may be deaminated to form deoxyuridine. Glyoxal also forms crosslinks between deoxyguanosine and deoxycytidine or deoxyadenine (Kasai et al. 1998). Glyoxal is mutagenic in several strains of *Salmonella typhimurium* and has been shown to induce single-strand breaks in the pyloric mucosa and liver of rats, DNA repair, chromosomal aberrations, and sister chromatid exchanges (Madhaven 2000; MAK-Commission 2014; Vilanova et al. 2017; WHO 2004). Similarly, methylglyoxal is mutagenic in the absence of exogenous metabolic activation in *Salmonella typhimurium*, *E. coli* and *Saccharomyces cerevisiae* (IARC 1991). Methylglyoxal also induces sister chromatid exchange, chromosomal aberrations and micronuclei in mammalian cells (IARC 1991; Marnett 1994). It has been shown to form DNA adducts with adenine and guanine bases in vitro to give rise to *N*^2^-(1-carboxyethyl)-2′-deoxyguanosine (CEdG) and *N*^6^-(1-carboxyethyl)-2′-deoxyadenosine (CEdA) as well as DNA crosslinks (Frischmann et al. 2005; Marnett 1994). There is also evidence that covalent binding of methylglyoxal to cellular proteins, i.e. glycation of proteins, such as histones and glyceraldehyde-3-phosphate dehydrogenase (Lee et al. 2005) may alter chromatin structure (Galligan et al. 2018) and inhibit mitochondrial respiration and glycolysis, respectively, and may thus link protein adduction by methylglyoxal to altered cell function and metabolism. There are, however, no adequate in vivo studies on the chronic toxicity and carcinogenicity of methylglyoxal (IARC 1991). Similarly, there are only limited data on 3-deoxyglucosone in vivo, which so far suggest a low rate of absorption from the GI tract and rapid excretion in urine, predominantly as 3-deoxyfructose (Hayase and Kaneko 1998; Kato et al. 1990). 3-Deoxyglucosone readily reacts with deoxyguanosine (Hayase and Kaneko 1998); it is mutagenic and induces chromosomal aberrations in vitro (Hayase and Kaneko 1998; Nishi et al. 1989).

In a risk characterisation based on a hypothetical worst-case dietary exposure for the general population conducted by WHO (2004), a hypothesised worst-case exposure of 10 mg/day, corresponding to 0.16 mg glyoxal/kg bw, was calculated based on concentrations of glyoxal in food. This worst-case exposure estimate was considered to be in the range of a tolerable intake of about 0.2 mg/kg bw for lifetime oral exposure derived from repeated dose oral toxicity studies on glyoxal in animals, which consistently revealed NOAEL values of around 100 mg/kg bw/day, and use of a composite uncertainty factor of 500 to account for interspecies and interindividual differences (100) as well as less-than-lifetime exposure. Thus, dietary exposure to glyoxal would not be considered a health risk, although it should be noted that carcinogenic potential was not considered due to the lack of data.

From a toxicological perspective, reaction of dicarbonyl compounds with free or protein-bound amino acids in food may be seen primarily as a mechanism of detoxication of these reactive intermediates. Whether or not food-derived free or protein/peptide-bound glycated amino acid derivatives themselves pose a health risk remains to be established. There are very limited data on the absorption and toxicity of free or protein-bound glycated amino acid derivatives other than carboxymethyllysine (CML) and *N*-*ε*-fructoselysine (FL). Absorption of glycated amino acid derivatives from the GI tract may depend on the free or protein-bound form and on the matrix. Studies by Hellwig et al. (2014) suggest that CML and FL may be released during simulated gastrointestinal digestion of glycated proteins (Hellwig et al. 2014). In support of this, ^13^C-*N*-*ɛ*-carboxymethyllysine was detected in various tissues of mice following dietary exposure to protein-bound ^13^C-labelled *N*-*ɛ*-carboxymethyllysine, although it was not possible for the authors to distinguish between protein-bound and free CML in tissues (Tessier et al. 2016). Such distinction may be important as there is evidence that protein-bound CML may be a ligand to the AGE receptor (RAGE) and subsequently elicit an inflammatory response, whereas free CML appears not to bind to RAGE. RAGE, initially identified and named based on its ability to bind AGEs, is a multiligand transmembrane receptor of the immunoglobulin superfamily that is expressed in a wide range of cell types and acts as a pattern recognition receptor (Poulsen et al. 2013). Binding of a ligand to RAGE induces oxidative stress and an inflammatory response through NF-κB (nuclear factor ‘kappa-light-chain-enhancer’ of activated B cells) pathway activation and subsequent production of pro-inflammatory cytokines (Poulsen et al. 2013). Oral toxicity studies using free CML demonstrate low acute toxicity (LD_50_ mice > 5000 mg/kg bw) but suggest that repeated administration of free CML may impair liver and kidney function (Li et al. 2015; Liu et al. 2016). The molecular mechanisms underlying these effects have not been studied so far.

The caramelisation reaction also gives rise to a range of stable end products. An example is 5-hydroxymethylfurfural (5-HMF) present in a wide variety of food items. Although 5-HMF can be bioactivated to a reactive and mutagenic metabolite 5-sulphoxymethylfurfural, the currently available data suggest that 5-HMF is not genotoxic and does not induce neoplastic changes in the intestinal tract (Abraham et al. 2011b). Based on limited evidence of 5-HMF carcinogenic potential and overall low toxicity of 5-HMF, the German Federal Institute for Risk Assessment (BfR) considered that the margin of safety of > 100 calculated based on maximum estimated exposure of 5-HMF < 500 µg/kg bw (resulting from sources other than caramel colours and beverages made from dried plums) and NOAEL values in the range of 80–100 mg/kg bw in animal studies was unlikely to indicate a safety concern (Abraham et al. 2011b).

#### Existing knowledge on exogenous sources

##### Exogenous exposure from food

For the general population, intake via heat-treated or fermented foods and beverages is considered to present the main exogenous source of exposure to dicarbonyl compounds. Glyoxal has, for instance, been found at significant concentrations in beer and wine, sherry, and Bourbon whiskey as well as in instant and brewed coffee. Soy products, bread, yoghurt, and edible oils are also considered to be important contributors to glyoxal exposure via food. Similarly, soy and dairy products, bread and bakery products, alcoholic drinks and coffee consumption are key sources of dietary exposure to methylglyoxal. Particularly high concentrations of methylglyoxal have been detected in manuka honey. In a recent comprehensive analysis of dicarbonyls in commonly consumed foods and drinks, 3-deoxyglucusone was found to be the major dicarbonyl in most foods and drinks analysed (Maasen et al. 2021). Based on the average daily consumption of food items by the Dutch population, the daily dicarbonyl intake was reported to be about 3 mg for both glyoxal and methylglyoxal, and approximately 9 mg for 3-deoxyglucosone, with bread, beer, cookies and bakery products, and soft drinks identified as main contributors to dietary 3-deoxyglucusone exposure (Maasen et al. 2021). These values are slightly lower than dietary exposure estimates of 5–20 mg methylglyoxal/day and 20–160 mg 3-deoxyglucosone/day in a German population (Degen et al. 2012), as well as the worst-case intake of 10 mg glyoxal/day estimated by WHO (2004).

Based on the content of selected Maillard reaction products in food and assuming consumption of 1 L of milk and 500 g of bakery products, dietary exposure of consumers of a typical “Western diet” has been roughly estimated at 500–1000 mg/day for Amadori products (such as *N*-*ε*-fructoselysine) and 25–75 mg/day for advanced Maillard reaction products, mainly carboxymethyllysine (1–5 mg/day) and pyrraline (1–10 mg/day) (Hellwig and Henle 2014; Henle 2003).

##### Exogenous exposure from other sources

Glyoxal is also found in the environment, whereby residential wood combustion and vehicle exhaust appear to be the main sources for glyoxal in ambient air. Based on its disinfectant properties, glyoxal is also present in some household cleaners. Its use as a disinfectant is also a key source of occupational exposure. Electronic cigarette and regular tobacco cigarette smoke are a further exogenous source of glyoxal and methylglyoxal exposure. Sterilising glucose solutions such as peritoneal dialysis and infusion fluids by heat gives rise to a range of glucose degradation products, including 3-deoxyglucosone, glyoxal, methylglyoxal, and 5-hydroxymethylfurfural (Haybrard et al. 2017; Pischetsrieder et al. 2016).

#### Existing knowledge on endogenous sources

**Fig. 8:**
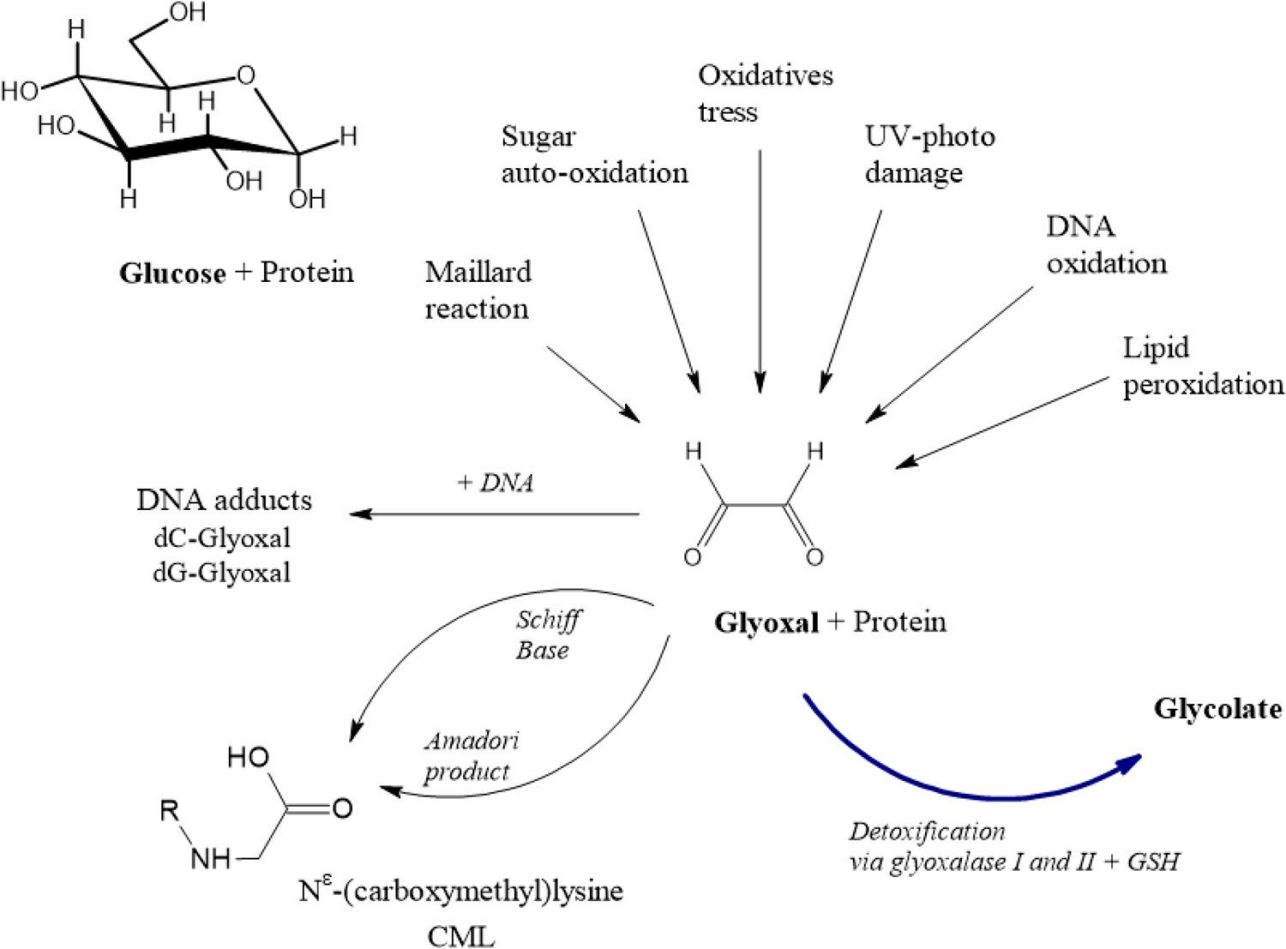
Pathways of endogenous formation of glyoxal, reaction with cellular nucleophiles, and detoxification via the glyoxalase system (Adapted from WHO 2004)

While there are as yet no data, e.g. from analysis of 5-HMF derived DNA adducts in cells or human tissue samples (Monien et al. 2012, 2015), to suggest endogenous formation of 5-HMF, there is a wealth of evidence that the same reactive dicarbonyls and other glycation compounds formed in thermally processed food via the Maillard reaction are produced in vivo. Both glyoxal and methylglyoxal are formed endogenously via a variety of enzymatic reactions as well as by enzyme independent pathways, including spontaneous reaction of reducing sugars and free or protein-bound amino acids, oxidation of sugars and DNA, and peroxidation of polyunsaturated fatty acids (Fig. 8) (Kalapos 1999; WHO 2004). Methylglyoxal for instance is non-enzymatically formed by decomposition of glyceraldehyde and dihydroxyacetone as well as from acetoacetate (Kalapos 1999; Richard 1993). Similarly, 3-deoxyglucosone is formed endogenously from glucose via the Maillard reaction and the polyol pathway (Niwa 1999). There is also evidence that the gut microflora may present an additional source of endogenous methylglyoxal production (Baskaran et al. 1989). Increased intake of dietary sugars leads to enhanced endogenous production of dicarbonyl compounds.

It has been estimated that 3 mmol methylglyoxal/day are endogenously formed in human tissues (Rabbani and Thornalley 2015). Concentrations of glyoxal, methylglyoxal and 3-deoxyglucosone are typically in the range of 50–150 nM in human plasma and 1–4 μM in mammalian cells (Rabbani and Thornalley 2015). Efficient detoxification of glyoxal/methylglyoxal via glyoxalase as well as covalent binding to proteins limit the concentration of free dicarbonyls in tissues and body fluids.

In analogy to the Maillard reaction that occurs in food, subsequent reaction of electrophilic dicarbonyl compounds with free or protein-bound amino acids gives rise to amino acid derivatives such as CML and pentosidine, which have been shown to accumulate with age and increase in diabetes. The extent of protein glycation by dicarbonyls in normal tissues is reported to be 1–5% (Rabbani and Thornalley 2015).

#### Available biomarkers

While there is concern over the use of some methods such as immunological methods or measurement of fluorescence to indicate an “AGE content” (Hellwig et al. 2019), individual glycation compounds can be quantitatively analysed in biological matrices using high-pressure liquid chromatography (HPLC) with derivatisation, LC or GC/MS and may thus serve as biomarkers of exogenous and endogenous exposure.

Reported levels of methylglyoxal in plasma of healthy individuals generally range between 0.12 and 3.3 µM (Kalapos 2013). Comparative analysis of the concentration of methylglyoxal in diabetic patients revealed significantly elevated levels in the range of 0.19–5.9 µM (Kalapos 2013). The concentration of glyoxal in human plasma of healthy human subjects was reported to be 0.1 µM, whereas higher blood levels were found in patients suffering from diabetes or renal failure. In urine of healthy individuals, concentrations of glyoxal were reported to be 132 µM (Espinosa-Mansilla et al. 1998), which was considered to contradict the low levels detected in plasma (WHO 2004). For methylglyoxal, the same study reported a urinary concentration of 15 µM in urine of healthy individuals (Espinosa-Mansilla et al. 1998). Quantitation of 3-deoxyglucosone levels in human plasma demonstrated a significant increase from 58.5 ± 14 nM in normoglycemic individuals to 98.5 ± 34 nM in type I diabetics (Lal et al. 1997).

Quantitative screening of AGEs in healthy individuals indicated plasma levels of individual glycated free amino acids in the nM range, e.g. 23 ± 8 nM for carboxymethyllysine (CML), 110 ± 46 nM for *N*^δ^-(5-hydro-5-methyl-4-imidazolon-2-yl)-l-ornithine (MG-H1) and 147 ± 19 nM for hydroimidazolones derived from 3-deoxyglucosone (Thornalley et al. 2003). In urine, the concentration of major glycation compounds was reported to be in the low micromolar range (Thornalley et al. 2003). It is presently not clear to what extent glycation compounds from external sources contribute to the levels of dicarbonyls and glycated amino acids in body fluids.

In addition to the free amino acid derivatives, glycated proteins may also be used as biomarkers of exposure. In healthy human subjects, the concentration of individual glycated amino acid residues in plasma protein was reported to be 21 ± 5 µmol/mol of Lys for carboxymethyllysine (1.1 µM), 350 ± 83 µmol/mol of Arg for hydroimidazolones derived from 3-deoxyglucosone (5.9 µM), and 920 µmol/mol of Arg for MG-H1 (15.5 µM) (Thornalley et al. 2003). Significantly increased concentrations of glycated residues in plasma protein were found in uremic patients (Thornalley et al. 2003).

Glycated Hb, in which the N-terminal valine residue of the β chain is glycated and exists as *β*-*N*-1-(deoxyfructosyl)-valine, designated as HbA1C, is a well established and routinely used clinical marker for retrospectively assessing glycaemic control based on its ability to reflect the cumulative glucose exposure over the previous 3 months (Ribeiro et al. 2016). In addition to Hb or plasma proteins, levels of individual glycated amino acid residues such as carboxymethyllysine bound to proteins with low turnover (e.g. collagen, connective tissue, and eye lenses) may be considered as markers for chronic dicarbonyl exposure.

Since electrophilic dicarbonyl compounds readily react with DNA bases, stable DNA adducts formed from glyoxal, methylglyoxal and 3-deoxyglucosone present in DNA extracted from whole blood and tissues or excreted as nucleosides in urine may serve as biomarkers of exposure. A pilot study quantifying 9 exocyclic DNA adducts induced by 8 aldehydes in genomic DNA isolated from the blood of a smoker compared to a non-smoker surprisingly showed lower levels of adducts arising from methylglyoxal, i.e. *N*^2^-(1-carboxyethyl)-2′-deoxyguanosine and *N*^2^-(1,2-dihydroxy-2-methyl)ethano-2′-deoxyguanosine in the smoker (Alamil et al. 2020). In tissues, *N*^2^-(1-carboxyethyl-)2-deoxyguanosine was detected in a human breast tumour and normal adjacent tissue at levels of 3–12 adducts/10^7^ dG (Synold et al. 2008). Using an antibody-based approach, *N*^2^-(1-carboxyethyl)-2′-deoxyguanosine was also found in urine of healthy individuals, suggesting that analysis of urinary methylglyoxal-derived DNA adducts may be used to monitor exposure to this dicarbonyl compound (Schneider et al. 2004). Treatment of WM-266-4 human melanoma cells with methylglyoxal at concentrations > 50 µM (3 h) or glucose (> 5 mM, 5 days) resulted in a dose-dependent increase in *N*^2^-(1-carboxyethyl)-2′-deoxyguanosine (Yuan et al. 2008). Of note, *N*^2^-(1-carboxyethyl)-2′-deoxyguanosine was also detected in untreated cells at a level of one lesion per 10^7^ nucleosides, and exposure to 10 µM methylglyoxal for 3 h did not cause a significant increase above this background level (Yuan et al. 2008).

#### Impact of endogenous formation on risk assessment

With the exception of a risk characterisation based on a hypothetical worst-case exposure for dietary glyoxal intake conducted by WHO in 2004 which suggested low concern but did not consider endogenous formation (WHO 2004), the risks related to dietary exposure to dicarbonyl compounds and other glycation compounds have not been assessed to date. In contrast to the inherent toxic potential of electrophilic dicarbonyls, there are no or very limited reliable data to suggest that dietary-free and protein-bound glycated amino acid derivatives produce adverse effects, despite a wealth of data to show that AGEs formed endogenously contribute to ageing and disease.

Considering the extent of endogenous formation of reactive dicarbonyls (exemplified by 3 mmol endogenous production of methylglyoxal per day compared to an estimated 0.04–0.27 mmol/day intake via food) and their efficient detoxification, it appears that the impact of dietary dicarbonyl exposure to the overall body burden may be relatively low or even negligible for some compounds. Thus, a critical issue in risk assessment particularly of dietary dicarbonyls, which are clearly genotoxic and potentially carcinogenic, is to benchmark the additional body burden due to dietary intake against the background of endogenous formation. This is particularly important with regard to dicarbonyl-derived DNA adducts such as *N*^2^-(1-carboxyethyl)-2′-deoxyguanosine, which exhibited an endogenous background level of one lesion per 10^7^ nucleosides in a mammalian cell line that was not significantly affected by exposure to 10 µM methylglyoxal.

### *N*-Nitroso compounds

#### Characterisation, formation, occurrence, and public health concern

Knowledge about chemical and biological properties of *N*-nitroso compounds (NOC) dates back to the early 50 s of the twentieth century. The strong carcinogenic potential of N-nitroso-dimethylamine (NDMA) was first described in 1956 (Magee and Barnes 1956) and was independently confirmed somewhat later (Schmähl and Preussmann 1959). The next homolog, *N*-nitroso-diethylamine (NDEA) was found to be equally or even more potent (Schmähl et al. 1960) and this finding triggered systematic structure–activity and dose–response studies on these compounds, primarily in Germany (Druckrey et al. 1967), soon also in research centres all over the world, resulting in close to about 400 compounds described chemically and biologically. Carcinogenicity and other biological effects were found to depend on structural features, dose, and route of application. Overall, close to 90% of NOC were found to be mutagenic and carcinogenic in vitro and in vivo, suggesting that any newly discovered or synthesised NOC will be highly probable to be carcinogenic.

There are two main categories of NOC: *N*-nitrosamines and *N*-nitrosamides. Whereas *N*-nitrosamines are chemically rather stable, *N*-nitrosamides (encompassing, e.g. *N*-nitrosoureas, -carbamates and -guanidines) are less stable and may spontaneously decompose, generating electrophilic (mostly alkylating) species, a decomposition governed by pH and other (physico-/bio-) chemical conditions and the respective NOC structure. Whereas nitrosamines require metabolic activation, nitrosamides in most cases are directly acting genotoxic agents.

**Fig. 9:**
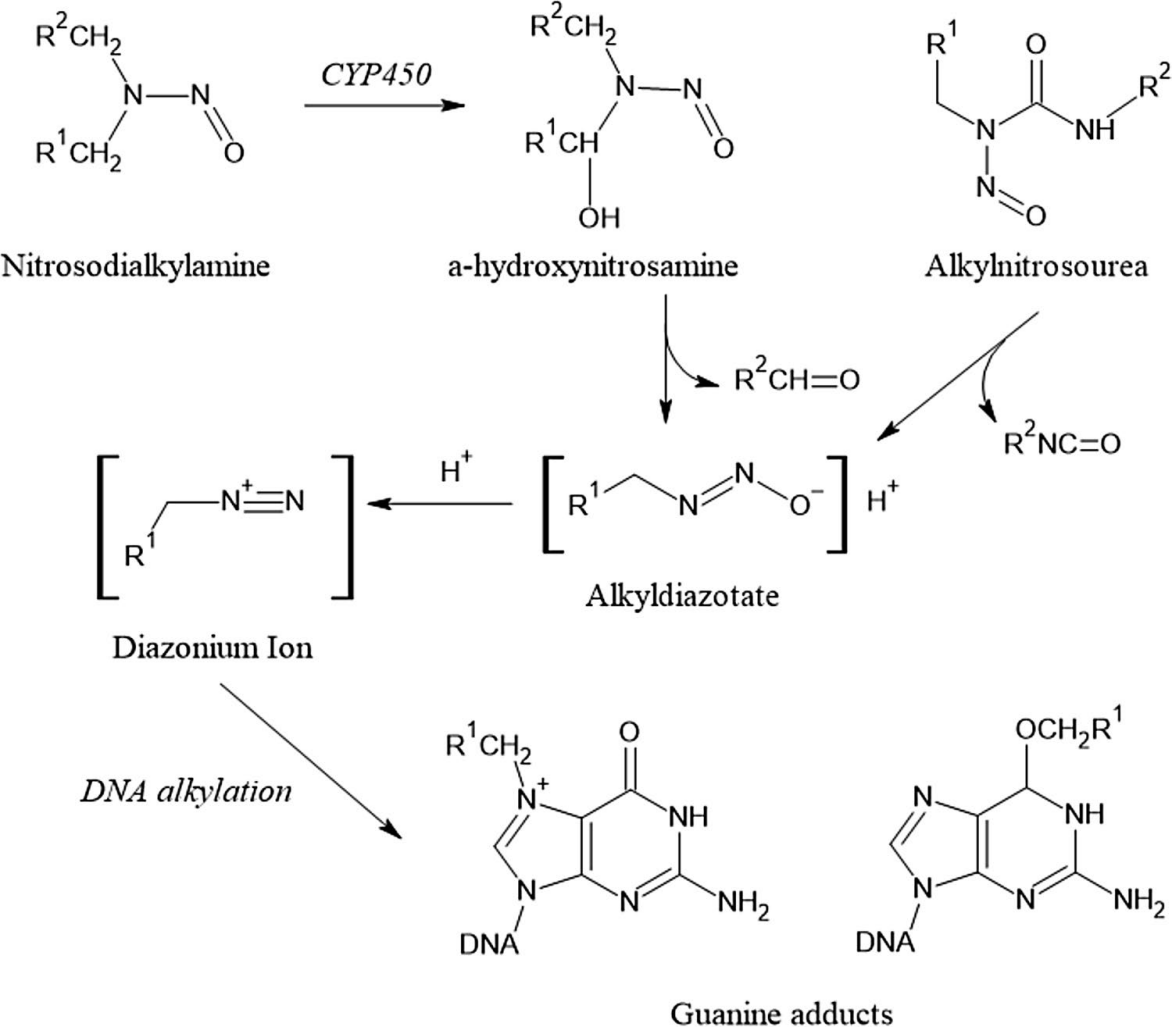
Metabolic activation of *N*-nitroso compounds

The metabolic activation of dialkyl nitrosamines occurs through CYP450 dependent insertion of oxygen into an α C–H bond, resulting in the formation of a rather short lived *α*-hydroxy-*N*-nitrosamine. The *α*-hydroxy-*N*-nitrosamine cleaves off an aldehyde to generate an alkyldiazonium electrophile that may alkylate DNA at nucleophilic positions, predominantly at *N*^7^ of guanine, to a minor extent also at oxygens of DNA bases such as *O*^6^ of guanine or *O*^2^ and *O*^4^ of thymine (Fig. 9). The oxygens of the phosphate diester nucleotide backbone may undergo alkylation as well. The adducts to the oxygens of DNA bases are potently mutagenic DNA lesions, whereas adducts at nitrogens, especially at *N*^*7*^ of guanine are much less promutagenic, if at all.

**Fig. 10:**
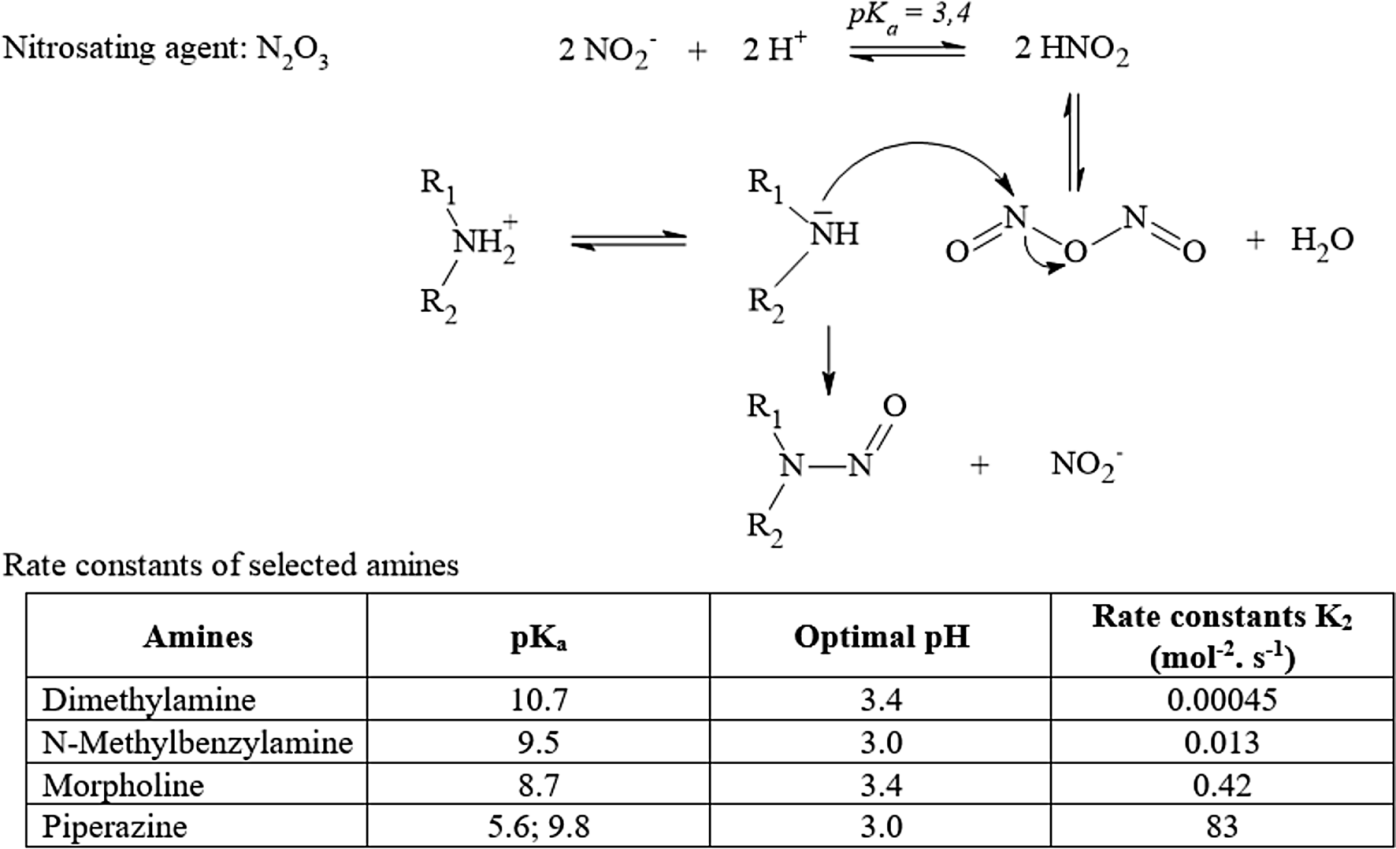
Nitrosation of secondary amines by nitrous acid

As a rule of thumb, it can be stated that in practical, all situations where nitrosating agents encounter *N*-nitrosatable amino compounds, NOC may be formed, be it in the human environment, at working places or even in the human organism. In aqueous-acidic environments, amines react with nitrous acid to form NOC under proton catalysis, which is particularly relevant for the formation of nitrosamines in the gastric lumen. Nitrite equilibrates with nitrous acid (HNO_2_, pKa 3.4) and, favoured by enhanced proton concentration 2 molecules of undissociated HNO_2_ generate the actual nitrosating species, dinitrogen trioxide (N_2_O_3_). Other nitrosating species may become operative as well, depending on pH and the presence of further ionic and/or reactive oxygen species, including nitrogen tetroxide (N_2_O_4_) or nitrous acidium (nitrosonium)ion (NO^+^) the latter in charge-balance with counteranions such as halogenides, (hydrogen) carbonate, superoxide- or peroxo-species. The mechanism of nitrosation is exemplified for a secondary amine in Fig. 10 (Mirvish 1975).

Since only the unprotonated amine nitrogen is available for *N*-nitrosation, the pKa value of a given amine governs the nitrosation rate. Strongly basic dialkylamines (pKa > 9.5) have their maximal nitrosation rate at around pH 3.4, corresponding to the pKa value of HNO_2_ (Mirvish 1975). The rate decreases with higher proton concentration since protonation limits free amine concentration, whereas at higher pH the concentration of N_2_O_3_ becomes rate limiting, since the proton concentration primarily governs N_2_O_3_ formation from 2 molecules of undissociated HNO_2_. Thus, the rate dependence on pH is represented by a bell-shaped curve with a maximum at about pH 3.4. This also explains why in aqueous-acidic media (e.g. at pH 3.4) weakly basic amines, such as morpholine (pKa 8.7) are much more rapidly nitrosated than strongly basic ones like dimethylamine (pKa 10.7). In the presence of aldehydes, especially formaldehyde, nitrosation is strongly catalysed and proceeds even at neutral or basic pH values. This has been found relevant for certain environmental and especially specific working place conditions characterised by co-occurrence of secondary amines, formaldehyde, and nitrogen oxides (NOx) (Keeper and Roller 1973). Tertiary amines may also generate nitrosamines by a reaction termed dealkylating nitrosation, however, in general this occurs at substantially lower reaction rates as compared to nitrosamine formation from secondary amines (Loeppky et al. 1990). Nitrosation of primary amines in aqueous solution results in unstable diazonium intermediates that may react with water to alcohols and/or with biological nucleophiles by covalent adduct formation, as may be conceptualised by formation of diazoacetate, a carboxymethylating intermediate from nitrosation of the amino acid glycine.

Finally, the nitrosation reaction can be inhibited by NOx scavengers, such as ascorbic acid, α-tocopherol, flavonoids, tannins and other phenolics, whereas halogenides and thiocyanate act as catalysts (Loeppky et al. 1994).

#### Existing knowledge on exogenous sources

##### Exogenous exposure from food

In 1964, NDMA was identified as causative agent for induction of hepatotoxicity in sheep fed on herring meal produced from sodium nitrite-preserved herring, apparently at rather high concentrations (Ender et al. 1964). Although appropriate analytical methods were not available at that time, NDMA was identified, supposed to have arisen from the treatment of the fishmeal with nitrite. These early findings raised the suspicion that NOC might also be present in nitrite- or nitrate-treated food for human consumption, such as cured meat products and the like. Analytical studies to uncover the presence of NOC initially were hampered by insufficient specificity and sensitivity but became possible with the advent of highly specific and sensitive detection methods, such as the thermal energy analyzer (TEA) (Fine and Rounbehler 1975) and GC or LC coupled with multimode MS (LC–MS/MS/MS). NOC contamination was proven in many foods, feeds, cosmetics, consumer goods, pesticides, drugs and at working places involved in production and handling of such items, mostly (but not always) in rather low concentrations.

These investigations led to unveiling major causes underlying NOC generation in food, including food processing such as nitrite/nitrate-mediated curing/brining of meat and fish, drying and kilning with direct firing techniques, smoke treatment of food and, in rare cases, migration of NOC from packaging materials into food. Mitigation measures taken to reduce contamination encompassed substantial decrease of added nitrite and nitrate, addition of inhibitors such as ascorbic acid, tocopherols and others to curing mixtures and foods and reducing NOx contents in smoke for food smoking, as far as technically feasible. Direct firing techniques were modified to minimise NOx generation or replaced by (traditional) indirect heating of the drying air in malting kilns and by avoidance of environmental NOx impact. These mitigation measures contributed to achieve a marked reduction in consumer exposure, evidenced by dietary intake estimates for NDMA of 0.03–0.11 µg/day for the US (Fristachi and Rice 2007; Hrudey et al. 2013) and up to about 0.3 µg/day for various European countries (Biaudet et al. 1994; Dich et al. 1996; Jakszyn et al. 2004; Tricker and Preussmann 1991).

##### Exogenous exposure from other sources

For non-food-related exposure, products of personal care and cosmetics are of some relevance. The NOC most often identified in cosmetics is *N*-nitrosodiethanolamine (NDELA), but sporadically also other NOC have been found, such as *N*-nitroso-morpholine (NMOR), -diethylamine (NDEA), -di-2-hydroxypropylamine (NDPA) and certain *N*-nitroso-methyl-long chain-alkylamines. The contamination resulted primarily from use of secondary amines, from insufficient purity of materials as a consequence of inadequate purity specifications and from (inadvertent) contact with nitrosating agents during production and/or storage. In some cases, the presence of certain preservatives like Bronopol/Bronidox, both acting as potent nitrosating agents, was found to cause substantial build up of NOC contamination in cosmetics. Mitigation measures were taken, consisting mainly of introduction of purity specifications for basic materials, such as mono- and trialkanolamines, prohibition of the use of secondary amines and the avoidance of any nitrosating agents during production and storage (SCCS (Scientific Committee on Consumer Safety) 2021). This regulation led to substantial reduction of contamination, e.g. down to an estimated average systemic consumer exposure to NDELA through dermal application of cosmetics of < 0.05 µg/person/day (Janzowski et al. 2000).

Occupational exposure of workers at specific working places in the rubber, leather, chemical, and metal industry, by inhalation or through dermal contact, was discovered as a major source of non-consumer related exposure. For example, cutting fluids widely used in metal working industries were found to be contaminated with up to high ppm levels of nitrosamines such as NDELA, NMOR and *N*-nitroso-1,3-oxazolidines, the latter arising by three component condensation from primary alkanolamines, formaldehyde (used as biocide) and nitrite (used as anticorrosion agent) during long-term use (Ellen et al. 1982; Fajen et al. 1979; Stephany and Schuller 1978). Mitigation measures primarily encompassed minimisation of contact with nitrosating agents, use of nitrosation inhibitors and NOx scavengers to prevent NOC formation, as well as replacement of chemicals giving rise to carcinogenic NOC by those that upon *N*-nitrosation yield noncarcinogenic NOC (so-called “safe amines”). These mitigation measures have been laid down, together with further guidance to protect workers at working places, in technical rules for hazardous substances (GMBI TRGS 552 2018). They provide, within an extensive framework of protective and preventive rules and standards, regulation for working places in relevant industrial situations carrying a risk of NOC exposure of workers. A selection of such technical rules for a variety of critical working places is given in Table 1 (GMBl 2018).

**Table 1:** Technical rules for working places carrying a risk of NOC exposure of workers (GMBl 2018)

Industry	NOC	Critical working place	Tolerance/acceptance conc. Indiv.NOC or Σ
Metal	NDELA/NDMA/NMOR	Cutting, grinding, drilling	0.75/0.075 µg/m^3^
Rubber	NDMA/NDEA/NMOR/NPIP	Vulcanisation and subsequent processes; storage	0.75/0.075 µg/m^3^
Chemical	NDMA/NDEA/NMOR/NPIP	Use of dimethylfomamide/tank/reactor filling	0.75/0.075 µg/m^3^

Tobacco consumption is another source of substantial exogenous exposure, especially to tobacco-specific NOC formed from the main tobacco alkaloid, nicotine. The tertiary amine nicotine upon *N*-nitrosation generates three NOC by dealkylating nitrosation of the *N*-methylpyrrolidine part of the molecule, namely *N*-nitroso-nornicotine (NNN), 4-*N*-methyl-*N*-nitroso-4-(3-pyridyl)butanal (NNAL) and 4-*N*-methyl-4-(1 pyridyl)-1-butanone (NNK). NNK and NNAL have been concluded to be carcinogenic to humans, thus representing Group 1 carcinogens, based on sufficient evidence for carcinogenicity in animals and strong mechanistic evidence in exposed humans (IARC 2007)*.* Beyond direct exposure of smokers and/or consumers of other types of tobacco products (e.g. chewing tobacco) to substantial levels of these NOC, extensive biomarker monitoring also has shown noteworthy passive exposure of non-smoking population groups to tobacco-specific NOC present in environmental tobacco smoke (ETS). This may apply to vulnerable groups such as the unborn and/or newborn, e.g. by transplacental exposure, but also to elementary school children and women living with smokers (Hecht 2003). Similar passive exposure has further been proven for specific exposure situations, exemplified by subjects in tobacco smoke contaminated rooms, such as people in a gambling casino. Typical levels of NNAL and NNAL-glucuronides excreted in the urine of passively exposed non-smokers were about 1–5% of those in smokers. For ETS exposure, such data have been considered to be consistent with a causal link between exposure and lung cancer risk (Hecht 2003).

#### Existing knowledge on endogenous sources

It is well established that in the human organism nitrosating agents are permanently generated. Nitrite may arise from nitrate ingested with the diet by chemical and/or microbiological reduction. Orally ingested nitrate is rapidly absorbed and systemically distributed via the blood circulation. Wherever it encounters reducing enzymology in the mammalian host and/or its microbiome, it will undergo partial conversion into nitrite and/or NOx.

One illustrative example is provided by the human oral cavity: ingested nitrate after absorption from the upper gastrointestinal tract and distribution through the blood circulation reaches the salivary glands, where nitrate is secreted by active transport from blood into saliva, achieving salivary nitrate levels up to 20 times the plasma level. In the oral cavity, salivary nitrate is partially converted into nitrite by oral and commensal microbial reductases (Eisenbrand et al. 1980). Approximately, 25% of the orally ingested nitrate is secreted through the salivary glands and up to about 7% of the totally ingested nitrate becomes converted to nitrite in the oral cavity during enterosalivary circulation. Saliva-derived nitrite may contribute to NOC formation in the stomach when *N*-nitrosatable compounds are present.

In addition to nitrite, NOx may function as further in vivo nitrosating species. In contrast to nitrite that requires an acidic environment to generate NOC, NOx can give rise to NOC under neutral or even basic conditions. Amongst NOx, nitrogen monoxide (NO) is preeminent because it works as a multifaceted physiological signalling agent that is consistently generated from l-arginine by a family of oxygen-dependent NO synthases (Hattori et al. 1994; Marletta et al. 1988; Moncada and Higgs 1993). NO is also a key component formed in response to bacterial infections and/or during inflammatory reactions (Hussain et al. 2008; Stuehr and Marletta 1985). Nitrate and nitrite, whether endogenously synthesised or taken up from exogenous sources, are known alternative sources for endogenous NO.

NO itself is not a nitrosating agent, but may rapidly react to NOx in the presence of oxygen. A variety of enzymes and proteins act as reductases, giving rise to NO from nitrate/nitrite, including flavoproteins and CYP450, deoxygenated Hb/myoglobin, xanthine oxidase and mitochondrial respiratory chain enzymes, among others (Cosby et al. 2003; Lundberg and Govoni 2004; Millar et al. 1997; Reutov and Sorokina 1998; Shiva et al. 2007; Zhang et al. 1998). In the mammalian organism, nitrate, nitrite and NOx are metabolically interconvertible (Leaf et al. 1989; Weitzberg and Lundberg 1998). It has been shown that NO can endogenously be oxidised to nitrate and nitrite (Leaf et al. 1989; Marletta et al. 1988)*,* whereas the latter two can undergo reduction to partially cycle back to bioactive NO in blood and tissues before terminal excretion, e.g. as urinary nitrate*.* In humans, nitrate excreted in urine has been reported to exceed the amount ingested by a factor of about 4; endogenous nitrate biosynthesis in humans was found to reach about 10 μmol/kg bw/day which is equivalent to 0.7 mg/kg bw/day or 50 mg/day for a 70 kg person (Green et al. 1981; Tannenbaum et al. 1978)*.*

The IARC concluded that there is inadequate evidence in experimental animals for the carcinogenicity of nitrate and limited evidence for the carcinogenicity of nitrite (IARC 2010b). However, IARC also stated that there is sufficient evidence in experimental animals for the carcinogenicity of nitrite in combination with amines or amides, through NOC formation in the organism (IARC 2010b).

It is well established that also numerous drugs after their absorption and distribution through the blood circulation are secreted into saliva, with salivary levels often directly reflecting blood levels. This allows, as confirmed in many cases, monitoring of blood levels of the parent drug and/or its metabolite(s) through measurements in saliva. Given a simultaneous or prior nutritional nitrate uptake in amounts as brought about, e.g. by the consumption of nitrate-rich vegetables, dietary nitrate may give rise to endogenous nitrite, thus enhancing the risk of endogenous NOC formation. Drugs for which this in vivo *N*-nitrosation in saliva and gastric juice has compellingly been documented, are, for instance, amidopyrine and piperazine (Bellander 1990; Bellander et al. 1985; Spiegelhalder and Preussmann 1985). Not only drugs and their metabolites, but also food constituents may at least partially share the described biokinetic properties, in other words undergo enterosalivary circulation following absorption from the gastrointestinal tract.

Beyond undergoing enterosalivary circulation, nitrosatable food constituents may be prone to endogenous *N*-nitrosation when encountering nitrosating agents anywhere in the body. For the gastrointestinal tract, endogenous NOC formation has been exemplified especially for secondary amino acids such as proline, hydroxyproline (Ohshima et al. 1982) as well as thiazolidine-4-carboxylic acids and congeners (Ohshima and Bartsch 1988; Ohshima et al. 1984). The corresponding NOC, formed from the respective amino acids following nutritional uptake and/or during digestion, are excreted fairly quantitatively in the urine and are not carcinogenic. In volunteers, ingestion of nitrate has been demonstrated to lead to enhanced urinary excretion of *N*-nitrosated amino acids (Knight et al. 1991; Tricker and Preussmann 1987).

#### Available biomarkers

Since the above-mentioned *N*-nitrosated amino acids, exemplified by *N*-nitrosoproline, are neither mutagenic nor carcinogenic, they are considered as valuable surrogate biomarkers for the in vivo formation of potentially carcinogenic NOC, primarily in the gastrointestinal tract. However, whether the excretion of *N*-nitrosated proline, hydroxyproline and their *N*-nitrosated thiazolidine congeners may truly reflect the overall endogenous nitrosation potential, including potential generation of carcinogenic NOC is not yet conclusively proven.

For example, gastrointestinal disorders like chronic atrophic gastritis and status after stomach resection are characterised by achlorhydric gastric fluid with the consequence of bacterial growth in the stomach. Such disorders have been identified as precursor lesions for gastric cancer (Correa et al. 1990).

Nitrite concentrations in the gastric fluid have been shown to be (directly) correlated with intragastric pH. Under conditions of hypochlorhydria, bacterial contamination of gastric fluid is common and nitrate reducing microorganisms are regularly found, causing raised nitrite concentrations considered to favour the intragastric formation of *N*-nitroso compounds. In a human intervention study, volunteers ingested (after 12 h fasting) 200 mg nitrate, followed (30 min later) by 500 mg l-proline. Urinary *N*-nitrosoproline (NPRO) excretion over the following 24 h in most cases showed no detectable NPRO in the urine of achlorhydric patients with gastric resection, whilst substantial NPRO excretion was detected in healthy controls. Thus, given that formation of NPRO in the stomach is governed by acid concentration, urinary NPRO excretion may not function as surrogate biomarker for potentially enhanced risk of overall endogenous NOC formation. The latter may occur not only under acidic stomach conditions (pH < 4) but also at body compartments not accessible to proline/*N*-nitrosoproline. Thus, as surrogate biomarkers for overall endogenous nitrosation urinary excretion of *N*-nitrosated amino acids may be of limited use. Therefore, appropriate methodology has still to be developed for a comprehensive assessment of endogenous nitrosation.

An alternative biomarker-based approach concentrates on monitoring DNA base adducts resulting from alkylating NOC. As key mutagenic DNA lesion, *O*^6^-methylguanine formation has been exploited for dosimetry of NDMA-induced DNA damage. *N*-nitrosoglycocholic acid (and other nitrosated glycine derivatives), have also been investigated as potential biomarkers reflecting endogenous nitrosation. These compounds are known to form several DNA adducts, including *O*^6^-carboxymethyl-guanine and *O*^6^-methylguanine (Harrison et al. 1999; Shuker and Margison 1997).

The other authors have proposed dihydrouracil (DHU) as a physiological model compound that may be of value for dosimetry of endogenous nitrosation potential. DHU is a normal intermediate in mammalian metabolism, formed from uracil by dihydropyrimidine dehydrogenase and is present in human plasma and urine. Levels of DHU excreted in the urine of healthy humans have been reported to range from about 2 to 10 mg/day. The corresponding NOC, *N*-nitrosodihydrouracil (N-DHU) is a hepatocarcinogenic, DNA carboxyethylating agent. In human leukocyte DNA samples, an average amount of 7-carboxyethyl-guanosine (7-CEGua) of 103 ± 89 fmol/μmol Gua has been reported (Bulay et al. 1979; Wang et al. 2013). Thus, DNA carboxyethyl adducts may as well be eligible as endogenous nitrosation biomarkers, although they are known to also be formed by other agents, including acrolein and/or acrylic acid.

In human monitoring studies, levels of *O*^6^-methylguanine have been measured by immunochemical methodology in peripheral leukocytes and have been estimated to be associated with overall exposure to NDMA at a mean level of 18 μg/kg/day, up to a maximum of 220 μg/kg/day (ca. 17,000 μg/day). This surprisingly high estimate, supposedly to a major extent caused by endogenous NDMA formation, obviously will require further confirmation (Georgiadis et al. 2011).

Some support for such high estimates may be found by extrapolation from human NDMA blood levels reported in the literature (Dunn et al. 1986; Gough et al. 1983; Simenhoff et al. 1982). On the premise, they reflect steady-state conditions and assuming an NDMA clearance rate in humans equal to that reported for rats (3.45 L/min), estimates of 100 to nearly 2500 μg/day = 1.4–35 μg NDMA/kg bw/day (based on 71.5 kg bw) were derived (Hrudey et al. 2013). This is concordant with the order of magnitude estimated from endogenous *O*^6^-methylguanine levels in humans. Taken together, these data apparently suggest a much higher human NOC exposure of endogenous origin than previously anticipated. Given that the analytical methodology to determine human NDMA blood levels may have suffered from some uncertainty in terms of reliability and proven absence of artefact formation, the data on human blood levels require definite confirmation.

#### Impact of endogenous formation on risk assessment

Although it has been clearly demonstrated that carcinogenic NOC might be formed endogenously from appropriate precursors, endogenous formation of carcinogenic NOC has not been evaluated quantitatively and comprehensively as a process of potential relevance to human cancer. Data on urinary levels of *N*-nitrosated amino acids are available to a limited extent but they appear to depict exclusively gastric, acid catalysed *N*-nitrosation. Therefore, no conclusive approach to adequately consider systemic endogenous formation of NOC in risk assessment is available at present.

### Ethylene oxide

#### Characterisation, formation, occurrence and public health concern

Ethylene oxide (ethene oxide, oxirane; EO) is an important industrial chemical in the production of ethylene glycol, non-ionic surfactants and other substances; it is also used as a sterilising agent for medical devices.

The IARC has classified EO as being “carcinogenic to humans (Group 1)” (IARC 1994, 2008). This overall evaluation was very much influenced by “supporting evidence” which was summarised as follows:

- “EO induces a sensitive, persistent dose-related increase in the frequency of chromosomal aberrations and sister chromatid exchanges in peripheral lymphocytes and micronuclei in bone-marrow cells of exposed workers;
- EO has been associated with malignancies of the lymphatic and haematopoietic system in both humans and experimental animals; (remark: note, however, that there was no statistically significant increase observed in lymphatic/hematopoietic malignancies in large human cohort studies as detailed below);
- EO induces a dose-related increase in the frequency of Hb adducts in exposed humans and dose-related increases in the numbers of adducts in both DNA and Hb in exposed rodents;
- EO induces gene mutations and heritable translocations in germ cells of exposed rodents; and
- EO is a powerful mutagen and clastogen at all phylogenetic levels.”

The US-EPA completed an evaluation of the carcinogenicity of EO in December 2016. The US-EPA reviewed epidemiologic, laboratory animal, and mechanistic studies pertaining to the human carcinogenicity of EO and addressed some key scientific issues such as the analysis of mechanistic data as part of the cancer hazard evaluation and to inform the quantitative risk assessment. It was concluded that EO is carcinogenic in humans, with the strongest human evidence links to lymphoid and breast cancers. Similar to IARC, US-EPA concluded that analyses of the mechanistic data establish a key role for genotoxicity and mutagenicity in EO-induced carcinogenicity and reveal little evidence supporting other mode-of-action hypotheses. In consequence, EO was flagged as carcinogenic to humans by inhalation, posing a potential human health hazard for lymphoid and breast cancers (US-EPA 2016).

The evaluations of both IARC and US-EPA are being questioned. Thus, Vincent et al. (Vincent et al. 2019) argued that higher quality epidemiological studies demonstrated no increased risk of breast cancers or lymphohaematopoietic malignancies, and that toxicological studies and studies of early effect biomarkers in animals and humans provided no strong indication that EO causes lymphohaematopoietic or mammary cancers. In consequence, animal data were addressed as being inadequate to define the actual dose–response shape or predict tumour response at very low doses with any confidence. The position was defended that the IARC and US-EPA classification of EO as a strong human carcinogen overstated the underlying evidence, so that the carcinogenic risk derived by US-EPA was overestimated.

In essence, the extrapolation of an EO-related increase in human tumour incidence is difficult because epidemiologic data suffer from insufficient statistical power and difficulties to accurately estimate the exposure. Based on essentially the same data, different assessors arrive at dramatically different estimates: the US-EPA (2016) calculates a cancer unit risk of almost 0.01/1 ppb EO, claiming validity for up to 20 ppb, which would accordingly result in 20% additional tumours at this exposure level, which is hardly in line with existing epidemiological data. In sharp contrast, the Texas Commission on Environmental Quality (TECQ 2020) arrives at a cancer unit risk of 0.000004/1 ppb EO, thus estimating a 2500-fold lower risk.

EO is formed from its precursor, ethylene, in laboratory animals (Ehrenberg et al. 1977; Filser and Bolt 1984; Maples and Dahl 2008) as well as in humans (Filser et al. 1992; Törnqvist et al. 1989). Ethylene is a normal body constituent. Its endogenous formation has been evidenced by its exhalation in animals (Sagai and Ichinose 1980; Shen et al. 1989) and in humans (Filser et al. 1992; Ram Chandra and Spencer 1963; Shen et al. 1989). Physiologically based toxicokinetic models for trans-species extrapolation of ethylene and EO are available (Filser and Klein 2018). In essence, EO in trace amounts is a normal body constituent.

**Fig. 11:**
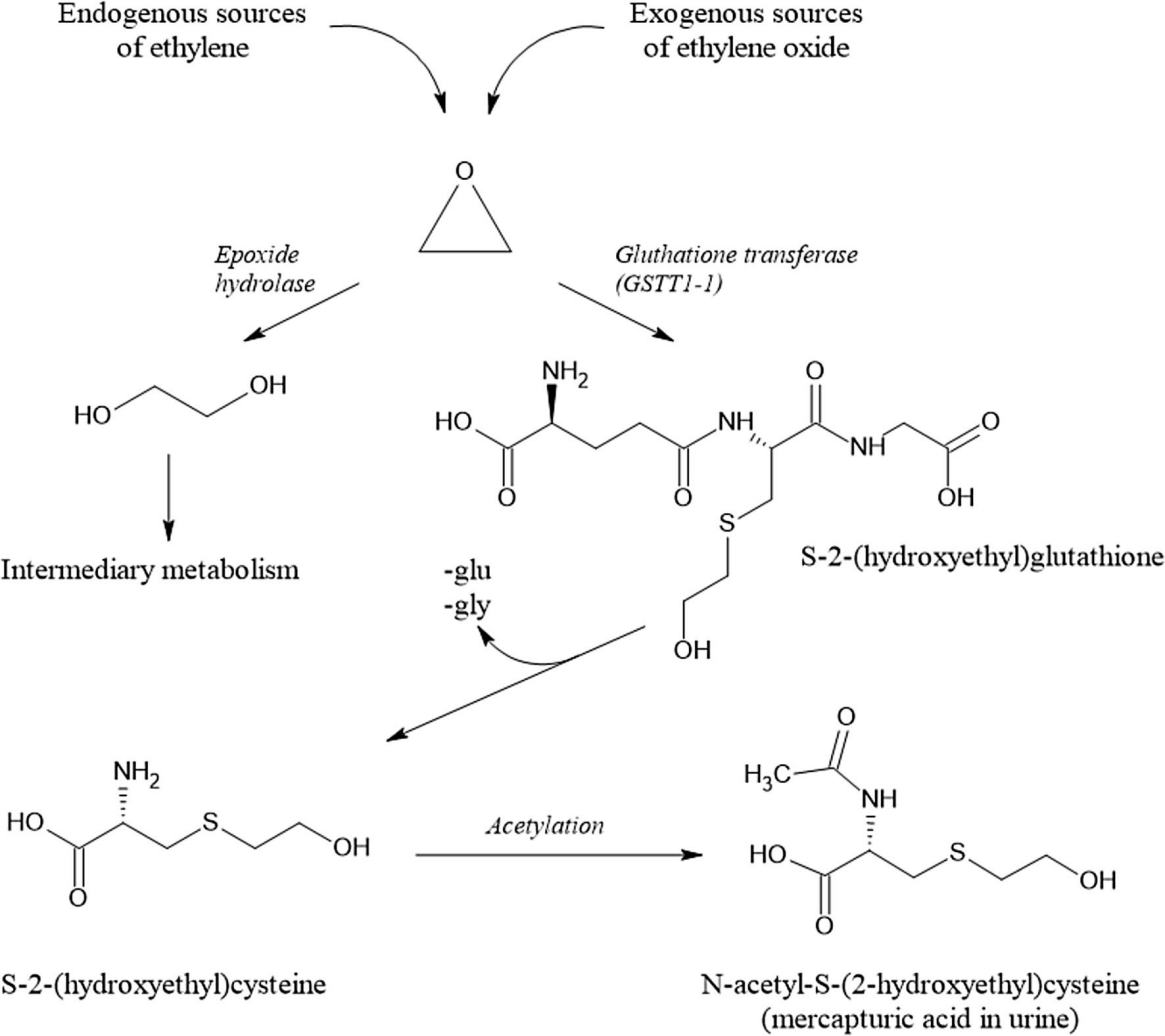
Main metabolic pathways for ethylene oxide (Adapted from Thier and Bolt 2000)

Figure 11 shows the main metabolic pathways: the transformation of ethylene to EO is mediated by CYP2E1. EO is further metabolised by two detoxifying enzymes, epoxide hydrolase and GSH-S-transferase (GSTT1-1).

#### Existing knowledge on exogenous sources

##### Exogenous exposure from food

Historically, potential exposure to EO has been reported from the use of EO in sterilisation of food (Phillips and Kaye 1949). Even today EO is reportedly used in some countries to control insects as a fumigant for spices, seasonings, and foodstuffs. It is employed in some non-thermal decontamination processes for products such as herbs, spices, and seeds. In 2019, Health Canada’s Pest Management Regulatory Agency proposed to establish maximum residue limits (MRLs) for EO on dried vegetables and sesame seeds to permit the sale of foods containing such residues. EO is registered in Canada for use on whole or ground spices and processed natural seasonings. The MRLs proposed in Canada for EO of 7 parts per million are the same as American tolerances (Tarja 2021).

##### Exogenous exposure from other sources

EO is a gas at body temperature (Bp: 10.7 °C) and is of high industrial relevance. It is used primarily in the production of ethylene glycols and derivatives thereof and of ethoxylates. In the health sector, an important application is sterilisation of medical equipment. From such applications, it may be released into the environment. EO has been used as a pesticide (fumigant). Consumers might be exposed to traces of EO as a residue in polyglycol ether preparations, e.g. in skin-care products (Filser et al. 1994). Similarly, the role of packaging materials in EO sterilisation of medicines is a matter of concern (Panella 1974).

The quantification of environmental exposure to EO has been reported. The estimated peak 24 h exposure to EO caused in neighbourhoods close to a sterilisation facility in Michigan was assessed to be 1.83 µg/m^3^ above the background level (Olaguer et al. 2019), which in the United States is reported to vary between 0.18 and 0.4 µg/m^3^ (0.1–0.22 ppb) (US-EPA 2020). In another report, residents living close to two EO emitting facilities in Illinois showed overall geometric Hb *N*-(2-hydroxyethyl)-valine adduct levels of 35.0 pmol/g Hb (29.7 pmol/g Hb for non-smokers) (Szwiec et al. 2020), compared to an average (endogenous) background (Törnqvist et al. 1989) of 20 pmol/g Hb reported earlier (Törnqvist et al. 1989). Thus, occupational and environmental exogenous exposures to EO must be considered (Filser and Klein 2018).

Smokers display higher levels of the Hb adduct *N*-(2-hydroxyethyl)-valine and of hydroxyethyl mercapturic acid excretion compared to non-smokers (Frigerio et al. 2020; Thier et al. 1999; Tornqvist and Kautiainen 1993; Tornqvist et al. 1986). This points to a significant impact of smoking on the total human body burden of EO (see “Impact of endogenous formation on risk assessment”).

#### Existing knowledge on endogenous sources

Reported endogenous sources of ethylene/EO are lipid peroxidation (Frank et al. 1980; Lieberman and Mapson 1964; Sagai and Ichinose 1980; Törnqvist et al. 1989), oxidation of free methionine (Kessler and Remmer 1990; Lieberman et al. 1965), oxidation of haemin in Hb (Clemens et al. 1983) and reductive metabolism of intestinal bacteria (Törnqvist et al. 1989). Furthermore, endogenous EO formation can be fed by exogenous ethylene. Ethylene (the metabolic precursor of EO) is a high-volume gaseous petrochemical Bp: − 169.2 °C) that is used in the production of polymers (primarily polyethylene) and other chemicals. On top, ethylene is an important plant hormone involved in plant growth and development and in fruit ripening. It is commercially used for fruit ripening (e.g. bananas, tomatoes, avocados). Thus, both occupational and environmental exposures to ethylene by inhalation are to be considered (Filser and Klein 2018). Ethylene is transformed to EO by CYP2E1, which partly inactivates this enzyme (suicide inhibition, e.g. by *N*-hydroxyethylation of pyrrole ring D of the CYP porphyrin; (Correia and Ortíz de Montellano 2005; Filser and Klein 2018). Thus, endogenous formation of EO from ethylene is self-limiting.

#### Available biomarkers (ethylene oxide)

##### Hb alkylations

EO reacts with nucleophilic centres of amino acids in proteins, such as cysteine, histidine, or the N-terminal valine in Hb. The most valid measure for individual EO exposure appears to be the N-terminal hydroxyethylvaline adduct of Hb, that integrates EO exposure over a period of several months. Several different calculations for the dependence of valine adduct steady state on external EO exposure fall in the range of 2.5–7 nmol hydroxyethyl valine/g Hb/1 ppm EO exposure under workplace conditions, i.e. 40 h/week (Boogaard 2002; Filser and Klein 2018; Hartwig et al. 2020; Kirman and Hays 2017), thus (more than) a 100-fold above the background in the unexposed population as mentioned above.

##### Mercapturic acid excretion

The characteristic urinary excretion product of EO is the hydroxyethyl mercapturic acid, *N*-acetyl-3-(2-hydroxyethyl)-l-cysteine (Fig. 11). A standard analytical method is available (Schettgen 2013), and this metabolite is routinely used for biomonitoring of acute occupational exposure to EO.

##### DNA adducts

In vitro incubation of calf thymus DNA with radioactive EO yielded 5 adducts (relative yields given in parenthesis), identified as *N*^7^-(2-hydroxyethyl)guanine (85%), *O*^6^-(2-hydroxyethyl)guanine (0.4%), *N*^3^-(2-hydroxyethyl)guanine (4%), as well as two unidentified peaks (3% and 4%) proposed to be *N*^7^-(2-hydroxyethyl)adenine and *N*^1^-(2-hydroxyethyl)adenine (Segerback 1990). Based on the facts that EO (1) is a direct acting DNA modifying compound that does not require metabolic activation, and (2) should easily reach any tissue and penetrate into cells with little barriers on the way, due to its amphiphilic nature and small molecular weight, these in vitro experiments can be expected to reasonably well reflect the in vivo DNA adduct pattern formation by EO.

The type of a DNA adduct, and the cell type in which it is formed, can profoundly influence the mechanisms and efficiency of its repair, and the likelihood that it would lead to a mutation. An identification of specific promutagenic adduct(s) would have a very high impact in risk assessment. However, especially *N*^7^-hydroxyethylguanine and related *N*^7^-alkylguanine undergo spontaneous depurination, because of the positive charge within the imidazole ring. Analytically, this is used for detection, as heating of the DNA preparation releases the *N*^7^-alkylguanine which then can be quantitated. For this reason, practically all available studies on EO-induced DNA are focussed on *N*^7^-hydroxyethylguanine. Out of all potential EO adducts, this is the DNA adduct that is best quantifiable (Thier and Bolt 2000).

Earlier studies in rats (up to the 1990s) showed that DNA alkylation at *N*^*7*^ of guanine, equivalent to the level of normal background alkylation, are caused by repetitive exposures to ethylene oxide at about 1–2 ppm (Bolt 1996). In the 2000s, more sophisticated studies linking DNA adduct levels and exposure to EO were published by Marsden et al. (2007, 2009). In 2007, the authors established a highly sensitive LC–MS/MS assay with selected reaction monitoring that offered a limit of detection of 0.1 fmol of *N*^7^-(2-hydroxyethyl)guanine on column (Marsden et al. 2007). Background levels of *N*^7^-(2-hydroxyethyl)guanine were 1.1–3.5 adducts/10^8^ nucleotides in tissues of rats. Following intraperitoneal administration of a single dose or three daily doses of EO (0.01–1.0 mg/kg), *N*^7^-(2-hydroxyethyl)guanine adducts increased with dose, except at the lowest concentration where total *N*^7^-(2-hydroxyethyl)guanine levels were not different to those detected in control animals, indicating that any increase was negligible as compared to the endogenous damage already present. In a following 3-day study, the kinetics of adduct removal were investigated, and DNA damage did not appear to accumulate with repeated administration.

In a subsequent study, the authors used a dual-isotope approach (Marsden et al. 2009). By combining LC–tandem MS and HPLC accelerator MC analysis, both endogenous and exogenous *N*^7^-(2-hydroxyethyl)guanine adducts were quantified in tissues of (^14^C)EO-treated rats. Levels of (^14^C)*N*^7^-(2-hydroxyethyl)guanine in spleen, liver, and stomach DNA increased in a linear manner from 0.002 to 4 adducts/10^8^ nucleotides. The extent of damage arising through this route was insignificant compared with the background abundance of *N*^7^-(2-hydroxyethyl)guanine naturally present. In reviewing these data, Pottenger et al. (2019) concluded: “The implications of this result are that exogenous *N*^7^-(2-hydroxyethyl)guanine formation may not pose any additional risk over and above that presented by the ubiquitous background damage, at least up to the tested (high, i.p.) doses”. Surprisingly, at the two highest doses tested by Marsden et al. (2009) of 0.05 and 0.1 mg/kg [^14^C]EO, the exposure caused a significant increase in endogenous *N*^7^-(2-hydroxyethyl)guanine formation in liver and spleen, suggesting that EO can induce physiologic pathways responsible for ethylene generation in vivo and thereby indirectly promote *N*^7^-(2-hydroxyethyl)guanine production (Marsden et al. 2009). The authors explained this phenomenon by the involvement of oxidative stress and 1-aminocyclopropane-1-carboxylic acid as a potential biosynthetic precursor to ethylene in mammalian cells.

In general, the *N*^7^-(2-hydroxyethyl)guanine DNA adduct is regarded as a biomarker of exposure, not of a (genotoxic) effect [ILSI/HESI Committee, see (Jarabek et al. 2009)]. Biologically more important may be the *O*^6^-adduct that, however, is only formed in amounts 200-fold below that of the *N*^7^ adduct. It is thought that it is the driving force of EO genotoxicity (Hartwig et al. 2020).

#### Impact of endogenous formation on risk assessment

##### Ethylene risk assessment

Ethylene, the metabolic precursor of EO, has been tested for carcinogenicity by inhalation (up to 3000 ppm) in rats, with negative results (Hamm 1984). Based on the metabolism to EO, and carcinogenicity studies with EO, a first risk estimate for ethylene suggested a carcinogenic risk of 1/100 times that of EO (Bolt and Filser 1984). This was further refined in a later study (Csanady et al. 2000). Based on this analysis, the German MAK Commission of the Deutsche Forschungsgemeinschaft concluded in 1998: “From the results of pharmacokinetic studies it may be deduced that ethylene, like its main metabolite EO, must possess carcinogenic potential for man. However, the substance cannot be classified in Category IIIA1 or IIIA2 (i.e., carcinogens proven in man or experimental animals) because positive results from epidemiology or carcinogenicity studies are not available. Ethylene is therefore classified for the present in Category IIIB” (i.e., suspected carcinogen) (MAK-Commission 2012b).

In the meantime, the underlying PBPK models have been refined (Filser and Klein 2018). From animal and human data experiments, exposures to 10,000 ppm ethylene were predicted to induce the same adduct levels as EO exposures to 3.95 (mice), 5.67 (rats), or 0.313 ppm (humans). This means that a carcinogenic risk from realistic levels of exogenous ethylene exposure would be minimal. The American Conference of Governmental Industrial Hygienists has published a “Threshold Limit Value” for workers exposed to ethylene of 200 ppm (ACGIH 2017).

##### Ethylene oxide risk assessment

Based on the relative contributions of endogenous and exogenous EO to the quantities of the *N*^7^-(2-hydroxyethyl)guanine adduct in DNA, the data by Marsden et al. (2007, 2009) showed that EO (exogenous) *N*^7^-(2-hydroxyethyl)guanine levels in DNA are overwhelmed by endogenous *N*^7^-(2-hydroxyethyl)guanine across a range of EO doses (rats, i.p. injection). This suggests that the impact of endogenous DNA damage would be very high compared to exogenous damage, at least at practically relevant human EO exposure ranges.

However, this contrasts with the findings on Hb alkylation. Investigations in mice, rats and humans using the method of Tornqvist et al. (1986) of monitoring *N*-(2-hydroxyethyl)valine in Hb arrived at backgrounds of 58 ± 10 pmol/g Hb in mice and 42 ± 8 pmol/g Hb in rats (Walker et al. 1992), and 20 pmol/g in (non-smoking) humans. Based on the dose-related Hb alkylation data of rats and mice exposed to 3, 10, 33, 100 or 300 (rats only) ppm EO for 6 h/day, 5 day/week, over 4 weeks, it was inferred that an external exposure to 0.027 ppm EO would lead to a level of Hb hydroxyethylation in rats which is actually found as the endogenous background (Bolt 1996; Filser et al. 1994). This is in principal agreement with more recent modelling approaches (Csanady et al. 2000; Filser and Klein 2018) that predict the valine adduct burden of long-term exposure to 1 ppm EO to be 2500 pmol/g Hb.

In humans, non-exposed individuals seem to have an endogenous body burden corresponding to around 20 pmol valine adduct/g of Hb, which is increased by tobacco smoking by a factor around 4. The contribution of the gut microbiome to endogenous exposure is estimated to be around a third, with probably larger variation, based on the gut microbiome composition. Based on the above assessments, endogenous EO exposure in the absence of external exposure thus is approximately equivalent to a workplace exposure of ≤ 0.008 ppm EO (Kirman et al. 2021).

Compared to the average background of 20 pmol hydroxyethyl valine/g Hb in non-exposed healthy non-smokers, there is a difference by almost three orders of magnitudes in the impact of endogenous vs. exogenous EO exposure, compared to the above derivation based on the DNA adduct *N*^7^-(2-hydroxyethyl)guanine. This apparent “ethylene oxide paradox” (Bolt 1996) is still unresolved. The following conclusions may be drawn at the present time:

(i) Because of methodological reasons, nearly all studies on DNA alkylation by EO are focussed on the quantitatively major adduct, *N*^7^-(2-hydroxyethyl)guanine, of which the biological impact is limited. However, there is paucity of data on more relevant DNA adducts, such as *O*^6^-(2-hydroxyethyl)guanine or *N*^3^-(2-hydroxyethyl)adenine. This is the limiting factor for comparative risk assessments based on DNA alkylation.
(ii) In humans, the Hb adduct burden (at the N-terminal valine) of long-term exposure to 1 ppm EO was calculated to be 2500 pmol/g Hb (Filser and Klein 2018), in reasonable agreement with available experimental values. Compared to the average background of 20 pmol hydroxyethylvaline/g Hb in non-exposed healthy non-smokers (Boogaard et al. 1999), this means an increase by two orders of magnitudes per inhaled ppm ethylene oxide (under workplace conditions). Therefore, Hb alkylation may serve as a particularly sensitive marker of exposure (Hartwig et al. 2020).
(iii) The “ethylene oxide paradox” suggests that endogenous formation of *N*^7^-(2-hydroxyethyl) guanine may largely derive from sources other than EO, the nature of which is presently unclear.

### Furans

#### Characterisation, formation, occurrence and public health concern

**Fig. 12:**
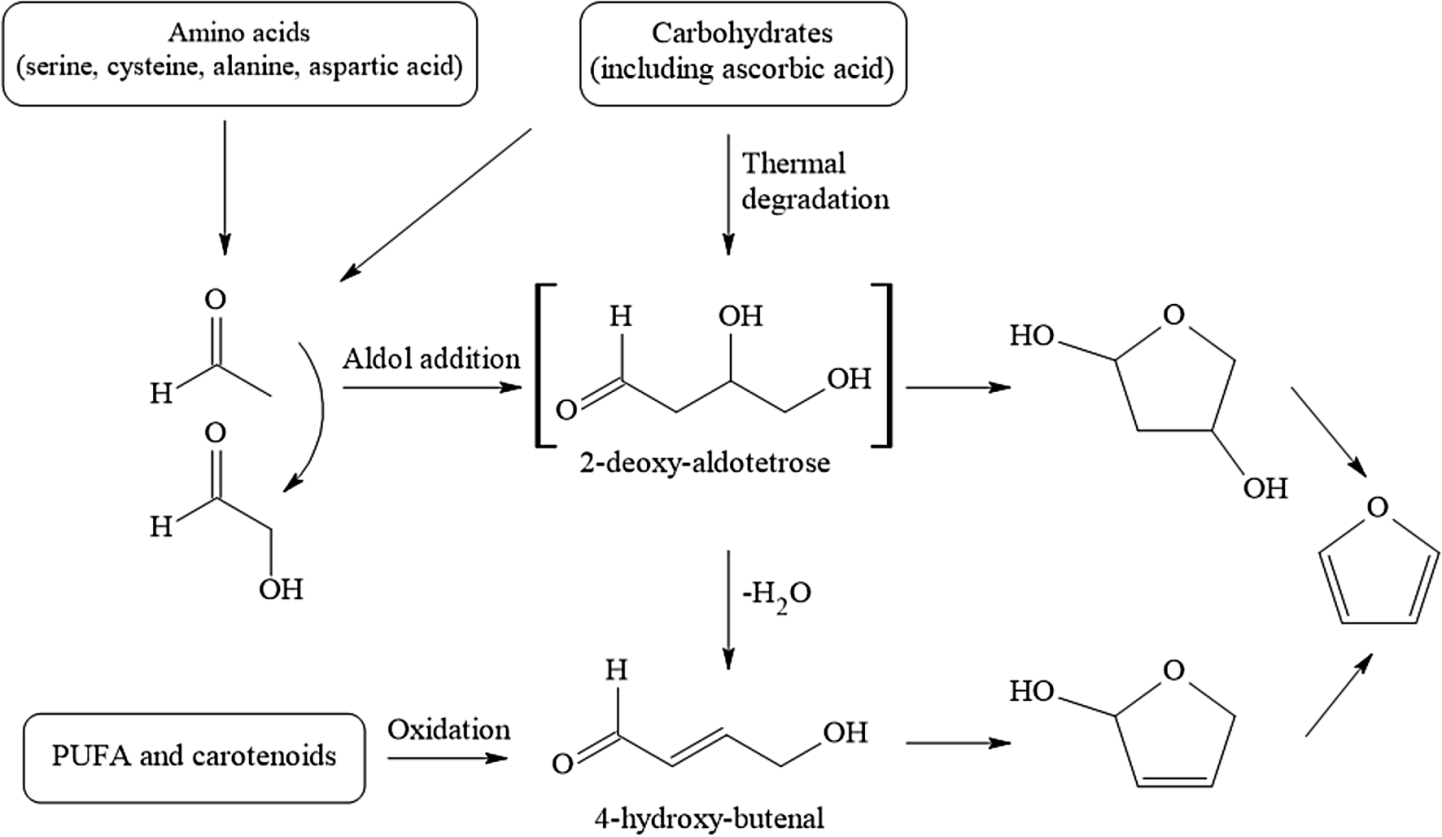
Proposed formation pathways from the most important precursors of furan (Adapted from Yaylayan 2006)

The organic, heterocyclic compound furan is a colourless, highly volatile liquid which represents an important intermediate in the industrial production of chemicals and pharmaceuticals and is also widely used in the production process of lacquers, stabilisers, resins, and insecticides. Furan is released into the environment as a product of incomplete combustion through exhaust gases from diesel and gasoline engines, through various combustion processes (e.g. waste, wood) and through industrial effluents (IARC 1995). During the processing of foods, furan can be formed under the influence of heat. Several naturally occurring food components such as ascorbic acid, polyunsaturated fatty acids (PUFAs), sugars, amino acids and carotenoids serve as precursors. Different formation pathways have been investigated and specific mechanisms were proposed (Fig. 12). Furan can be formed from PUFAs via lipid peroxidation. This pathway involves the formation of different reactive aldehydes, e.g. 4-hydroxy-2-butenal by enzymatic (lipoxygenase) or non-enzymatic (reactive oxygen species) mechanisms. Subsequent cyclisation and dehydration produce furan derivatives (Perez Locas and Yaylayan 2004). The key step in the formation of furan from amino acids is the formation of acetaldehyde and glycolaldehyde. In an aldol condensation, these degradation products can form 2-deoxyaldotetrose, from which furan is formed through cyclisation and dehydration reactions. While thermal degradation of serine and cysteine results in the formation of acetaldehyde and glycolaldehyde, the amino acids aspartic acid, threonine and alanine give rise only to acetaldehyde and thus the presence of sugars as an external source of glycolaldehyde is required for furan formation. The proposed mechanism of furan formation from the precursor ascorbic acid also involves 2-deoxyaldotetrose as an important intermediate. Via the Maillard reaction, sugars can generate furan from the intact sugar skeleton or from recombined sugar fragments (Limacher et al. 2008).

The formation of furan in food strongly depends on reaction conditions such as temperature and pH value. The pH value for efficient furan formation differs for each precursor. While sugars produce higher amounts of furan under neutral conditions, formation of furan from ascorbic acid preferentially occurs in an acidic milieu. Dry heating conditions usually lead to higher furan concentrations than wet heating conditions (Limacher et al. 2007). The highest furan values are obtained by a quick heating process at high temperatures (Altaki et al. 2011; Limacher et al. 2007).

Since 2004, EFSA has evaluated occurrence data on furan levels of numerous samples of different food categories. Coffee showed the highest furan content, which correlates with the degree of roasting and also depends on the preparation type. High furan concentrations are achieved using a fully automated coffee machine or cartridge coffee, while instant coffee contains only small amounts of furan (Kettlitz et al. 2019; Rahn and Yeretzian 2019). However, due to its high volatility, the content of furan decreases in the course of coffee preparation from roasting to consumption of the final beverage, resulting in a total loss of about 90% (Guenther et al. 2010). High furan levels were also found in canned or jarred baby food, as it is formed and sealed in the container during temperature treatment. EFSA (2011) reported a mean content of 31–32 µg/kg and a maximum of 233 µg/kg furan in processed baby food. The highest mean content is found in ready-to-eat meals consisting of vegetables only (48–49 µg/kg) and meat-based meals (40 µg/kg), while the lowest values were found in baby food containing only fruit (2.5–5.3 µg/kg). Furan could not be detected in freshly home-prepared food (Lachenmeier et al. 2009). Other foods identified as sources of exogenous exposure with maximal furan contents above 100 µg/kg are cereals, meat and dairy products, soups and sauces. Due to its high volatility, furan can also evaporate from food, so that individual preparation methods such as reheating, stirring or storage conditions affect the final concentration (Knutsen et al. 2017). The retention of furan in food is also influenced by certain matrix components. One study investigated the effect of oils and starch on the retention of furan in food and showed that especially fats are able to decrease the evaporation of furan (Van Lancker et al. 2009).

After oral intake, at least 80% of the furan dose is rapidly absorbed in the gastrointestinal tract. Elimination takes place via metabolites found in urine and faeces as well as via exhaled air as unchanged furan and CO_2_ (Burka et al. 1991). Furan is predominantly metabolised by cytochrome P450 CYP2E1 (Kedderis et al. 1993). CYP2E1 mediated biotransformation of furan results in the formation of *cis*-2-butene-1,4-dial (BDA), a highly reactive electrophile, which is able to form covalent adducts with cellular nucleophiles such as proteins, amino acids or DNA bases (Chen et al. 1995). The liver is the main target organ of furan toxicity. While furan is a strong hepatotoxin in rats and mice after oral administration, its nephrotoxic effect is less pronounced. It is also a carcinogen in rodents and was classified as possibly carcinogenic in humans (Group 2B) by IARC (1995). Carcinogenicity studies in rodents showed the occurrence of hepatocellular adenomas and carcinomas in rats and mice and the occurrence of cholangiocarcinomas in rats (Moser et al. 2009; NTP 1993; Von Tungeln et al. 2017). The mechanism of furan carcinogenicity is not fully understood. Studies on furan genotoxicity provide contradictory results. Furan was not mutagenic in bacteria. In vitro studies showed that furan induces sister chromatid exchanges and chromosomal aberrations, but no DNA strand breaks. However, furan was able to cause chromosomal aberrations and DNA strand breaks in livers of rats and mice in vivo at higher doses. In contrast to furan, its reactive metabolite BDA was positive in the Ames test using *Salmonella typhimurium* TA104 and induced DNA strand breaks and crosslinks in CHO cells (Moro et al. 2012). The potential genotoxic mechanism of furan carcinogenesis led to the decision of EFSA to use the MOE approach for risk assessment instead of deriving a TDI. The BMDL_10_ was chosen as the point of departure (PoD) and applied for the non-neoplastic endpoint cholangiofibrosis (0.064 mg/kg bw) as well as the combined neoplastic endpoint hepatocellular adenoma and carcinoma (1.31 mg/kg bw) (EFSA 2017). The MOEs for the incidence of non-neoplastic effects were smaller than 100 for some exposure estimates, especially for the high percentile exposure estimates for infants, toddlers and adults and thus indicate a health concern for these consumer groups. In terms of neoplastic effects, most MOE values calculated from the mean dietary exposure and all MOE values calculated from the 95th dietary exposure were below 10,000, indicating a health concern from the potentially genotoxic carcinogen furan. However, there is uncertainty regarding the carcinogenic mode of action of furan. The CONTAM Panel concluded that the current chronic dietary exposure to furan is a risk to human health (EFSA 2017). The Panel noted that methylfurans may add to the overall exposure and risk of hepatotoxicity. While the uncertainties on the risk assessment of furan were overall classified as ‘moderate’, the CONTAM Panel considered the uncertainties for methylfurans as ‘large’ due to the limited data available, so that a full risk assessment of the sum of furan and methylfurans was not possible (Knutsen et al. 2017).

#### Existing knowledge on exogenous sources

##### Exogenous exposure from food

Human exposure is mainly caused by diet as furan is present in a wide range of heat-treated foods. An estimation of human exposure to furan via food based on consumption data from 35 dietary surveys from 19 European countries was published by EFSA (2017). The calculated dietary exposures show that the population group ‘infants’ is the most exposed to furan. Their estimated mean exposure ranged from 0.14 to 0.99 µg/kg bw/day (minimum LB to maximum UB) and is mainly due to the consumption of ready-to-eat meals. Besides the subpopulation infants, exposure to furan via ready-to-eat meals is only relevant for toddlers, but to a lesser extent. The mean dietary exposure of toddlers is 0.22–0.65 µg/kg bw/day and is mainly caused by the consumption of grains and grain-based products. This food category is also the main contributor for children (≥ 36 months to < 10 years) and adolescents and the second largest exogenous source of furan for all other age groups. Comparing the consumer groups, adolescents show the lowest dietary exposure to furan with a mean exposure of 0.11–0.31 µg/kg bw/day. In this age group, only 12% of the exposure is driven by non-alcoholic beverages such as coffee. In contrast, coffee accounts for up to 85% of the exposure of adults, elderly and very elderly and is thus the main contributor in these age groups. The mean dietary exposure of these consumers ranges from 0.11 to 0.75 µg/kg bw/day (Knutsen et al. 2017).

##### Exogenous exposure from other sources

Exposure to furan from cigarette smoke is one of the most important exogenous sources besides dietary intake. The FDA added furan to its list of ‘Harmful and Potentially Harmful Constituents in Tobacco Products and Tobacco Smoke’ and has defined furan as a carcinogen (FDA 2012). The content of furan in cigarette smoke has been analysed by different methods. Amounts from 20.2 to 37.3 µg of furan/cigarette were reported (Hatzinikolaou et al. 2006; Pouli et al. 2003). The consumption of waterpipe tobacco is also an exogenous source of furan exposure (Kassem et al. 2020).

Since furan can evaporate from food during cooking, Crews (2009) determined the concentration of furan in kitchen air after different cooking times and estimated the amount of inhaled furan. Data show that furan levels in kitchen air depend on the food product, cooking technique and duration. The highest amount of furan is inhaled during the preparation of coffee in cafetiere and frying or baking of chipped potatoes (Crews 2009). Prasse et al. (2018) studied the formation of α,β-unsaturated enedials and oxoenals such as BDA from phenolic contaminants in drinking water sources through the use of hydroxy radicals and UV light in water treatment systems and thus discovered another exogenous source not for exposure to furan itself, but to its reactive metabolite BDA (Prasse et al. 2018).

#### Existing knowledge on endogenous sources

**Fig. 13:**
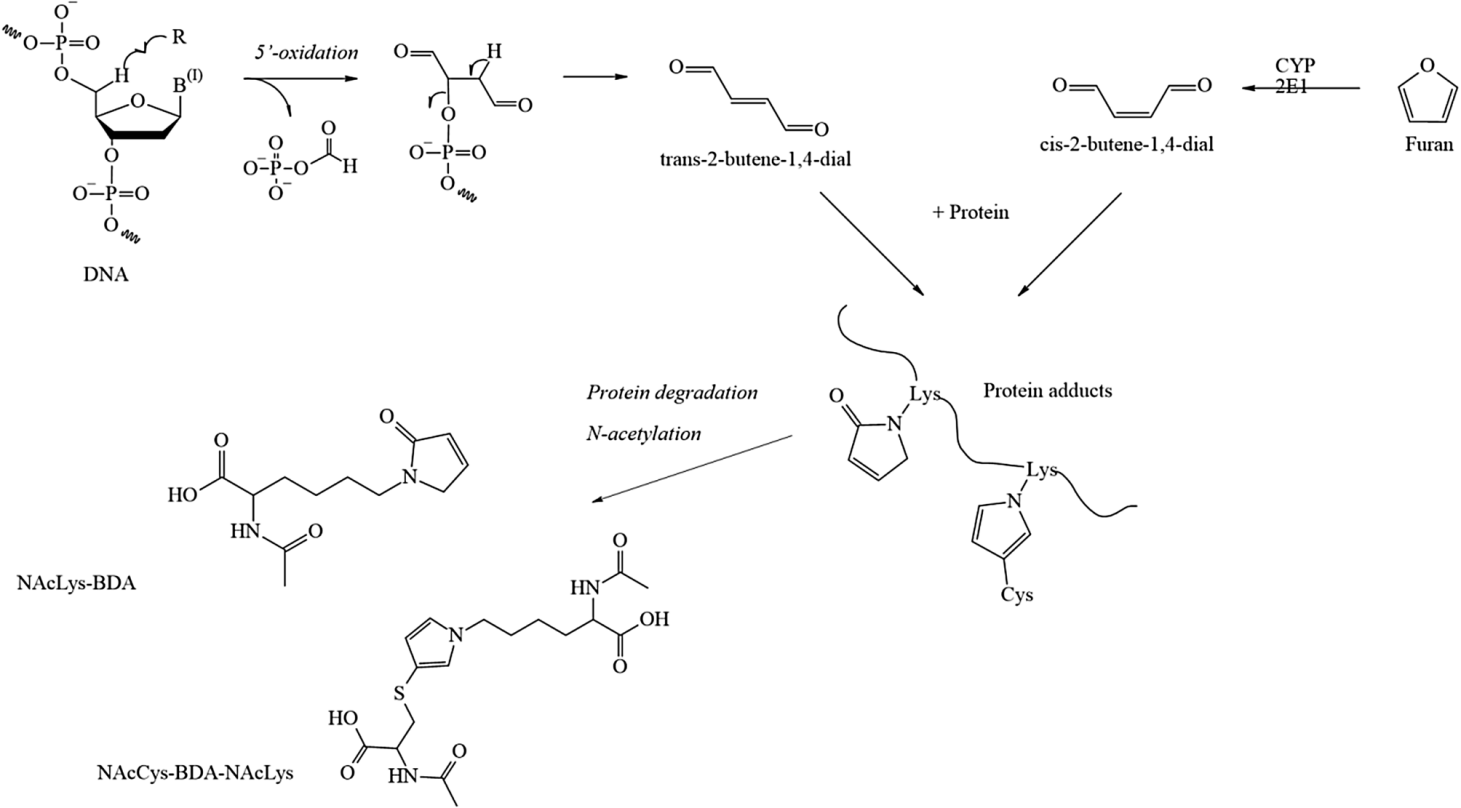
Possible endogenous formation pathway and formation from exogenous exposure of the protein-lysine-derived furan metabolites NAcLys-BDA and NAcCys-BDA-NAcLys (Adapted from Chen et al. 2004; Karlstetter and Mally 2020)

Furan and furan-derived metabolites have been detected in tissues and body fluids of untreated experimental animals, suggesting either endogenous formation or background exposure via animal feed. In searching for biomarkers of furan exposure in male F344 rats orally treated with furan at a dose of 40 mg/kg bw, Keller et al. identified the furan-dependent metabolites *R*-2-acetylamino-6-(2,5-dihydro-2-oxo-1H-pyrrol-1-yl)-1-hexanoic acid (NAcLys-BDA), *N*-acetyl-*S*-(1-(5-acetylamino-5-carboxypentyl)-1H-pyrrol-3-yl)-l-cysteine (NAcCys-BDA-NAcLys) and an as yet unidentified compound in the urine of control animals (Kellert et al. 2008). The ratio of metabolite concentrations in urine of treated relative to untreated animals varied between metabolites, and thus animal feed was considered unlikely to present the source of the background levels. If small amounts of furan in animal feed would contribute to the total furan load of the animals, the authors argued that the three metabolites detected would be expected to show a similar ratio between treated and untreated controls (Kellert et al. 2008). Endogenous occurrence of NAcLys-BDA is also supported by others who detected and quantified this metabolite in urine of untreated rats (Karlstetter and Mally 2020). A possible alternative endogenous source of furan-derived metabolites is the formation of *trans*-2-butene-1,4-dial from 5′-(2-phosphoryl-1,4-dioxobutane), which results from 5′-oxidation of deoxyribose in DNA (Fig. 13). Evidence for the formation of *trans*-2-butene-1,4-dial as a β-elimination product of the 5′-(2-phosphoryl-1,4-dioxobutane) residue in oxidised DNA was provided by two analytical approaches that indirectly detected *trans*-2-butene-1,4-dial as a pyridazine or dioxime derivative. The formation of *trans*-2-butene-1,4-dial was induced by five different oxidising agents, leading to the assumption that this lesion is a general product of DNA oxidation (Chen et al. 2004; Gingipalli and Dedon 2001). Previous studies suggest that lysine adducts are mainly formed by a reaction of *trans*-2-butene-1,4-dial with protein-bound lysine. It is, therefore, assumed that the endogenously formed *trans*-2-butene-1,4-dial reacts with lysine residues of histone proteins, resulting in the presence of lysine-dependent metabolites in the urine of control animals (Fig. 13) (Karlstetter and Mally 2020). A study investigated whether the presence of furan in blood or liver of experimental animals can be caused by possible background contamination in animal feed using a sensitive headspace solid phase microextraction GC method (Becalski et al. 2013). F344 rats were given deuterated furan at a dose of 120 ng/kg bw similar to the presumed exposure via typical laboratory animal feed. While unlabelled furan was present at concentrations of 0.09–0.18 ng/g in blood and 0.04–0.12 ng/g in liver tissue, no measurable levels of labelled furan could be detected in blood and liver of animals treated with d_4_-furan. Since the furan levels in animal feed had no influence on the tissue concentrations, endogenous furan formation was suspected (Becalski et al. 2013).

#### Available biomarkers (furans)

The high reactivity of *cis*-2-butene-1,4-dial (BDA) towards proteins and DNA gives rise to a broad spectrum of furan-dependent metabolites which present potential biomarkers of furan exposure. The association between urinary excretion of such metabolites and oral exposure to furan has been investigated in several in vivo studies. The presence of the cyclic mono-GSH-BDA conjugate in urine of male F344 rats after a single dose of 8 mg/kg bw furan shows that BDA is trapped in vivo by GSH and indicates the potential use of the GSH-BDA adduct as a marker of furan intake (Peterson et al. 2006). After oral administration of 40 mg/kg bw furan, 13 potential biomarkers that increased in the urine of male F344 rats were identified using a combination of a mass spectrometric analytical procedure and multivariate analysis (Kellert et al. 2008). Five structures, all containing a 3-methylthio-pyrrole, were identified as conjugates of BDA with GSH, or *N*-acetylated lysine and as cysteine-BDA-lysine crosslinks (Kellert et al. 2008). A further mass spectrometric analysis of rat urine confirmed the formation of the BDA-derived metabolites mono GSH-BDA, NAcLys-BDA, NAcCys-BDA-NAcLys and NAcCys-BDA-NAcLys sulfoxide related to furan treatment (Lu et al. 2009). The metabolites NAcLys-BDA and mono GSH-BDA were further examined as putative biomarkers of furan exposure. A quantitative LC–MS/MS analysis in urine of rats receiving furan doses ranging from 0.1 to 2 mg/kg bw for 5 and 28 days demonstrated a linear relationship between urinary excretion of these metabolites and external furan dose (Karlstetter and Mally 2020). These results support the suitability of a biomarker-based approach for furan exposure assessment. Human biomonitoring of dietary exposure has not yet been carried out. However, a strong correlation was demonstrated between smoking and the excretion of NAcCys-BDA-Lys sulfoxide which was significantly increased in the urine of smokers from three different cohorts and decreased following cessation of smoking (Grill et al. 2015). Similarly, analysis of urinary furan metabolites in waterpipe tobacco smokers revealed a significant increase in the two metabolites NAcCys-BDA-Lys sulfoxide and NAcCys-BDA-NAcLys in waterpipe tobacco smokers compared to non-smokers (Kassem et al. 2020). While metabolic profiling of rat urine revealed some promising biomarkers of furan exposure, there is no indication that DNA adducts may present suitable biomarkers for furan exposure. Neuwirth et al. discovered a dose-dependent increase in radiocarbon content in liver DNA of rats treated with (3,4-^14^C)-furan, but were unable to structurally characterise these DNA modifications (Neuwirth et al. 2012). Investigations of histone modifications seem to offer new possibilities for furan biomarkers as a furan-derived adduct of GSH-BDA and histone H2B was extracted from liver of furan-treated rats (Nunes et al. 2016).

### 3-MCPD (3-monochloropropane-1,2-diol) and 2-MCPD (2-chloro-1,3-propanediol)

**Fig. 14:**
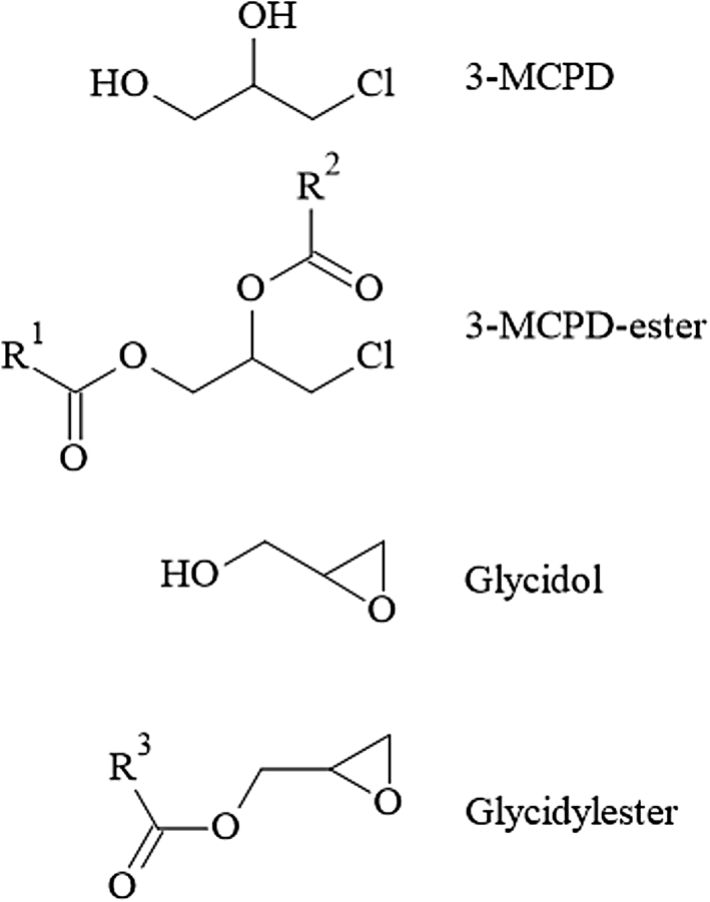
Chemical structure of 3-MCPD, 3-MCPD-esters, glycidol and glycidylesters

3-MCPD (3-monochloropropane-1,2-diol) and 2-MCPD (2-chloro-1,3-propanediol) are derivatives of glycerol, where one hydroxyl group is replaced by chlorine. As process contaminants, also 3-MCPD- and 2-MCPD-esters are relevant. 3-MCPD- and 2-MCPD-esters are derivatives of triacylglycerides, which are the predominant compounds in vegetable and animal fats. Normally three fatty acids are esterified to glycerol to build up a lipid molecule. In MCPD molecules one hydroxyl group of the glycerol backbone is replaced by chlorine, either in the middle position (2-MCPD) or in one of the outer positions (3-MCPD) (Fig. 14). Due to the variety of fatty acids occurring in vegetable and animal fats, a number of different 3-MCPD-esters can be present as process contaminants in oils and fats (Haines et al. 2011).

#### Characterisation, formation, occurrence and public health concern

Free 3-MCPD has been described as a food contaminant already in the early 1980s (Velisek et al. 1980). There is clear evidence that it is carcinogenic in male rats, with some evidence in female rates (Cho et al. 2008). In 2011, 3-MCPD was categorised by IARC as a ‘‘possible human carcinogen’’—Category 2B (Grosse et al. 2011). Still there are also publications indicating that 3-MCPD shows no tumour generating activity (Jeong et al. 2010). According to the contradictory findings of the researchers, Abraham et al. proposed to consider the carcinogenic potential of free 3-MCPD as species specific (Abraham et al. 2013). Besides the carcinogenic properties of 3-MCPD, Kirton et al. described infertility inducing effects (Kirton et al. 1970).

The TDIs for 3-MCPD have been the subject of several discussions and have been changed several times. In early 2016, EFSA published a TDI of 0.8 µg/kg bw/day. Only 6 months later, JEFCA published a TDI of 4 µg/kg bw/day. The reason for the divergent TDI values in both studies was mostly the utilisation of different mathematical models applied to the same database. The latest revised opinion from EFSA resulted in a TDI of 2 µg/kg bw/day (EFSA 2018).

Since free 3-MCPD and 3-MCPD esters share the 3-MCPD structure, toxicological properties of 3-MCPD esters likely include those of free 3-MCPD. The esters of 3-MCPD are rapidly and almost completely hydrolysed after absorption liberating 3-MCPD (Abraham et al. 2013). Accordingly, toxicological properties of free 3-MCPD largely apply to 3-MCPD esters as well.

2-MCPD has a structure very similar to 3-MCPD. An oral toxicity study with 2-MCPD, 3-MCPD and 2-MCPD dipalmitate in Wistar rats and subsequent bioinformatic analysis of the proteome data indicated the toxicology of both compounds to be markedly different (Schultrich et al. 2017). However, detailed toxicological data or even an estimation of a TDI for 2-MCPD are still missing.

Several risk assessments related to exposure of the population in Europe to 3-MCPD have been carried out, including recent ones from EFSA (2018) and BfR (2020) as well as earlier, unpublished assessments conducted by industrial food producers or suppliers. From these assessments also different recommendation for TDI levels (see above) ranging from 0.8 to 4 µg/kg bw/day were derived. The EFSA TDI of 2 µg/kg bw/day (EFSA 2018) has been applied to derive the currently valid legal limits in the EU for 3-MCPD and 3-MCPD esters.

In its evaluation, BfR (2020) concluded that—even though increased health risks for adults are not expected—there is still a risk for infants and a need for further research. Especially data on the toxicity of 2-MCPD and its fatty acid esters is very limited and, accordingly, no conclusive assessment of its potential health risks is currently possible.

Given that 3-MCPD esters and 2-MCPD esters emerge from the same processes of vegetable oil refining, it is unsatisfactory that current risk assessments only focus on 3-MCPD- and glycidyl esters. Moreover, exclusive exposure through food has been considered, while exposure from other sources has not yet been taken into account.

#### Existing knowledge on exogenous sources

##### Exogenous exposure from food

Free 3-MCPD occurs in various food types such as bakery goods (Hamlet et al. 2004), malt-derived foods, coffee (Doležal et al. 2005), cheese and meat (Crews et al. 2001), cold smoked products (Reece et al. 2005), and fish (Calta et al. 2004). Highest amounts have been found in hydrolysed vegetable protein and flavouring sauces such as soy or oyster sauce (Collier et al. 1991). There are different pathways how the contaminant is formed. Precursors can be allyl alcohols, glycerol, triglycerides, and phospholipids (each in conjunction with chlorine) or epichlorohydrin and 3-MCPD-esters releasing the free compound.

Most human 3-MCPD exposure occurs through the consumption of refined vegetable oils which can contain notable amounts of 3-MCPD esters. Since 3-MCPD esters emerge in the refining process, native oils do not contain any of those contaminants.

To refine vegetable oils a bleaching process and a deodorisation at temperatures of 180–260 °C or higher are normally applied. Under the applied conditions, organochloric compounds can split off chlorine or hydrochloric acid that react with mono- and diglycerides, which are naturally part of most vegetable oils, to 3-MCPD esters (Craft et al. 2012; Destaillats et al. 2012; Nagy et al. 2011). Under acidic conditions, all types of chlorine sources can react with mono- and diglycerides to 3-MCPD esters. Besides chloride being ubiquitously present, fertiliser components such as iron chloride or sea water may contribute as sources leading to 3-MCPD in the refining process (Destaillats et al. 2012). In particular, oils and fats that are stored for a long time and transported over long distances before being refined are susceptible to the formation of 3-MCPD (i.e. palm oil). Washing fresh palm oil to remove chloride ions can reduce the potential to build 3-MCPD (Craft et al. 2012).

A collection of publications and patents presenting means to prevent the formation of 3-MCPD in the refining process and to mitigate 3-MCPD presence in food oils has been published by the Lebensmittelverband Deutschland (2016).

In the EU, legal limits for 3-MCPD and its fatty acids esters are in place. Hydrolysed vegetable proteins and soy sauces must not exceed a level of 20 µg/kg. Coconut-, maize-, rapeseed-, sunflower-, soybean-, palm kernel and olive oils for human consumption and for the production of food are not allowed to exceed levels of 1200 µg/kg. Other oils like for instance palm oil are not allowed to exceed 2500 µg/kg. For fats and oils used in infant or baby nutrition, even lower limits apply (max. 750 µg/kg). In powdered formulation for the preparation of infant formula and foods for special medical purposes, a maximum limit of 125 µg/kg has been given, while for prepared infant formula levels of max 15 µg/kg 3-MCPD apply as laid out in Commission Regulation (EU) 2020/1322 (European Commission 2020). Legal maximum levels for 2-MCPD or 2-MCPD fatty acid esters have not been defined yet.

##### Exogenous exposure from other sources

In 2016, Becalski et al. reported that 3-MCPD can be contained in paper products and could also be transferred to food products from coffee filters, paper tissues or packaging materials (Becalski et al. 2016). Though the concentrations in this type of products are low compared to the levels found in food, particularly in vegetable oils, the frequent use of such products could also contribute to the overall 3-MCPD intake.

#### Existing knowledge on endogenous sources

Even though it was reported that 3-MCPD occurs in human breast milk (Zelinkova et al. 2008), it is assumed that 3-MCPD is not formed by biochemical processes in the human body and that 3-MCPD reaches the human milk through food uptake.

#### Available biomarkers

It has also been found that 3-MCPD oxidises to a significant amount DJ-1 proteins (Buhrke et al. 2018), mainly in kidney, but also in other body tissues and cells. The authors propose oxidised DJ-1 proteins as biomarkers for 3-MCPD exposure which needs further exploration. DJ-1 oxidation may be biologically relevant since the protein is involved, e.g. in intracellular redox regulation through interaction with the “master switch” redox regulator Nrf2, in transcriptional regulation of thioredoxin-/-GSH homoeostasis, and in co-operation with proteins maintaining mitochondrial function. Additional functions attributed to DJ-1 include acting as a chaperone for α-synuclein, preventing its misfolding and exerting protease and glyoxalase activity [(Buhrke et al. 2018) and references therein].

Earlier work has shown that 2,3-dihydroxypropyl mercapturic acid (DHPMA) and its isomers could also be used as biomarkers (Jia et al. 2019). In contrast, Abraham (Abraham et al. 2020) found no linear relation between MCPD intake and urinary DHPMA excretion. It has rather been proposed to use urinary 3-MCPD and 2-MCPD directly to measure 3-/2-MCPD exposure. Still the methodology to detect the compounds in low levels needs to be improved.

#### Impact of endogenous formation on risk assessment

Up to now, no significant endogenous formation of 2- and 3-MCPD or their esters is known. The exogenous exposure levels reported may suggest that endogenously produced 3- and 2-MCPD if shown, will only contribute marginally to the overall exposure. Of note, it has been hypothesised that glycidol may be converted in the organism to some extent to MCPD, (Scholz and Schilter 2014). Hypothetically, this may become relevant, provided endogenous sources for glycidol are uncovered, in view of the ubiquitous occurrence of physiological chloride levels in the extra- and intracellular space of the organism.

### Glycidyl esters

Glycidol and its esters (G(E)) are process-related contaminants primarily found in refined fats and oils, and foods containing fats and oils. G(E) have been previously evaluated by JEFCA (2017) and EFSA (2016).

#### Characterisation, formation, occurrence, and public health concern

GE are formed from precursors such as diacylglycerols (DAG) (Destaillats et al. 2012) and monoacylglycerols (MAG) (Cheng et al. 2017) by mechanisms involving intramolecular rearrangement through charge migration differing from each other in the nature of the intermediate (acyloxonium ion or oxonium ion) and the leaving group (free fatty acid or water) (Destaillats et al. 2012).

Glycidol and GE are efficiently absorbed following ingestion, the esters being rapidly hydrolysed into glycidol in the digestive tract. Their metabolism has been reviewed in depth by EFSA (2016) and JEFCA (2017) and was summarised by Scholz and Schilter (2014).

Glycidol was shown to be genotoxic. Toxicity studies on glycidol showed a low acute toxicity, renal toxicity, temporary/reversible infertility, immunotoxicity and neurotoxicity. In oral long-term studies in mice and rats, glycidol induced tumours in various tissues in both sexes. As lowest effective dose level, 17.9 and 26.8 mg/kg bw/day were reported for mice and rats, respectively.

The United States National Toxicology Program (NTP 1990, 2007) concluded that there was “clear evidence for carcinogenic activity”. IARC classified glycidol as probably carcinogenic to humans (Group 2A) (IARC 2000).

Both JEFCA and EFSA considered carcinogenesis as the pivotal outcome for risk assessment (EFSA 2016; JEFCA 2017). JEFCA calculated as toxicological reference value a BMDL10 of 2.4 mg/kg bw/day. EFSA considered that a BMDL10 cannot be derived because of inadequacy of the data for modelling and set a T25 dose (25% increase in incidence of a specific tumour above background incidence in the lifespan of the species) of 10.2 mg/kg bw/day.

#### Existing knowledge on exogenous sources

##### Exogenous exposure from food

Using different dataset and food consumption surveys, EFSA (2016) and JEFCA (2017) found comparable dietary intakes for G(E) (Kemeny et al. 2019). For all age classes except infants, G(E) intakes ranged from 0.1 to 2.1 μg/kg bw/day. In both evaluations, results on infants exclusively consuming infant formula showed higher intake estimates for glycidol (up to 4.9 μg/kg bw/day). These findings are of the same order as reported by BfR (2020).

MOEs were calculated by dividing toxicological reference values by average exposure levels. Even considering different toxicological reference values and different ways of dietary intake estimation, EFSA, JEFCA and the German BfR reached similar conclusions for G(E). MOEs were deemed insufficient (< 10,000) mainly for high consumers and particularly for infants receiving formula only and, therefore, pointed towards a health concern. EFSA and JEFCA agreed that the dataset of levels of G(E) in foods should be completed and updated to refine dietary exposure calculations to also take into account current mitigation achievements and to quantify separately free and ester forms.

##### Exogenous exposure from other sources

Preliminary observations in consumers who exclusively consumed foods not heated at temperatures over 42 °C (‘raw food eaters’), indicated a background blood level of glycidol Hb adducts. It was speculated that exposure to glycidol from other sources, to glycidol from endogenous origin or exposure to an as yet unknown C3 compound may be causative (BfR 2020).

Some studies show that smoking contributes to glycidol exposure in humans: higher levels of *N*-(2,3-dihydroxypropyl)-valine (2,3-diHOPr-Val) were detected in blood samples of smokers (Aasa et al. 2019; Landin et al. 1996; Monien et al. 2020). The probable source of glycidol in cigarette smoke is glycerol used as additive in tobacco (Carmines and Gaworski 2005). Glycidol was shown to be present in e-cigarette vapour probably formed by dehydration of the glycerol component of the liquid at high temperatures (Laino et al. 2011; Sleiman et al. 2016).

#### Existing knowledge on endogenous sources

Following a literature search, no clear evidence of endogenous formation of G(E) was found. EFSA and JEFCA reported that the microbial enzyme halohydrin dehalogenase is able to dehalogenate haloalcohols to produce glycidol (EFSA 2016; JEFCA 2002). However, this was not demonstrated in bacterial strains present in the human gut microbiome and, moreover, conversion of orally administered 3-MCPD or it esters into glycidol was not observed in rats during metabolism studies.

Glycidol-related substances in foodstuffs (including anhydrosugars, allyl alcohol, and glycerol halohydrins), have been discussed as possible precursors of glycidol (Rietjens et al. 2018).

#### Available biomarkers

**Fig. 15:**
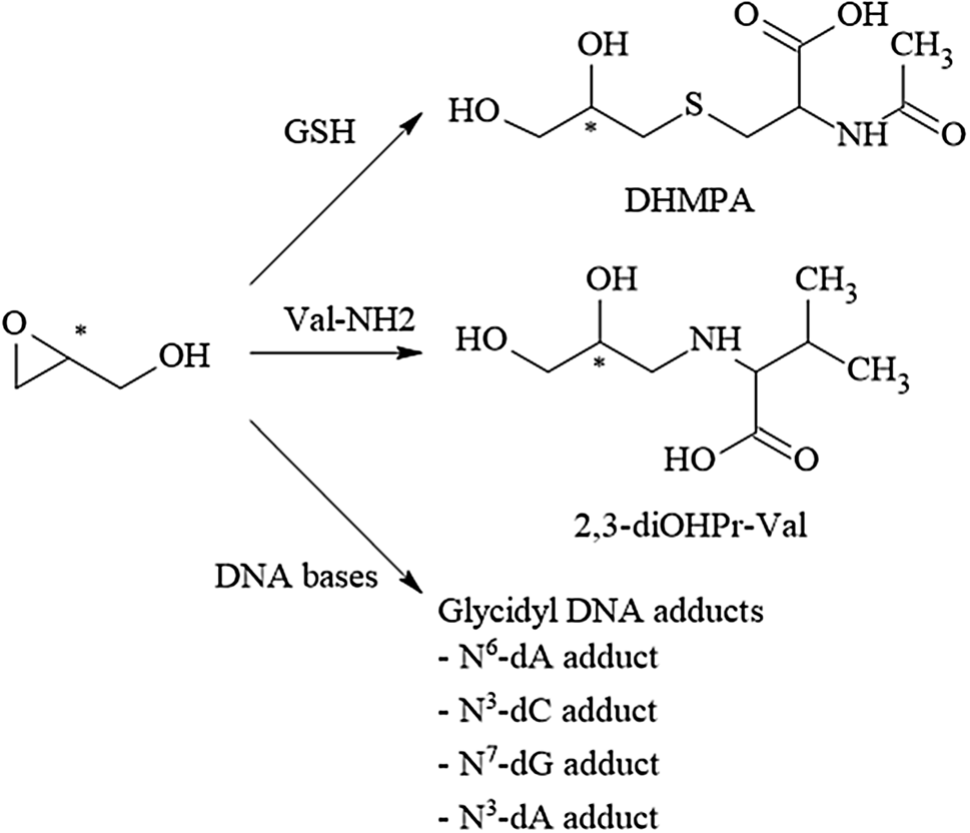
Biomarkers of glycidol/glycidylester exposure including 2,3-dihydroxypropylmercapturic acid (DHPMA) and *N*-(2,3-dihydroxypropyl)valine (2,3-diOHPr-Val) and several DNA adducts. *dA* deoxyadenosine, *dC* deoxycytidine, *dG* deoxyguanosine (Adapted from Rietjens et al. 2018)

A full review of GE biomarkers was performed previously (Rietjens et al. 2018). The authors discussed urinary, blood and tissues biomarkers (Fig. 15). DHPMA in urine, 2,3-diHOPr-Val adducts on Hb and glycidyl-DNA adducts were identified as promising exposure biomarkers.

In a controlled exposure study with 11 volunteers, (Abraham et al. 2019) it was shown that daily consumption of palm oil with a high content of glycidyl esters for 4 weeks led to an increase in 2,3-diHOPr-Val levels in Hb during the intervention period. The authors also calculated mean daily glycidol exposure supposedly related to the adduct levels of the volunteers and arrived at exposure levels substantially higher as to be expected from average intake levels reported by EFSA (Abraham et al. 2019; EFSA 2016). As possible explanations for these differences, lack of data for food not covered in the EFSA database, formation of glycidol during home-cooking, passive inhalation exposure (e.g. from cigarette smoke) and endogenous formation were discussed. It also was concluded that currently there are no indications that substances other than glycidol may form the adduct 2,3-diHOPr-Val in a relevant amount (Abraham et al. 2019).

#### Impact of endogenous formation on risk assessment

Up to now, no compelling evidence of endogenous formation of glycidol or its esters was demonstrated. From a risk assessment perspective, endogenous formation, if any, currently is considered insignificant (Abraham et al. 2019).

## Discussion and conclusion

From the overview presented above, it becomes clear that endogenous formation of several PRCs and related compounds is a fact. Moreover, for some compounds, such as ethanol, acetaldehyde, formaldehyde and acrolein, substantial endogenous formation implies that for people not being exposed to a noteworthy extent from exogenous sources such as food, cigarette smoke or the occupational setting, the endogenous exposure may account for a major part of the overall exposome.

For the design of meaningful epidemiological studies on potential associations between dietary PRC exposure and human health, beyond the issue of endogenous background, further potential confounding needs to be considered. It is widely accepted that epidemiological studies need to be supported by adequate biomarker dosimetry to achieve meaningful outcomes. This is especially true when attempting to find out correlations with exposure to specific compounds. Food usually contains a whole spectrum of PRCs, most probably at varying concentration ratios, depending on the processes it has undergone. This makes attempts highly questionable to establish an association between diet-related exposure to individual PRCs and human health outcomes. Moreover, human endogenous exposure can be expected to markedly impact results, depending on whether it is exceeding, close to, or lower than dietary exposure. Even more importantly, it is subject to interindividual physiological variability, comprising variables such as composition of human microbiomes, dependence on nutritional and health status, gender, age, lifestyle, and socioeconomic factors. Taken together all these variables may contribute to obscure potential associations with dietary exposure, especially to single agents, if not adequately addressed.

It is sometimes not entirely straightforward to draw a line between endogenous and exogenous sources of exposure. For instance, while dietary intake of glycation compounds present in heat-treated food without doubt constitutes an exogenous source, some may argue that high intake of dietary sugars as precursors for endogenous formation of glycation compounds, may also present an exogenous source. Similarly, nitroso compounds may form from exogenous exposure to nitrate/nitrite, but also endogenously from amino compounds, potentially from the diet, i.e. an exogenous source. However, such precursors like amino acids are synthesised as well in the organism. In the case of arginine, from which nitrogen monoxide is generated, it as well provides endogenously nitrogen monoxide from which nitrite, nitrate and the NOx equivalents required for in vivo formation of NOC may be formed. In the context of this work, we consider endogenous generation of PRCs from nutrients and other molecular constituents of cells required for energy metabolism, cell growth and function (e.g. carbohydrates, proteins, lipids, nucleic acids, and hormones) an endogenous source (e.g. glycation compounds formed endogenously from sugars, nitroso compounds formed endogenously from amines/amino acids, α,β-unsaturated alkenals formed endogenously by lipid peroxidation), whilst formation of PRCs during xenobiotic metabolism (e.g. acetaldehyde formed from exogenous ethanol, nitroso compounds from exogenous nitrite/nitrate/NOx) is considered exogenous.

However, often endogenous formation is not yet adequately quantified. Only for a few compounds such quantitative information is already available to reliably inform about the ratio of endogenous versus exogenous exposure levels. Yet, dependable information on endogenous exposure is considered a mandatory element of a comprehensive risk assessment and has already been taken into consideration in a few cases in the past. For example, the exposure to ethanol resulting from use of ethyl esters as flavour constituents does not add substantially to the overall exposure and thus does not raise a safety concern (JEFCA 1970). For some compounds, the existence of a substantial endogenous exposure may result in what could be a so-called “practical threshold” where low dose exposure levels are negligible, compared to the endogenous exposure levels. This was illustrated by the data presented for acetaldehyde and formaldehyde, where the use of exogenous labelled compounds allowed comparison to unlabelled endogenous levels, revealing that at low dose the exogenous exposure did not substantially increase the overall exposure. Another possibility is to use strictly controlled dietary uptake dosimetry, as achieved by duplicate diet measurement for AA intake, in connection with biomarker-based total exposure monitoring.

Of all compounds discussed in this work, formaldehyde is probably one of the most thoroughly investigated compounds, with respect to the comparison between endogenous and exogenous exposure, and repeated risk assessments have been performed, both for exposure via food and via inhalation at the workplace taking the endogenous formation into account. Currently, there are refinements on the input data on the respective modelling approaches, including intracellular formaldehyde concentrations, which will give rise to even more precise predictions of the exogenous impact on DNA adduct formation and thus mutagenicity and carcinogenicity.

Ethanol provides another example where data are available to quantify endogenous exposure, as well as exogenous exposure from different sources. For example, risk assessment may compare exposure to exogenous sources in the occupational setting or from consumption of alcoholic beverages, to endogenous exposure. This entails the consequence that, even though ethanol is to be considered a genotoxic carcinogen (IARC 2018), certain exposures may not raise a concern because they can be considered negligible compared to the endogenous exposure. Ethanol is also an example that shows that the exposome includes various exogenous and endogenous exposures, providing a proof of principle that adequate assessment of exposure and risk should take the overall exposome into account. To further enable this approach, refined quantification of endogenous levels of ethanol is needed, taking into account interindividual differences concerning absorption, distribution, metabolism and excretion, together with the potential influence of dietary habits and variant health status. This concerns also polymorphisms within the ALDH2 gene, as outlined in more detail for acetaldehyde. Given that intestinal microbial metabolism of dietary carbohydrates contributes to the endogenous levels, it is likely that dietary factors will affect interindividual differences in endogenous levels of ethanol. This also applies most probably to other PRCs, as it has been reported for acrolein, acrylamide, and glycation products. It is conceivable that such interindividual differences at least in part may reflect differences in their metabolomes.

In addition to such interindividual differences, differences between target tissue sensitivity might have to be considered as was illustrated by the example of EO for which a different tissue selectivity was observed in different animal models. The lung appeared to be a particularly sensitive organ in the mouse, while brain is more susceptible to EO-induced carcinogenesis in rats. This tissue selectivity is not necessarily expected because EO does not require metabolic activation and should easily penetrate into all tissues, even the poorly perfused ones. One possible reason could be species differences in the tissue-specific GSH depletion, which, however, would probably be an irrelevant high dose effect. Another, more important mechanism could be cell-type-specific efficacy of DNA repair mechanisms, such as provided by the activity of *O*^*6*^-methyl guanosine methyltransferase (Hartwig et al. 2020).

The examples of formaldehyde and ethanol illustrate that due consideration of endogenous exposure in risk assessment also supports moving away from a hazard-based approach, where even minute exposure to a genotoxic carcinogen is considered to increase the risk of tumour formation to an extent that justifies significant measures. In contrast, a risk-based evaluation that takes endogenous exposure also into account opens the way to define acceptable exposure levels, e.g. by reference to the human endogenous background. A similar approach was chosen by the MAK Commission, who classified ethanol and its metabolite acetaldehyde in carcinogen Category 5, which defines substances that cause or are considered to cause cancer in humans or animals for which, however, a MAK value can be derived. A genotoxic mode of action is assumed to be underlying but is considered to not contribute in a noteworthy way to human cancer risk, provided the MAK and BAT values are observed (DFG 2020; Nakamura et al. 2014).

Likewise, EFSA estimated the relative contribution of formaldehyde from food versus the endogenous production and concluded that less than 1% of the estimated overall exposure from all sources originated from exogenous exposure (EFSA 2014).

Thus, taking endogenous formation into due account paves the way to a more holistic risk assessment that encompasses human endogenous background exposure where this appears to provide an important contribution to the exposome. As a consequence, analysing the total exposome and the respective contributions of endogenous and exogenous origin will refine risk assessment. Therefore, adequate exploration of the endogenous exposome is conceived to become a mandatory element that informs human health risk assessment. Where applicable, it may also serve as dosimetry-based reference point (point of departure) against which to evaluate exogenous exposure in human health risk assessment.

However, before the endogenous formation can be taken into account in risk assessment, important knowledge gaps for several compounds discussed in this review must be filled. Dependable probabilistic estimates of endogenous versus exogenous exposure are required and this remains a topic for further research. Endogenous exposure may be governed by largely unexplored factors of influence such as the composition of human microbiomes and/or the individual nutritional and health status, gender, age, lifestyle, and socioeconomic factors. Such variables appear important to explore to inform about the variance of endogenous exposure on a population level. Thus, experimental as well as human studies are needed to explore interindividual and interspecies differences. With appropriate biomarker methodology, extended and tightly controlled human nutritional intervention studies, such as those achieved to determine human endogenous exposure to AA and/or acrolein (Goempel et al. 2017; Goerke et al. 2019; Ruenz et al. 2016, 2019) are required to provide population-based data on variance and confidence intervals. Once established, this database may serve as reference to derive margins of exogenous exposure in relation to endogenous exposure. Such a novel margin of exposure will add a dependable, dosimetry-based element relevant to a future holistic risk assessment.

Similarly, for many of the thermally induced α,β-alkenals, gaps in the toxicological database required for a comprehensive risk assessment of exogenous and endogenous exposure exist. At present, an accurate assessment of exogenous exposure is difficult, given that levels of α,β-alkenals in heated oils and fats reported by different authors vary substantially. Furthermore, the actual levels of thermally induced aldehydes in oils and fats depend on the type of oil, temperature and process duration. Manufacturing processes may change to reduce the risk of thermally induced aldehyde formation. In the case of HNE, levels of endogenous exposure are available, but it remains to be explored to what extent they originate from endogenous or exogenous sources. This will apply to other α,β-alkenals as well. In addition, personal habits such as smoking and physical exercise may influence the endogenously formed levels requiring further characterisation and quantification. For all α,β-alkenals, biomarkers need to be developed and validated to quantify endogenous exposure levels. For dependable dosimetry, stability and turnover of exposure biomarkers such as, for instance, protein and DNA adducts remain to be determined to better indicate what type of exposure, i.e. long versus short term, they reflect.

There is also a paucity of data on the oral bioavailability of free and protein-bound glycated amino acids and their adverse health effects. Structure-based studies in experimental animals and humans assessing the association between dietary intake of individual glycation compounds and biomarker levels as well as adverse effects are needed. To this end, application of isotopically labelled glycation compounds will be useful to accurately assess the added contribution of exogenous exposure to endogenously formed glycation compounds, including those produced from dietary sugars. Such studies are particularly crucial for reactive dicarbonyls, which from a toxicological perspective may be of most concern due to their known genotoxicity and potential carcinogenicity. Understanding how much exogenous exposure is needed to increase endogenous levels of biomarkers, most notably DNA adducts, may allow risk assessors to prioritise glycation compounds that humans are exposed to via food.

Similarly, there is scientific consensus that carcinogenic NOC can be formed endogenously from appropriate precursors. Data on urinary levels of *N*-nitrosated amino acids are available to some extent but they apparently depict exclusively gastric, acid catalysed *N*-nitrosation. However, endogenous formation of carcinogenic NOC may not be limited just to the gastrointestinal tract (the stomach representing the predominant reaction compartment).

Estimates of endogenous NDMA exposure from NDMA blood levels appear to be based on analytical data of uncertain reliability and, therefore, require confirmation and validation. Refined toxicokinetic modelling may lend further support, given blood levels reflect steady-state conditions in humans and can be measured reliably with the required precision and convincing proof that analytical artefacts are excluded.

In addition, development and validation of biomarkers that will allow a realistic estimate of systemic in vivo formation of carcinogenic NOC is required and needs to consider all relevant body compartments, including potential effects of the human microbiome(s).

To get a more comprehensive picture, it may as well be advisable to monitor a spectrum of biomarkers that address different body compartments and/or potential *N*-nitrosation processes. In addition, such measurements should encompass an adequate number of individuals at various lifestyle and health conditions to generate a database that depicts a probabilistic approach. A further possible way forward may be to exploit genome wide expression responses in animals and humans in an exposure related way. Altogether, this might open new research avenues towards a better understanding of the relation between endogenous nitrosation, exposure to nitrate/nitrite/NOx and NOC and potentially associated biological effects, especially potential health risks and benefits. The systemic endogenous NOC formation potential has not been evaluated quantitatively and comprehensively as a process of potential relevance to human cancer.

The potential endogenous formation of furans or furan-dependent metabolites represents a significant source of uncertainty. This is important for potential use of urinary biomarkers for exposure assessment. In terms of biomonitoring, the detection of endogenously formed metabolites excreted via urine may lead to an overestimation of external furan exposure via food and other sources and consequently to an overestimation of the related human risk. Discrimination between exogenous exposure and potential endogenous formation of furans and their reactive metabolites is urgently needed. For a safe application of a biomarker-based approach, the contribution of endogenous formation to the human exposome needs to be determined. Due to the high volatility of furans, a reliable estimation of human exposure cannot be based exclusively on consumption data and analytical determination of contents in food. Although NAcLys-BDA and mono GSH-BDA have already been used in biomonitoring as urinary biomarkers, information is still lacking regarding the discrimination between potentially endogenous formation and exogenous exposure. To address this question, in vivo studies with isotopically labelled furans are needed. Alternatively, duplicate diet studies in humans with dosimetry of dietary furan intake may also provide relevant information by comparing input of furans versus biomarker output, as already demonstrated, e.g. for acrylamide and acrolein (Goempel et al. 2017, 2019; Goerke et al. 2019; Ruenz et al. 2016). For animal experiments, background contamination of feed requires due attention when attempting reliable dose–response studies.

For some PRCs, endogenous formation is even less well characterised or even considered unlikely, as is the case for 2- and 3-MCPD and glycidyl esters. Up to now, no significant endogenous formation of 2- and 3-MCPD or their esters is known. Established levels in exogenous exposure suggest that potential future findings of endogenously produced 3- and 2-MCPD will only contribute marginally to the overall exposure estimates. For further proper risk assessment and a better monitoring of both exogenous and potential endogenous exposures, appropriate biomarkers are required that also differentiate between exposure to 2-MCPD- and 3-MCPD.

Further research is also needed to develop knowledge on endogenous formation of glycidol from food precursors. Currently, cigarette smoke is the only non-food source of glycidol that is mentioned in the literature. Abraham et al. have demonstrated a direct relationship between glycidol dietary intake and the level of 2,3-diHOPr-Val Hb adducts in humans and proposed a biomarker-based model to estimate dietary glycidol exposure (Abraham et al. 2019). The results appear promising but must be confirmed by more studies with higher numbers of subjects, to refine and validate the model. In addition, the specificity of 2,3-diHOPr-Val Hb adducts to reflect glycidol exposure needs to be ascertained. This is illustrated by background biomarker levels that appeared to indicate a mean total glycidol exposure considerably higher than current estimates for adults. Possible reasons were discussed, including other oral or inhalational glycidol sources or exposure to other chemicals forming the same adduct or endogenous formation, but were deemed not convincing, and therefore, further research was proposed, e.g. by investigating internal exposure of subjects on carefully controlled diets with established dosimetry of dietary glycidol uptake and balancing dietary glycidol input versus urinary biomarker output (Abraham et al. 2019).

Taken together, the current available evidence allows to conclude that for several PRCs endogenous formation appears to contribute substantially to the exposome, in some cases even in such a way that it outweighs the exogenous exposure. This needs to be taken into account for adequate risk assessment. However, given the limitations in the current data base for several compounds additional information is required, including:

- Identification of biomarkers and/or methodology that enable quantification of exposure and discrimination of exogenous versus endogenous origin of the overall exposome.
- Further elucidation of endogenous formation pathways, including the contribution of the human microbiome.
- Quantification of interspecies and interindividual differences in these processes.
- Establishment of compound-specific exposure biomarkers.
- Building an extended database, based on dependable dosimetry of total PRC-related exposure, with the aim to achieve probabilistic estimates of total PRC-related exposure of endogenous versus exogenous origin.
- Given the complex human endogenous and exogenous exposure to PRCs, design of better-informed epidemiologic studies, encompassing appropriate biomarker dosimetry and taking into account the respective endogenous exposome.
- Exposome based health risk assessment of PRC-related human exposure.
- Research on mitigation of exogenous, if applicable also of endogenous exposure.
- Determination of the potentially unavoidable endogenous background levels as reference informing mitigation of exogenous exposure.

To conclude, it is recommended that regulatory bodies develop generally accepted methodology how to balance risks associated with endogenous exposures against that from exogenous sources. Several of the examples presented already reveal that endogenous sources constitute a substantial part of the exposome with potentially major influence on risk assessment.

## Abbreviations

2,3-diHOPr-Val: *N*-(2,3-Dihydroxypropyl)-valine
2-MCPD: 2-Monochloropropane-1,3-diol
3-HMPA: 3-Hydroxypropylmercapturic acid
3-HPA: 3-Hydroxypropanal
3-MCPD: 3-Monochloropropane-1,2-diol
5-HMF: 5-Hydroxymethylfurfural
7-CEGua: 7-Carboxyethyl-guanosine
AA: Acrylamide
AAMA: *N*-Acetyl-*S*-(2-carboxamidoethyl) cysteine
AC: Acrolein
ADH5: Alcohol dehydrogenase 5
AGE: Advanced glycation end product
ALDH2: Aldehyde dehydrogenase 2
AUC: Area under the ROC curve
BAT: Biologische Arbeitsstoff-Toleranzwerte (biological tolerance values)
BDA: Cis-2-butene-1,4-dial
BDML10: Lower confidence limit of the benchmark dose for a 10% response
BfR: German Federal Institute for Risk Assessment
BMD: Benchmark dose
BMI: Body mass index
bw: Body weight
CEdA: *N*^6^-(1-Carboxyethyl)-2′-deoxyadenosine
CEdG: *N*^2^-(1-Carboxyethyl)-2′-deoxyguanosine
CEMA: 2-Carboxyethylmercapturic acid
CML: Carboxymethyllysine
CONTAM panel: EFSA Panel on Contaminants in the Food Chain
dA: Deoxyadenosine
DAG: Diacylglycerols
DFG: German Research Foundation
dG: Deoxyguanosine
DHMPA: 2,3-Dihydroxypropyl mercapturic acid
DHU: Dihydrouracil
ECHA: European Chemicals Agency
EFSA: European Food Safety Authority
EO: Ethylene oxide (oxirane, ethene oxide)
EPA: U.S. Environmental Protection Agency
ETS: Environmental tobacco smoke
FL: *N*-*ε*-Fructoselysine
G(E): Glycidol and its esters
GA: Glycidamide
GAMA: *N*-Acetyl-*S*-(3-amino-2-hydroxy-3-oxopropyl)-l-cysteine
GC: Gas chromatography
GE: Glycidyl fatty acid esters
GSH: Glutathione
GSTT1-1: Glutathione-S-transferase T1-1
Hb: Haemoglobin
HbA1C: *β*-*N*-1-(Deoxyfructosyl) valine
HESI: Health and Environmental Sciences Institute
HHE: 4-Hydroxy-2-hexenal
HNE: 4-Hydroxy-2-nonenal
HOE: 4-Hydroxy-2-octenal
HPLC: High-pressure liquid chromatography
HPMA: *N*-Acetyl-*S*-(3-hydroxypropyl)-l-cysteine
IARC: International Agency of Research for Cancer
ILSI: International Life Science Institute
iso-GAMA: *N*-Acetyl-*S*-(1-carbamoyl-2-hydroxyethyl)-l-cysteine
JEFCA: Joint FAO/WHO Expert Committee on Food Additives
LD50: Lethal dose causing death of 50% of a group of test animals
MA: Mercapturic acid
MAG: Monoacylglycerols
MAK: Maximale Arbeitsplatz-Konzentration (maximum workplace concentration)
MG-H1: *N*^δ^-(5-Hydro-5-methyl-4-imidazolon-2-yl)-l-ornithine
MOE: Margin of exposure
MRL: Maximum residue limit
MS: Mass spectroscopy
NAcCys-BDA-NAcLys: *N*-Acetyl-*S*-(1-(5-acetylamino-5-carboxypentyl)-1H-pyrrol-3-yl)-l-cysteine
NAcLys-BDA: *R*-2-Acetylamino-6-(2,5-dihydro-2-oxo-1H-pyrrol-1-yl)-1-hexanoic acid
NDEA: *N*-Nitroso-diethylamine
NDELA: *N*-Nitrosodiethanolamine
N-DHU: *N*-Nitrosodihydrouracil
NDMA: *N*-Nitroso-dimethylamine
NDPA: *N*-Nitroso-morpholine-di-2-hydroxypropylamine
NF-κB: Nuclear factor ‘kappa-light-chain-enhancer’ of activated B cells
NHS: National Health Service (UK)
NMOR: *N*-Nitroso-morpholine
NNAL: 4-*N*-Methyl-*N*-nitroso-4-(3-pyridyl)butanal
NNK: 4-*N*-Methyl-4-(1 pyridyl)-1-butanone
NNN: *N*-Nitroso-nornicotine
NO: Nitrogen monoxide
NOAEL: No-observed-adverse-effect-level
NOC: *N*-Nitroso compounds
NOx: Nitrogen oxides
NPRO: *N*-Nitrosoproline
NTP: United States National Toxicology Program
PhIP: 2-Amino-1-methyl-6-phenylimidazo[4,5-b]pyridine
PhIP-M1: 7-Hydroxy-5-methyl-3-phenyl-6,7,8,9-tetrahydropyrido[3′,2′:4,5]imidazo[1,2-a]pyrimidin-5-ium chloride
PRCs: Process-related contaminants
PUFA: Polyunsaturated fatty acids
RAGE: Advanced glycation end product receptor
SKML: DFG Senate Commission on Food Safety (DE)
T25 dose: Dose that results in a 25% increase above background in lifetime tumour incidence
TDI: Tolerable daily intake
TEA: Thermal energy analyzer
WHO: World Health Organisation
